# Early modulation of macrophage ROS-PPARγ-NF-κB signalling by sonodynamic therapy attenuates neointimal hyperplasia in rabbits

**DOI:** 10.1038/s41598-020-68543-9

**Published:** 2020-07-15

**Authors:** Jianting Yao, Xuezhu Zhao, Fancheng Tan, Xiaoru Cao, Shuyuan Guo, Xiang Li, Zhen Huang, Kamal Diabakte, Lu Wang, Mingyu Liu, Zhaoqian Shen, Bicheng Li, Zhengyu Cao, Siqi Sheng, Minqiao Lu, Yang Cao, Hong Jin, Zhiguo Zhang, Ye Tian

**Affiliations:** 1 Department of Cardiology, The First Affiliated Hospital, Cardiovascular Institute, Harbin Medical University, 23 Youzheng Street, Harbin, 150001 China; 2 Department of Pathophysiology and Key Laboratory of Cardiovascular Pathophysiology, Harbin Medical University, Key Laboratory of Cardiovascular Medicine Research (Harbin Medical University), Ministry of Education, Harbin, China; 3 Medicine Department, Karolinska Institute, Stockholm, Sweden; 4 Laboratory of Photo- and Sono-Theranostic Technologies and Condensed Matter Science and Technology Institute, Harbin Institute of Technology, Harbin, China; 5 Heilongjiang Academy of Medical Sciences, Harbin, China

**Keywords:** Apoptosis, Restenosis

## Abstract

Disruption of re-endothelialization and haemodynamic balance remains a critical side effect of drug-eluting stents (DES) for preventing intimal hyperplasia. Previously, we found that 5-aminolevulinic acid-mediated sonodynamic therapy (ALA-SDT) suppressed macrophage-mediated inflammation in atherosclerotic plaques. However, the effects on intimal hyperplasia and re-endothelialization remain unknown. In this study, 56 rabbits were randomly assigned to control, ultrasound, ALA and ALA-SDT groups, and each group was divided into two subgroups (n = 7) on day 3 after right femoral artery balloon denudation combined with a hypercholesterolemic diet. Histopathological analysis revealed that ALA-SDT enhanced macrophage apoptosis and ameliorated inflammation from day 1. ALA-SDT inhibited neointima formation without affecting re-endothelialization, increased blood perfusion, decreased the content of macrophages, proliferating smooth muscle cells (SMCs) and collagen but increased elastin by day 28. In vitro*,* ALA-SDT induced macrophage apoptosis and reduced TNF-α, IL-6 and IL-1β via the ROS**-**PPARγ**-**NF-κB signalling pathway, which indirectly inhibited human umbilical artery smooth muscle cell (HUASMC) proliferation, migration and IL-6 production. ALA-SDT effectively inhibits intimal hyperplasia without affecting re-endothelialization. Hence, its clinical application combined with bare-metal stent (BMS) implantation presents a potential strategy to decrease bleeding risk caused by prolonged dual-antiplatelet regimen after DES deployment.

For the past few decades, percutaneous coronary intervention (PCI) has been developed as a routine treatment modality for coronary atherosclerotic disease. In the era of balloon angioplasty and bare-metal stents (BMS), the major problem of PCI is intimal hyperplasia resulting in restenosis of the treated vessel, which leads to renewed symptoms and the need for repeated intervention. The antiproliferative drugs released from drug-eluting stents (DES) inhibit intimal hyperplasia but delay re-endothelialization at the expense of an increase in late stent thrombosis. Prolonged dual-antiplatelet therapy diminishes the rate of stent thrombosis at the cost of increased risk of bleeding^1–4^. Therefore, an optimal strategy for reducing in-stent intimal hyperplasia and restenosis without affecting re-endothelialization is urgently needed to maintain the balance between restenosis and haemostasis.

Inflammation drives the cascade of intimal hyperplasia^5^. After endothelium injury, infiltrated macrophages express numerous cytokines to initiate the proliferation and migration of smooth muscle cells (SMCs), leading to intimal hyperplasia^6,7^. A previous study provided evidence that inactivation and depletion of macrophages by systemic administration of liposome-encapsulated clodronate, with no adverse effect on endothelial cells, suppresses SMC proliferation and intimal hyperplasia in hypercholesterolemic rabbits and rats^8^. However, systemic inhibition of macrophage-mediated inflammation carries the risk of immunosuppression and fatal infection^9^. Thus, a rational and practical site-specific approach to reducing intimal hyperplasia by eliminating macrophages is needed.

Sonodynamic therapy (SDT) is a non-invasive technique characterized by the application of ultrasound to locally activate sono-sensitizers and stimulate the production of reactive oxygen species (ROS) to modulate cell function or fate^10^. In our previous study, we found that 5-aminolevulinic acid-mediated sonodynamic therapy (ALA-SDT) induced macrophage apoptosis via the mitochondria-caspase pathway^11^. Moreover, ALA-SDT reduced IL-6 and TNF-α released by macrophages, leading to advanced atherosclerotic plaque inflammation resolution without off-target effects^12^. Therefore, in this study, we aimed to further investigate whether early intervention with ALA-SDT could ameliorate inflammation-driven intimal hyperplasia and its effect on re-endothelialization in balloon-denuded hypercholesterolemic rabbits.

It has been confirmed that inactivation of NF-κB signalling efficiently suppresses intimal hyperplasia^13^. A plethora of pro-inflammatory cytokines (TNF-α, IL-6, IL-1β) and anti-apoptotic proteins (Bcl-2, Bcl-xl) are mainly regulated by NF-κB activation and nuclear translocation^14,15^. Thus, in the present study, we observed the effects of ALA-SDT on human umbilical artery SMCs (HUASMCs) in a co-cultured cell system with THP-1-derived macrophages to address the underlying mechanisms of ALA-SDT therapy.

## Results

### Plasma total cholesterol

**Figure 1:**
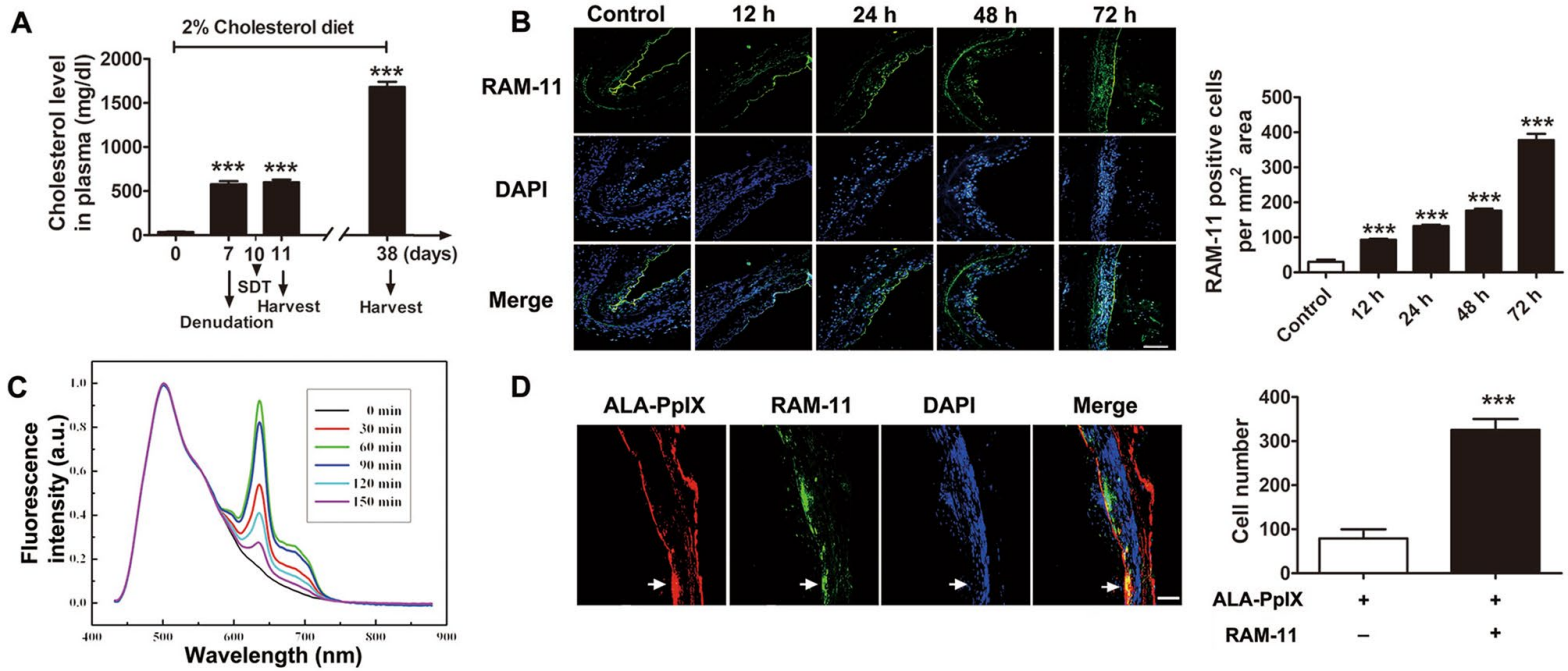
Rabbit experiment protocol, time course of macrophage accumulation, fluorescence intensity and cellular location of ALA-PpIX in the injured artery wall. (**A**) Schematic diagram and plasma cholesterol level in rabbit models. (**B**) Immunofluorescence analysis detected the variation in macrophage accumulation in the artery wall at 12, 24, 48 and 72 h after balloon denudation. Non-injured rabbits were used as controls. n = 5 at each time point. Scale bar = 100 μm. (**C**) ALA-PpIX fluorescence spectra in the injured arteries at 72 h after balloon denudation. Under the excitation of 405 nm laser light, two typical fluorescence emission bands centred at 635 nm and 690 nm were observed. The fluorescence spectra of PpIX were recorded at 0, 30, 60, 90, 120, and 150 min post-ALA (60 mg/kg) intravenous injection. Three experiments were conducted independently, and representative spectra are shown. (**D**) Cellular localization of ALA-PpIX in the media of the injured artery at 72 h after balloon denudation. At 1 h after ALA intravenous injection, ALA-PpIX fluorescence was mainly observed within the injured vascular media containing macrophages (stained with RAM-11). Scale bar = 100 μm. *ALA* 5-aminolevulinic acid, *PpIX* protoporphyrin. ***p < 0.001 versus 0 day or control.

Rabbit cholesterol levels increased from 35.15 ± 5.30 mg/dl on day 0 to 576.8 ± 35.24 mg/dl on day 7 when fed a hypercholesterolemic diet. On day 10, 3 days after balloon injury, the rabbits received different treatments. The cholesterol level increased to 599.8 ± 28.89 mg/dl on day 11 and 681 ± 60.55 mg/dl on day38 (Fig. 1A).

### Macrophage infiltration in the media of injured arteries

RAM-11-positive macrophages were determined by immunofluorescence in uninjured arteries (control group) and injured arteries harvested at 12, 24, 48 and 72 h after balloon denudation. The number of macrophages was increased by 3.1-fold at 12 h (93 ± 3), 4.4-fold at 24 h (132 ± 4), 5.9-fold at 48 h (176 ± 5) and 12.6-fold at 72 h (378 ± 18) in the media of balloon-denuded arteries compared with the control group (30 ± 6) (Fig. 1B).

### ALA-PpIX fluorescence detection

ALA was intravenously administered at 72 h after balloon denudation, and two fluorescence bands, one centred at 635 nm and the other at 690 nm, were detected conforming to the emission spectrum of PpIX under 405 nm light excitation. Typical ALA-PpIX fluorescence spectra were measured at 30 min intervals from 0 to 150 min post-ALA administration. The results showed that PpIX fluorescence intensity reached a maximum at 60 min and then decreased with time (Fig. 1C). The rabbits that received PBS injection showed no PpIX fluorescence under excitation. Thus, 60 min was specified as the ALA loading time in the following experiments.

### Cellular localization of ALA-PpIX

The cellular location of ALA-PpIX in the arterial media at 72 h after balloon denudation is shown in Fig. 1D. One hour after ALA injection, fluorescence microscopy showed that the number of ALA-PpIX-positive macrophages was 3.1-fold higher than the total number of other cells positive for ALA-PpIX (325 ± 14 vs. 79 ± 12) in media from the regions of the injured vascular.

### ALA-SDT induces macrophage apoptosis and reduces the expression levels of TNF-α, IL-6 and IL-1β in injured arteries

**Figure 2:**
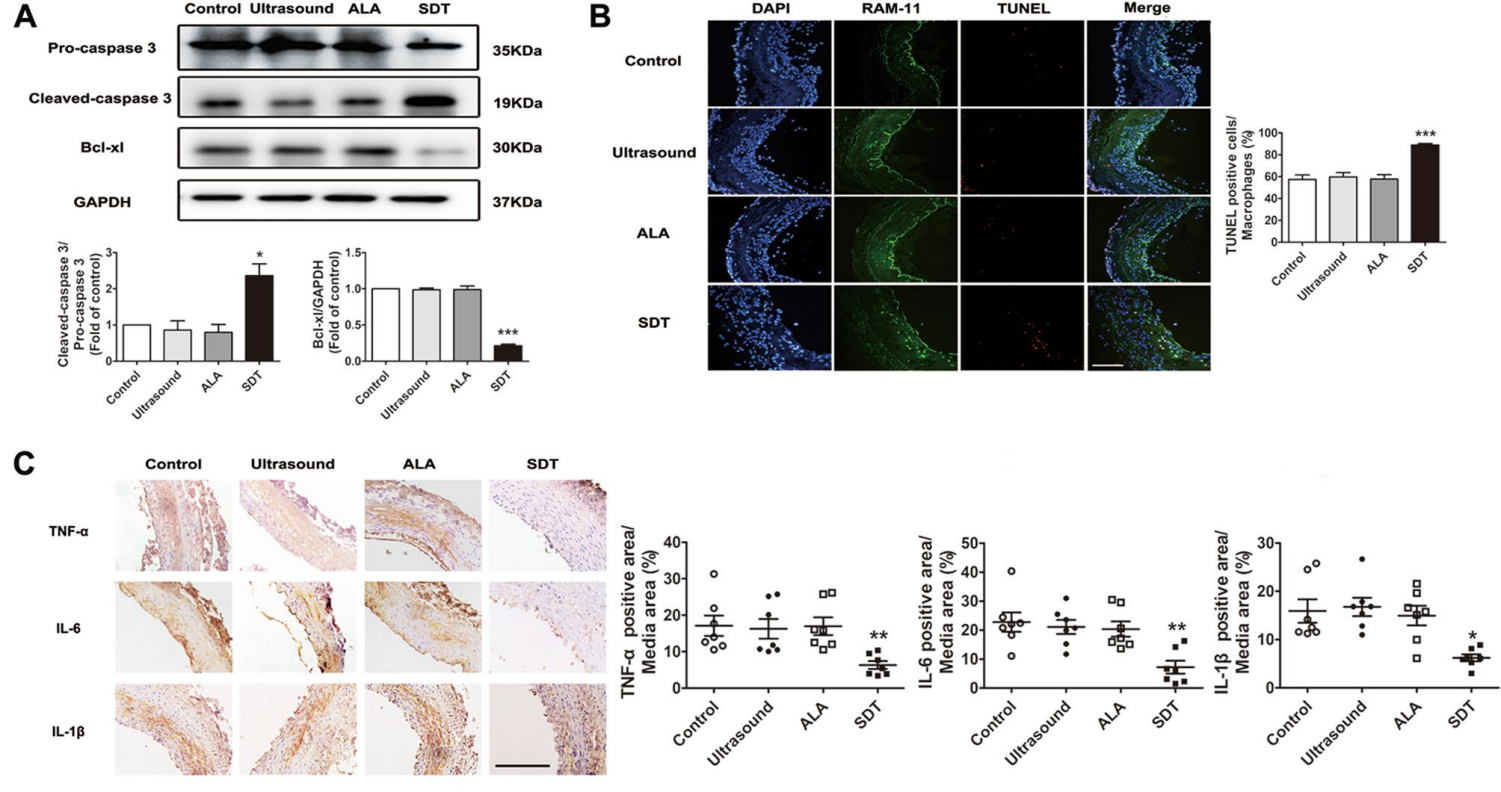
ALA-SDT induces macrophage apoptosis and reduces TNF-α, IL-6 and IL-1β expression in the media of balloon-denuded rabbit femoral arteries at day 1 after treatment. (**A**) Rabbit tissue Western blot analysis of apoptosis-related proteins, including pro-caspase 3, cleaved caspase-3 and Bcl-xl. GAPDH was used as a loading control. (**B**) Immunofluorescence staining of TUNEL and RAM-11 to assess the macrophage apoptosis rate in the indicated groups (n = 6). Scale bar = 100 μm. (**C**) Representative sections of TNF-α, IL-6 and IL-1β expression stained by immunohistochemistry and quantitative analysis in the indicated groups (n = 7). Scale bar = 200 μm. Each data point represents one rabbit. *p < 0.05, **p < 0.01 and ***p < 0.001 versus control.

On day 1 after ALA-SDT, the protein levels of cleaved caspase-3 and Bcl-xl significantly increased by 136% (1.00 vs. 2.36 ± 0.33) and decreased by 79% (1.00 vs. 0.21 ± 0.02), respectively (Fig. 2A), while the macrophage apoptosis rate significantly increased by 55% (Fig. 2B) compared with the control group (57.37 ± 4.28% vs. 88.86 ± 1.39%). Meanwhile, in the arterial media, we observed reductions in TNF-α by 63% (17.10 ± 2.80% vs. 6.33 ± 1.08%), in IL-6 by 68% (22.76 ± 3.36% vs. 7.18 ± 2.21%) and in IL-1β by 61% (15.91 ± 2.42% vs. 6.22 ± 0.72%) (Fig. 2C).

### ALA-SDT inhibits neointima formation

**Figure 3:**
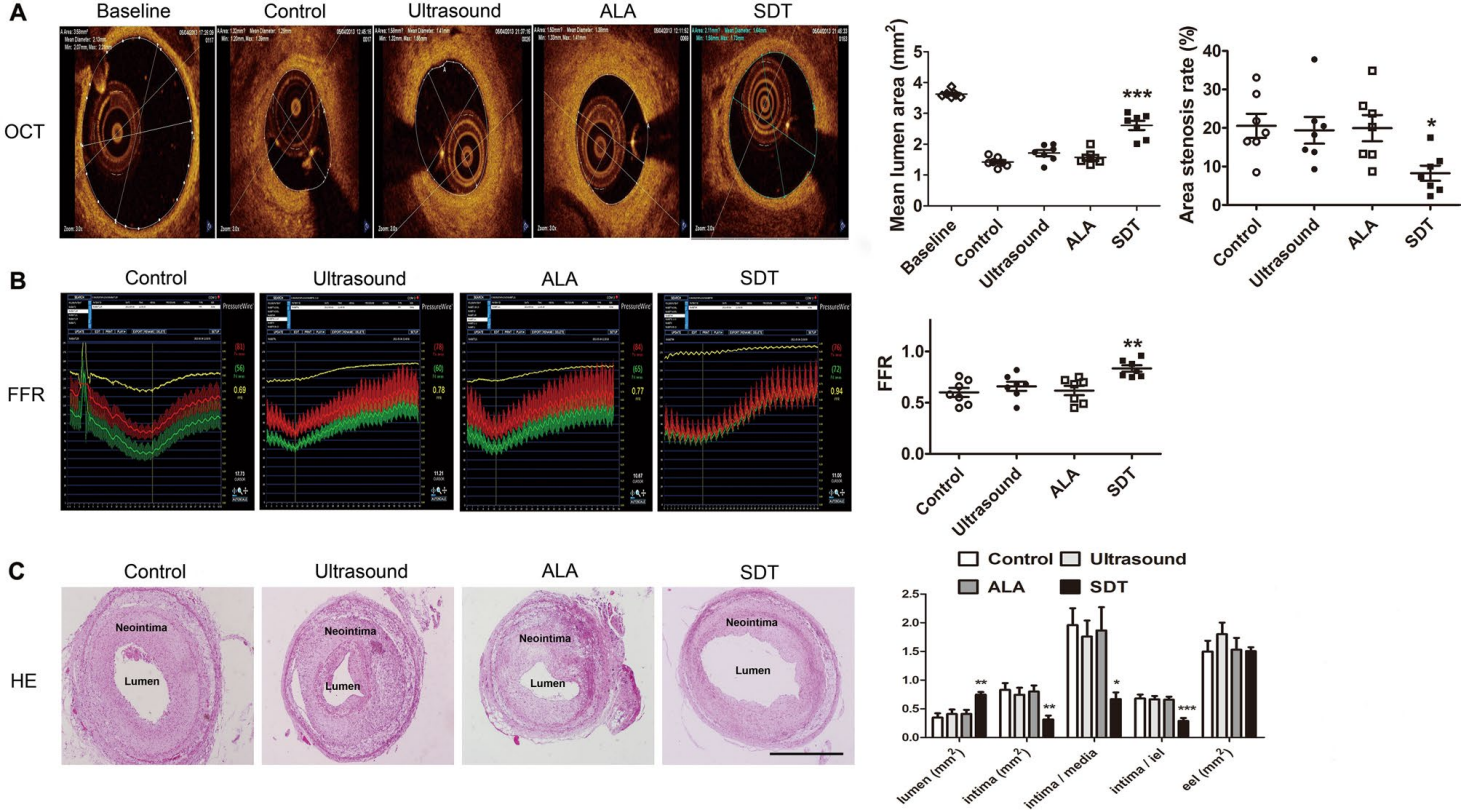
ALA-SDT inhibits intimal hyperplasia in the rabbit femoral arteries at day 28 after treatment. (**A**) Representative cross-sections of OCT scanning and quantitative analysis of the mean lumen area and area stenosis in the indicated groups (n = 7). (**B**) Representative images of FFR and quantitative analysis of FFR values in the indicated group (n = 7). (**C**) Representative sections of HE staining and quantitative analysis of lumen area, intimal area, intima/media ratio, intima/IEL and EEL area in the indicated groups (n = 7). Each data point represents one rabbit. *OCT* optical coherence tomography, *Baseline* normal healthy rabbits, *FFR* fractional flow reserve, *IEL* internal elastic lamina, *EEL* external elastic lamina. Scale bar = 1 mm. *p < 0.05, **p < 0.01 and ***p < 0.001 versus control.

At day 28 after ALA-SDT treatment, an angiogram showed that the lumen diameter of the arteries in the SDT group was larger than the lumen diameters in the other groups (Supplementary Fig. S1). Meanwhile, compared with the control group, the mean lumen area and the percentage of area stenosis measured by optical coherence tomography (OCT) in the SDT group were significantly increased by 83% (1.42 ± 0.06 vs. 2.61 ± 0.15 mm^2^) and decreased by 60% (20.56 ± 3.12% vs. 8.24 ± 1.97%), respectively (Fig. 3A). Fractional flow reserve (FFR), which measures the pressure gradient across stenotic arteries, was increased in arteries with larger cross-sectional lumen areas^16,17^. The FFR value in the SDT group (Fig. 3B) increased significantly by 0.39-fold compared with the control group (0.60 ± 0.04 vs. 0.83 ± 0.03). Morphometric analysis indicated that intimal growth-expressed either as intimal area, intima/media, or intima/internal elastic lamina (IEL)-was significantly reduced upon ALA-SDT [by 62% (0.83 ± 0.12 vs. 0.31 ± 0.07 mm^2^), 66% (1.96 ± 0.29 vs. 0.67 ± 0.12), and 58% (68.11 ± 6.75% vs. 28.79 ± 5.22%), respectively], resulting in a significant increase in luminal area [by 116% (0.35 ± 0.07 vs. 0.75 ± 0.04 mm^2^)] compared with the control group (Fig. 3C).

### ALA-SDT alters neointima composition

**Figure 4:**
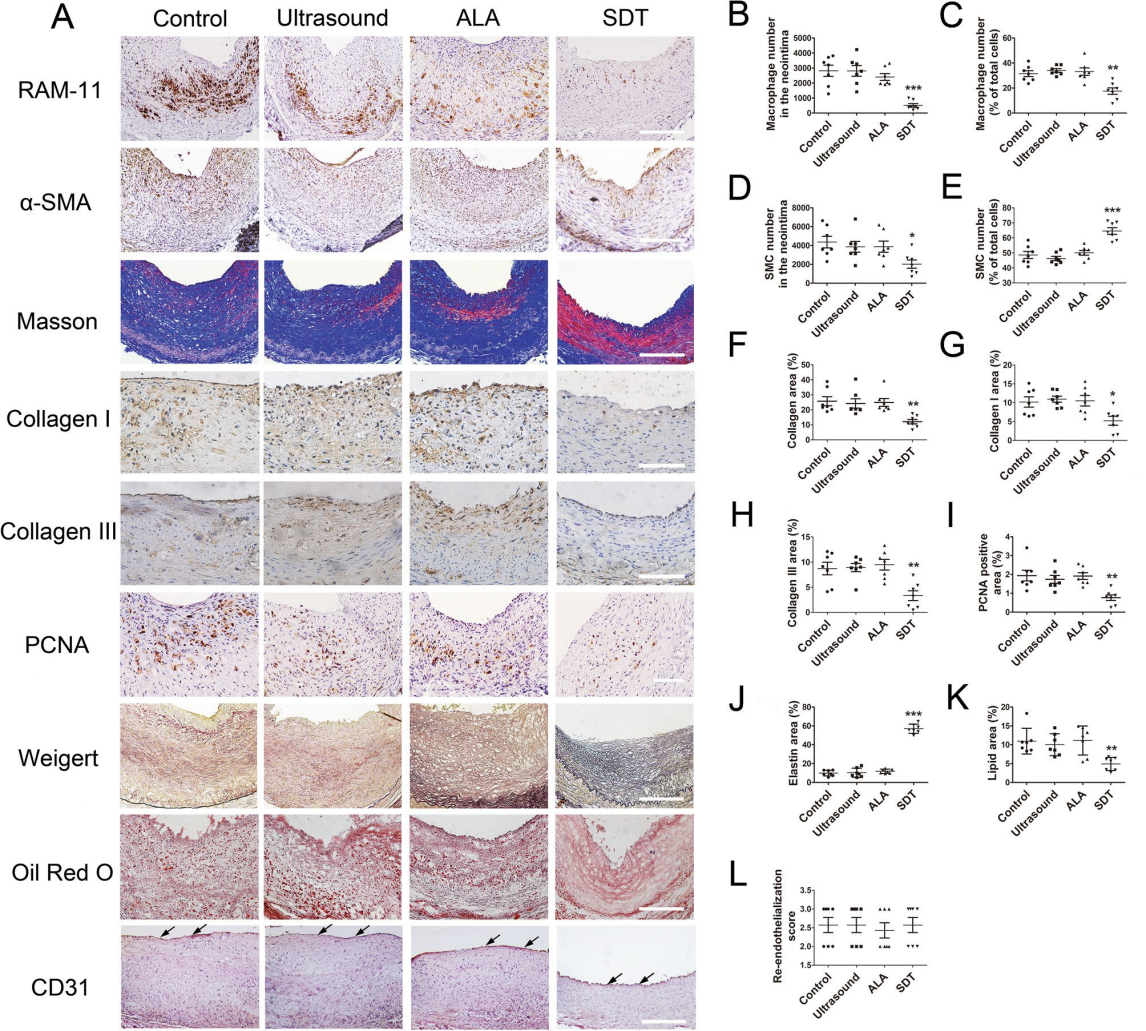
ALA-SDT alters the composition of neointima in the rabbit femoral arteries at day 28 after treatment. (**A**) Representative sections stained for macrophages (RAM-11), smooth muscle cells (α-SMA), collagen (Masson), collagen I, collagen III, PCNA, elastin (Weigert), lipids (Oil Red O) and endothelium (CD31) in the indicated groups. PCNA, proliferating cell nuclear antigen. Black arrows indicate the regenerated endothelium. Scale bar = 250 μm. (**B**–**L**) Quantification analysisin the indicated groups (n = 7). Each data point represents one rabbit. *p < 0.05, **p < 0.01and ***p < 0.001 versus control.

On day 28 after treatment, compared with the control group, ALA-SDT notably suppressed neointima formation and significantly decreased the numberof macrophages and SMCs in the neointima (Fig. 4B,D). However, although the percentage ratio of macrophages in the SDT group decreased by 44.2% (31.49 ± 2.38% vs. 17.55 ± 2.59%), the ratio of SMCs to total cells increased by 32.9% (48.61 ± 2.35% vs. 64.58 ± 2.22%) (Fig. 4C,E). Moreover, ALA-SDT significantly decreased the content of collagen by 53% (25.79 ± 3.00% vs. 12.15 ± 1.49%), collagen I by 49% (10.19 ± 1.37% vs. 5.21 ± 1.17%),collagen III by 62% (8.72 ± 1.25% vs. 3.33 ± 0.98%), PCNA-positive cells by 60% (1.94 ± 0.28% vs. 0.77 ± 0.15%) and lipids by 55% (10.97 ± 1.29% vs. 4.92 ± 0.66%), while it increased elastin content by 4.7-fold (9.99 ± 1.15% vs. 57.11 ± 1.84%) in the neointima compared with the control group (Fig. 4F–K). Notably, the re-endothelialization score indicated by CD31-positive endothelium coverage was similar in all four groups (Fig. 4L). Analysis of serial sections revealed that the majority of PCNA-positive cells were SMCs (Supplementary Fig. S2). On day 28 after ALA-SDT,the expression levels of ICAM-1 and MCP-1 were significantly decreased by 51% (21.79 ± 2.71% vs. 10.70 ± 1.28%) and 52% (19.37 ± 1.77% vs. 9.32 ± 2.39%), respectively, compared with the control group (Supplementary Fig. S3).

### ALA-SDT inhibits NF-κB signalling and subsequently suppresses the expression and secretion of TNF-α, IL-6 and IL-1β in THP-1-derived macrophages

**Figure 5:**
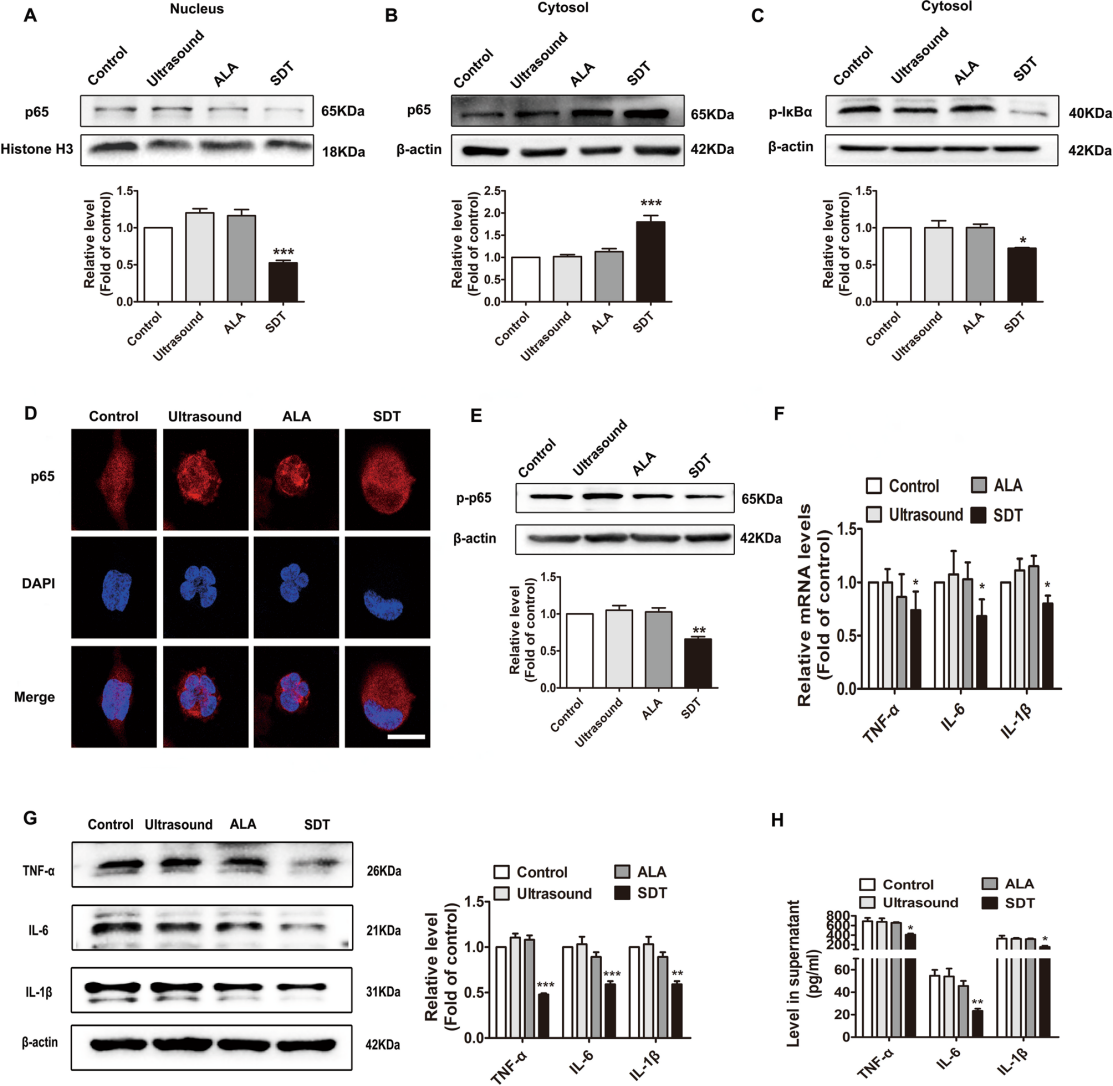
ALA-SDT inhibits NF-κB signalling and subsequently suppresses the expression and secretion of TNF-α, IL-6 and IL-1β in THP-1-derived macrophages. Western blot analysis of the nuclear protein expression of NF-κB p65 (**A**) and the cytoplasmic protein expression of NF-κB p65 (**B**) and p-IκBα (**C**). Histone H3 and β-actin were used as loading controls for nuclear proteins and cytoplasmic proteins, respectively. (**D**) Immunofluorescence analysis of NF-κB p65 nuclear translocation. Scale bar = 20 μm. (**E**) Western blot analysis of p-p65 levels. (**F**) Quantitative real-time polymerase chain reaction analysis of mRNA levels of TNF-α, IL-6 and IL-1β. (**G**) Western blot analysis of TNF-α, IL-6 and IL-1β. (**H**) ELISA analysis of TNF-α, IL-6 and IL-1β in supernatants (n = 5). *p < 0.05, **p < 0.01 and ***p < 0.001 versus control.

To explore the underlying mechanisms by which ALA-SDT inhibited inflammation in the injured arteries, we performed Western blot analysis and found that the expression level of NF-κB p65 in THP-1-derived macrophages was significantly decreased in the nucleus and increased in the cytoplasm after ALA-SDT (Fig. 5A,B). IκBα phosphorylation was significantly decreased upon ALA-SDT treatment, suggesting an inhibitory effect on IκBα degradation that aids in sequestering NF-κB in the cytoplasm (Fig. 5C). The inhibitory effect of ALA-SDT on p65 nuclear translocation was further confirmed by confocal laser scanning (Fig. 5D). Furthermore, ALA-SDT significantly reduced the phosphorylation level of NF-κB p65 (p-p65) (Fig. 5E), which is considered a marker of NF-κBactivation. As shown in Fig. 5F–H, ALA-SDT prominently inhibited the expression and secretion of TNF-α, IL-6 and IL-1β in THP-1-derived macrophages.

### ALA-SDT-treated THP-1-derived macrophages inhibit HUASMC proliferation, migration and IL-6 expression

The fluorescence intensity of ALA-PpIX in TNF-α-stimulated HUASMCs gradually increased from 0 to 24 h (Supplementary Fig. S4A). When we treated TNF-α-stimulated HUASMCs with the same SDT parameters to induce macrophage apoptosis, we did not find any remarkable change in TNF-α, IL-6 or IL-1β secretion (Supplementary Fig. S4B). Intriguingly, when ALA-SDT-treated THP-1-derived macrophages were co-cultured with TNF-α-stimulated HUASMCs, remarkable inhibition of HUASMC proliferation, migration and IL-6 production was observed (Supplementary Fig. S4C–G).

### ALA-SDT inhibits NF-κB transcriptional activity while increasing PPARγ transcriptional activity in THP-1-derived macrophages

To clarify the mechanism by which ALA-SDT inhibited NF-κB signalling, we performed a transcription factor profiling array. Among the 96 transcription factors, PPAR was significantly up-regulated, and HIF, NF-κB and SP-1 were significantly down-regulated by ALA-SDT (Supplementary Fig. S5). Compared with the control group, ALA-SDT reduced NF-κB binding to DNA response elements by 66% (3.99 ± 0.86 vs. 1.36 ± 0.23) while increasing PPAR binding to DNA response elements by 182% (6.41 ± 1.52 vs. 18.10 ± 3.60). We further confirmed that ALA-SDT increased the protein level of PPARγ but not PPARα or PPARβ/δ in THP-1-derived macrophages (Supplementary Fig. S6).

### ALA-SDT inhibits NF-κB signalling and the production of TNF-α, IL-6 and IL-1β by activating PPARγ

**Figure 6:**
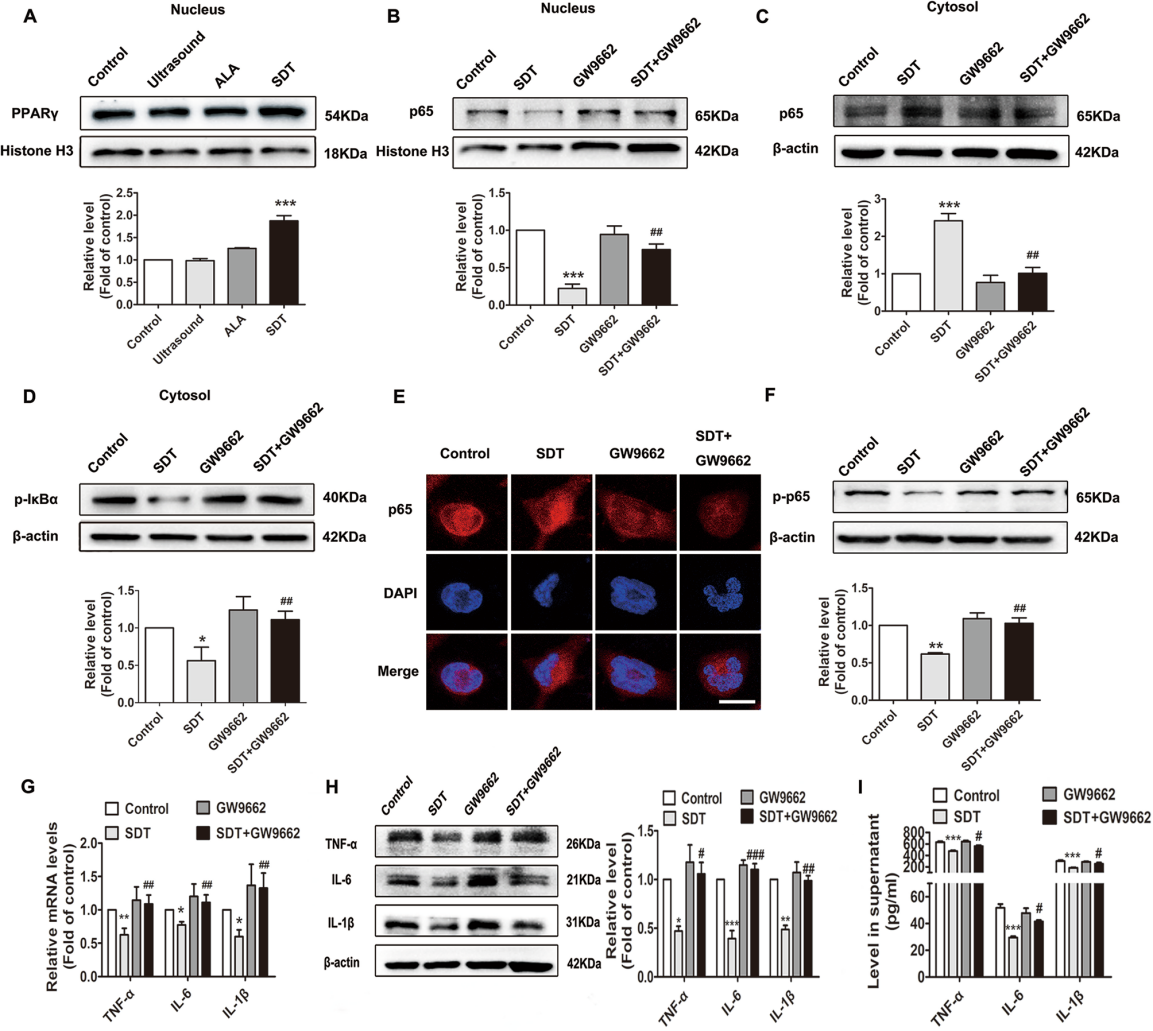
ALA-SDT inhibits NF-κB signalling and reduces the production of TNF-α, IL-6 and IL-1β by activating PPARγ in THP-1-derived macrophages. (**A**) Western blot analysis of the nuclear protein level of PPARγ. Cells were pre-treated with GW9662 for 1 h, followed by ALA-SDT treatment. Western blot analysis of the nuclear protein expression of NF-κB p65 (**B**) and the cytoplasmic protein expression of NF-κB p65 (**C**) and p-IκBα (**D**). (**E**) Immunofluorescence analysis of NF-κB p65 nuclear translocation. Scale bar = 20 μm. (**F**) Western blot analysis of the p-p65 level. (**G**) Quantitative real-time polymerase chain reaction analysis of mRNA levels of TNF-α, IL-6 and IL-1β. (**H**) Western blot analysis of TNF-α, IL-6 and IL-1β. (**I**) ELISA analysis of TNF-α, IL-6 and IL-1β in supernatants (n = 5). *p < 0.05, **p < 0.01 and ***p < 0.001 versus control. ^#^*p* < 0.05, ^##^*p* < 0.01 and ^###^*p* < 0.001 versus SDT.

PPARγ has been generally identified as an anti-inflammatory transcription factor. A part of this anti-inflammatory regulation is mediated through negative interference with NF-κB^18^. In this study, ALA-SDT significantly increased the nuclear protein level of PPARγ in THP-1-derived macrophages (Fig. 6A). To determine whether ALA-SDT inhibits NF-κB signalling and if the production of TNF-α, IL-6 and IL-1β occurs through PPARγ activation, THP-1-derived macrophages were pre-treated with the well-known PPARγ inhibitor GW9662 or PPARγ siRNA. The inhibition of NF-κB signalling by ALA-SDT was reversed by the presence of GW9662 (Fig. 6B–F) or PPARγ siRNA (Supplementary Fig. S8A–D). Moreover, ALA-SDT-induced TNF-α, IL-6 and IL-1β reduction was also reversed by GW9662 (Fig. 6G–I) or PPARγ siRNA (Supplementary Fig. S8E–G).

### *ALA-SDT induces THP-1-derived macrophage apoptosis *via* the PPARγ-NF-κB pathway*

**Figure 7:**
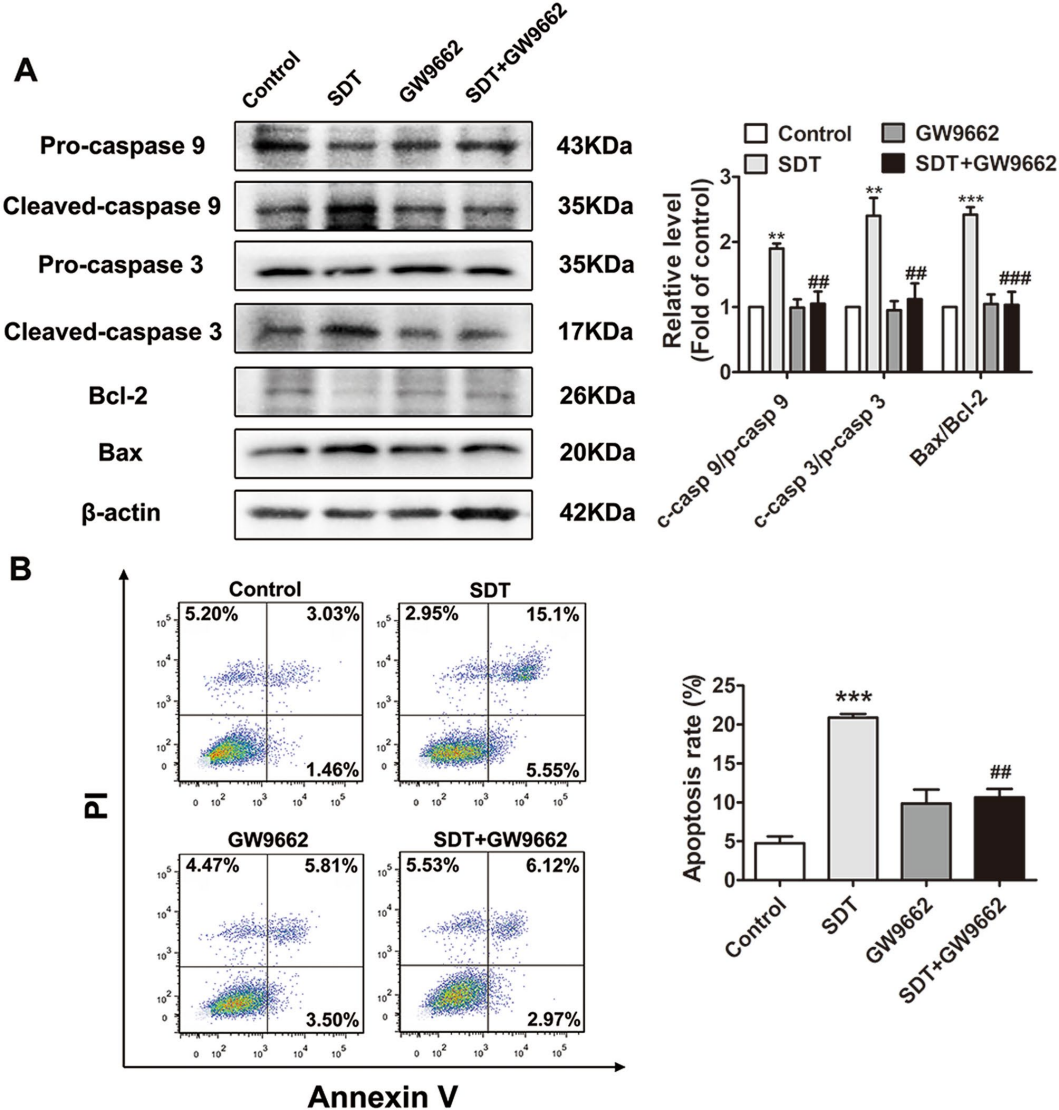
ALA-SDT induces THP-1-derived macrophage apoptosis via PPARγ-NF-κB signalling. Cells were pre-treated with GW9662 for 1 h, followed by ALA-SDT treatment. (**A**) Western blot analysis of apoptosis-related proteins, including pro-caspase-9, cleaved caspase-9, pro-caspase-3, cleaved caspase-3, Bcl-2 and Bax. (**B**) THP-1-derived macrophages treated with ALA-SDT in the presence or absence of GW9662 were stained with Annexin V–FITC/PI, followed by flow cytometry analysis of apoptosis. **p < 0.01 and ***p < 0.001 versus control. ^##^*p* < 0.01 and ^###^*p* < 0.001 versus SDT.

As an upstream regulatorof NF-κB, PPARγ is also involved in the regulation of apoptosis^19^. To illustrate whether PPARγ-NF-κB signalling is involved in the ALA-SDT-induced apoptosis process, we detected the effects of ALA-SDT on apoptosis-related protein expression, including pro-caspase-9, cleaved caspase-9, pro-caspase 3, cleaved caspase-3, Bcl-2 and Bax, in the presence and absence of GW9662 in THP-1-derived macrophages. Here, we demonstrated that pretreatment with GW9662 reversed the effect of ALA-SDT on apoptosis-related protein expression (Fig. 7A). Furthermore, flow cytometry analysis indicated that GW9662 pretreatment diminished the increased percentage of apoptotic cells upon ALA-SDT (Fig. 7B).

### *ALA-SDT regulates the PPARγ-NF-κB signalling pathway *via* ROS production in THP-1-derived macrophages*

**Figure 8:**
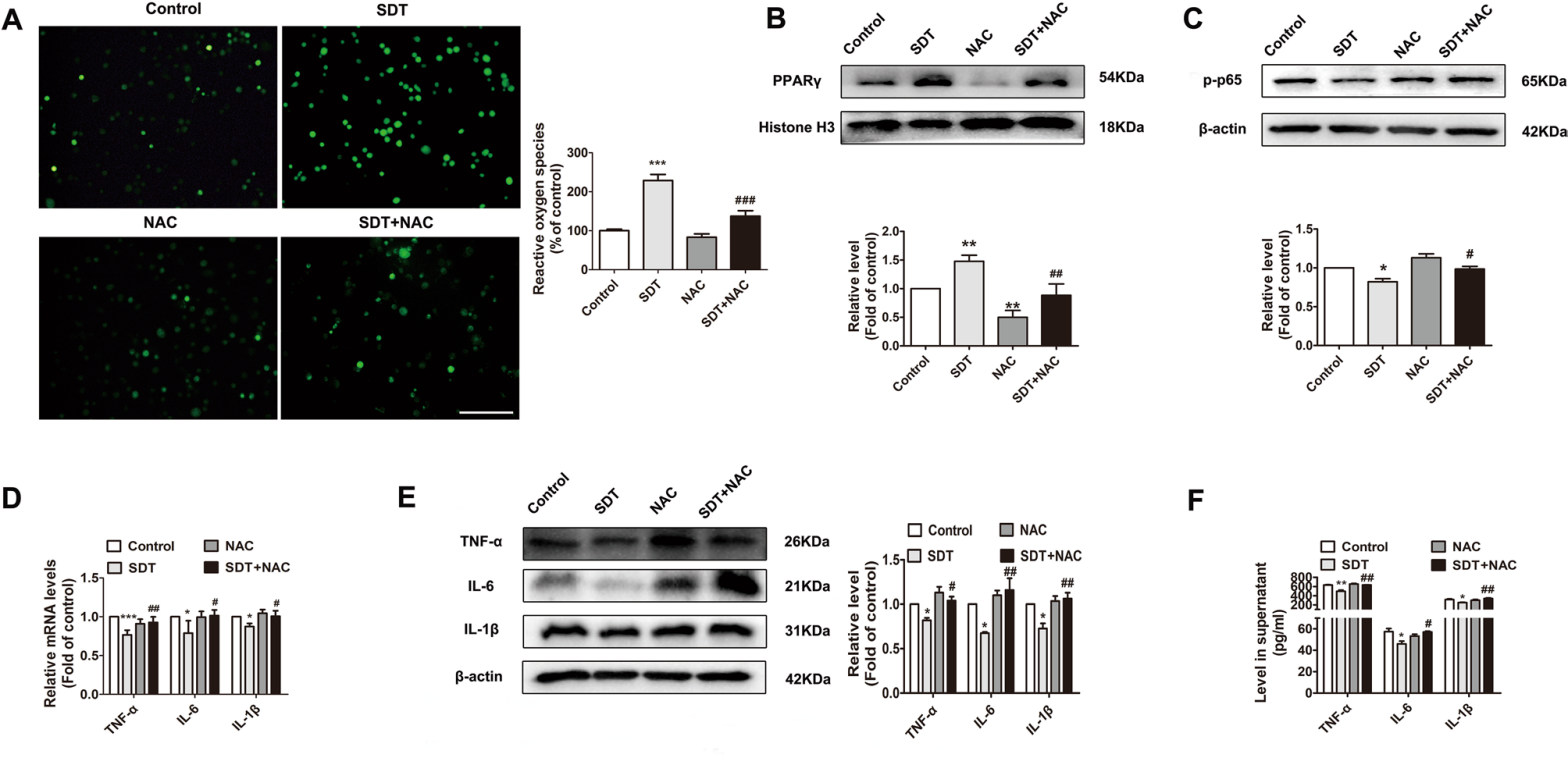
ALA-SDT stimulates ROS production and regulates PPARγ-NF-κB signalling in THP-1-derived macrophages. The cells were pre-treated with NAC for 1 h and loaded with DCFH-DA for 30 min, followed by ALA-SDT treatment. Immediately after treatment, the fluorescence of DCF was detected by fluorescence microscopy and a microplate reader (**A**) (n = 7). Scale bar = 200 μm. (**B**) Western blot analysis of the nuclear protein level of PPARγ. (**C**) Western blot analysis of the p-p65 level. (**D**) Quantitative real-time polymerase chain reaction analysis of mRNA levels of TNF-α, IL-6 and IL-1β. (**E**) Western blot analysis of TNF-α, IL-6 and IL-1β expression. (**F**) ELISA analysis of TNF-α, IL-6 and IL-1β in supernatants (n = 5). NAC, *N*-acetyl-l-cysteine.*p < 0.05, **p < 0.01 and ***p < 0.001 versus control. ^#^*p* < 0.05, ^##^*p* < 0.01 and ^###^*p* < 0.001 versus SDT.

Our previous studies have shown that biological effects caused by ALA-SDT are dependent on ROS generation in mitochondria^20,21^. Here, we proved that ALA-SDT-induced ROS production (Fig. 8A), PPARγ activation (Fig. 8B), and NF-κB p65 phosphorylation inhibition (Fig. 8C) and that the reductions in TNF-α, IL-6 and IL-1β (Fig. 8D–F) were reversed by the ROS scavenger *N*-acetyl-l-cysteine (NAC).

## Discussion

In this study, we performed early intervention of ALA-SDT at day 3 after balloon denudation in hypercholesterolemic rabbits since the maximum macrophage infiltration was reached at that time point. Previously, we demonstrated that macrophages represent the main target cell type for ALA-SDT in atherosclerotic plaques^12,22^. Considering the pivotal role of macrophage-mediated inflammation in neointima formation^8^, ALA-SDT may exert protective effects by reducing macrophages from the early stage. Interestingly, fluorescence spectra analysis showed that ALA-PpIX peaks at 1 h in the injured femoral artery, which differs from our previous result that ALA-PpIX peaks at 2 h in established plaque^23^. Indeed, we found that most macrophages infiltrate into freshly injured arterial subintima and reside inside the media of pre-existing plaques. Thus, it would take less time for macrophages in freshly injured vessels to phagocytise ALA.

As early as day 1 after ALA-SDT treatment, the rate of apoptotic macrophages had already increased, while TNF-α, IL-6 and IL-1β in the media of the femoral artery decreased significantly. At day 28 after ALA-SDT treatment, OCT, FFR and histopathological analysis showed significantly inhibited intimal hyperplasia with fewer macrophages and proliferative SMCs and reduced collagen content but increased elastin content without affecting endothelium regeneration. It is well known that infiltrated macrophages in the subintimal space produce pro-inflammatory cytokines, enzymes and chemokines to promote the proliferation and migration of SMCs. After dedifferentiation from the contractile to synthetic phenotype, SMCs migrate into the subendothelial space to excessively proliferate, and a large amount of collagen is secreted, accompanied by a decrease in elastin expression, leading to intima hyperplasia and stenosis of the lumen^7,24,25^. Thus, ALA-SDT induces depletion of activated macrophages and reduction in pro-inflammatory cytokines, which may promote phenotypic switching of SMCs, thereby inhibiting intimal hyperplasia.

In the present study, ALA-SDT parameters that induce THP-1-derived macrophage apoptosis had no direct effect on cytokine secretion in HUASMCs. However, after co-culturing ALA-SDT-treated macrophages with HUASMCs, the proliferation, migration and IL-6 production of HUASMCs were significantly inhibited. We previously found that after incubating 1 mM ALA with THP-1-derived macrophages and SMCs separately for 3 h, the fluorescence intensity of ALA-PpIX increased by 12-fold in THP-1-derived macrophages and by twofold in SMCs^20,26^. Additionally, after incubation of 1 mM ALA with SMCs for 24 h, ALA-SDT inhibited the proliferation and migration of SMCs and enhanced the expression of differentiated phenotypic markersin SMCs^26^. These results suggest that ALA-SDT had no direct effect on HUASMCs after incubation with ALA for 3 h, as HUASMCs uptake sono-sensitizers much slower than macrophages. Since inflammatory cytokines are widely recognized to activate SMCs^27–29^, we inferred that ALA-SDT-induced TNF-α, IL-6 and IL-1β reduction in macrophages indirectly inhibited HUASMC proliferation, migration, and IL-6 expression (Supplementary Fig. S9).

In the current study, we observed the effects of ALA-SDT on macrophages via NF-κB signalling inhibition, which was further confirmed by the transcription factor profiling array. ALA-SDT inhibited the transcriptional activity of NF-κB but increased the transcriptional activity of PPARγ. Hence, inhibition of PPARγ reversed the effects of ALA-SDT effect on NF-κB activity. We previously proved that 1.16 W/cm^2^ ultrasound intensity-mediated SDT produces a large amount of ROS to induce macrophage necrosis, while low ultrasound intensity-mediated SDT (0.48–0.84 W/cm^2^) produces a small amount of ROS to induce macrophage apoptosis^30^. Unlike other studies showing that ROS directly activate NF-κB signalling, which in turn aggravates the inflammatory response^31^, our results demonstrated that small amounts of ROS produced by low intensity ALA-SDT inhibit NF-κB signalling by activating PPARγ.

There are three limitations in this study. Although we confirmed that ALA-SDT inhibits intimal hyperplasia without affecting re-endothelialization in balloon-injured hypercholesterolemic rabbits, whether ALA-SDT can effectively exert the same effect upon BMS implantation requires further investigation. Moreover, the underlying cellular interaction between ALA-SDT-treated macrophages and SMCs needs further clarification. Finally, how ALA-SDT-induced ROS production activated PPARγ and subsequently inhibited NF-κB remains to be elucidated.

In summary, early intervention with ALA-SDT efficiently inhibits intimal hyperplasia without affecting re-endothelialization; hence, its clinical application combined with BMS implantation represents a potential strategy for decreasing bleeding risk caused by prolonged dual-antiplatelet regimen after DES deployment.

## Methods

### Reagents

5-Aminolevulinic acid (ALA) and phorbol-12-myristate-13-acetate (PMA) were purchased from Sigma-Aldrich (St Louis, MO, USA). DCFH-DA was purchased from Beyotime Biotechnology (Beijing, China). GW9662 (1 mM) and *N*-acetyl-l-cysteine (NAC, 1 mM) from Santa Cruz Biotechnology (Santa Cruz, CA, USA) were added to THP-1-derived macrophages 1 h before sonication. Recombinant human TNF-α protein was purchased from Abcam (Cambridge, UK). For Western blots and immunofluorescence analysis, horseradish peroxidase-conjugated or FITC-conjugated secondary antibodies were purchased from ZSGB-BIO (Beijing, China). 4′6-diamidino-2-phenylindole (DAPI) was purchased from Abcam. All primary antibodies are listed in Supplementary Table 1.

### Rabbit model and groups

Animal protocols were approved by the Ethics Committee for Animal Experiments of Harbin Medical University. The methods were carried out in accordance with the Guide for the Care and Use of Laboratory Animals published by the US National Institutes of Health (8th edition, 2011). Male New Zealand White rabbits (2.5–3.0 kg) were fed a 2% cholesterol-containing diet (Solarbio Bioscience & Technology Co. Ltd., Shanghai, China) for one week before surgery. Then, the rabbits were intravenously anaesthetized with pentobarbital (30 mg/kg) and ketamine (10 mg/kg). A 6F sheath was inserted into the left common carotid artery after intravenous administration of heparin (100 IU/kg). A 2.75 × 14 mm balloon dilation catheter (JW Medical Systems, Shandong, China) was guided through the descending aorta into the right superficial femoral artery with guidewire assistance (Cordis, MiamiLakes, FL, USA), inflated to 6–8 atmospheres and withdrawn to the femoral bifurcation with a rotating motion three times to induce effective endothelial denudation. After the injury, the rabbits continued to receive the high cholesterol diet until being killed.

To examine macrophage infiltration in the balloon-denuded arteries, five healthy rabbits were used as uninjured controls. The injured rabbits were euthanized at 12, 24, 48 and 72 h (n = 5) after balloon denudation. To investigate the effects of ALA-SDT on neointima formation, 56 balloon-denuded rabbits were randomly assigned to the control, ultrasound, ALA and ALA-SDT groups (n = 14). Each group was divided into 2 subgroups (n = 7), and the rabbits were killed at day 1 and day 28 after ALA-SDT treatment.

### Cell culture

Cells from the human THP-1 monocyte cell line were purchased from the American Type Culture Collection (ATCC, Manassas, VA, USA) and cultured as previously described^20^. The cells were seeded into 35-mm Petri dishes (1.0 × 10^5^ cells/ml) and treated with 100 ng/ml PMA for 72 h for differentiation into macrophages. Human umbilical artery SMCs (HUASMCs) (BeNa Culture Collection, Beijing, China), which have been previously used as a cell model for intimal hyperplasia^32^, were cultured in DMEM (Gibco, Life Technologies) with 10% foetal bovine serum and activated with TNF-α (10 ng/ml) for 24 h before experiments.

### SDT procedure

For the rabbit experiments, ALA (60 mg/kg) was bolus injected via the ear vein 1 h before ultrasound exposure. The ultrasonic procedure was consistent with a previous study^12^. For THP-1-derived macrophages and TNF-α-stimulated HUASMCs, the parameters of ALA-SDT (1 mM ALA, 0.5 W/cm^2^, 5 min ultrasound exposure time, 10% duty cycle) were the same as previously described^20^.

### Plasma cholesterol measurement

Rabbit blood was collected at day 0 (baseline), day 7 (balloon denudation time), day 11 (first harvest time) and day 38 (second harvest time). Plasma cholesterol was then measured by an automated analyser (AUC5800, Beckman Coulter, Inc., USA).

### ALA-PpIX fluorescence intensity detection

To delineate the time course of ALA-PpIX maximal accumulation in balloon-denuded arteries, the rabbits were sacrificed at 0, 30, 60, 90, 120 and 150 min after ALA injection (n = 3). The injured part of the femoral artery was removed, cut longitudinally to expose the intimal surface and washed thoroughly with phosphate buffered saline (PBS). Fluorescence emission spectra of PpIX were detected as described previously^23^. Emission spectra from 350 to 900 nm were collected every 3 mm across the entire area of the exposed intimal surface. Data were analysed by OriginPro7.0 software (OriginLab, Massachusetts, USA).

TNF-α-stimulated HUASMCs (2 × 10^5^ cells/mL) seeded in 96-well plates were incubated with 1 mM ALA for 0, 2, 4, 8, 12 and 24 h. Then, the cells were washed three times with PBS, and ALA-PpIX fluorescence intensity was measured at 405 nm excitation and 630 nm emission wavelengths using a multimode microplate reader (Tecan Infinite M200 Pro, Mannedorf, Switzerland).

### Tissue preparation

The rabbits were euthanized by pentobarbital overdose (100 mg/kg). The arterial tree was perfused at 100 mmHg and stored in 4% paraformaldehyde for 24 h, embedded in paraffin and dissected for morphology studies. Each block of the injured artery was divided into 3 parts: both ends and a middle part. Serial transverse sections (5 μm) were cut at each part for histopathology and immunohistochemistry analysis. For immunofluorescence staining and ALA-PpIX fluorescence detection, the arteries were embedded in optimum cutting temperature compound (Sakura Fine Technical, Tokyo, Japan) and snap-frozen in liquid nitrogen.

### Immunofluorescence staining

Frozen sections (7 μm) of rabbit arteries or HUASMCs were fixed, permeabilized and blocked, followed by incubation with primary antibody at 4 °C overnight. Rinsed sections or cells were then incubated with FITC-conjugated secondary antibody for 1 h at room temperature in the dark and with DAPI for another 2 min. The sections or cells were washed twice with PBS and analysed with a fluorescence microscope.

### Colocalization of ALA-PpIX fluorescence and macrophages

One hour after ALA injection, the balloon-denuded arteries were snap-frozen and cryosectioned immediately for the fluorescence detection of PpIX. To detect the cellular localization of PpIX, the same cryosection was processed for immunofluorescence staining of macrophages, and colocalization was examined with a fluorescence microscope.

### Histopathology and immunohistochemistry

Paraffin sections were stained with haematoxylin and eosin (HE) to identify the demarcations of the internal elastic lamina (IEL), the external elastic lamina (EEL) and the lumen. Masson, Weigert and Oil Red O staining were performed to determine the content of collagen, elastin and lipids. TUNEL staining (In situ cell death detection kit, Roche, Mannheim, Germany) was performed to detect apoptosis according to the manufacturer’s instructions.

For sections stained by immunohistochemistry, images were recorded with an Olympus IX70 microscope (Olympus, Tokyo, Japan) and analysed by Image Pro-plus 6.0 (Media Cybernetics, Rockville, MD, USA). The results of the stained area were expressed as percent positive area over the vascular media area or neointima. The number of macrophages and SMCs and the percentage ratio of macrophages and SMCs to the total cell number in neointima were semi-quantified. Re-endothelialization was scored for each cross-section by the extent of the circumference of the arterial lumen covered by endothelium: 1 = 25%; 2 = 25%–75%; and 3 = 75%–100% coverage, and data were expressed as averages of three independent scores.

### Angiography

The angiography procedure was performed as previously described^33^.

### Optical coherence tomography (OCT) imaging

Seven normal healthy rabbits were euthanized as a baseline control. The OCT imaging procedure was performed as previously described^12^.

### Fractional flow reserve (FFR)

The FFR measurement procedure was performed as previously described^12^.

### Western blots

The balloon-denuded arteries harvested at day 1 after ALA-SDT were cut into pieces (n = 3) and lysed in RIPA buffer containing PMSF on ice. THP-1-derived macrophages harvested at 6 h after ALA-SDT and HUASMCs harvested at 24 h after co-culturing with ALA-SDT-treated THP-1-derived macrophages were lysed in RIPA buffer containing PMSF on ice. The nuclear and cytosolic fractions were obtained by using a nuclear/cytosol fractionation kit (Beyotime). After quantification and denaturation, the proteins were electrophoresed in SDS–polyacrylamide gels and transferred onto PVDF membranes (Millipore, Schwalbach, Germany). The membranes were incubated with primary antibodies. Antibody labelling was detected using a ChemiDoc™ MP Imaging System (Bio-Rad, CA, USA) and quantified using Quantity One software analysis (Bio-Rad, USA). Data were collected from at least three independent experiments.

### Quantitative real-time PCR

Total RNA was extracted using TRIzol reagent (Invitrogen, Carlsbad, California, USA) and reverse-transcribed using ReverTra Ace qPCR RT Master Mix with gDNA Remover (TOYOBO, Osaka, Japan). Then, 1 μl of cDNA was amplified in SYBR Green Real-time PCR Master Mix (TOYOBO) on an ABI 7500 Fast Sequence Detection System (Applied Biosystems, CA, USA). Gene expression values were normalized to β-actin. mRNA was quantified by the 2^−△△Ct^ method. Primers are listed in Supplementary Table 2.

### Macrophage and HUASMC co-culture

THP-1-derived macrophages were divided into control and SDT groups. Six hours after ALA-SDT, THP-1-derived macrophages were harvested, transferred to the upper chamber of co-culture plates (Corning, NY, USA) at a concentration of 6 × 10^5^ cells/ml and co-cultured with TNF-α-stimulated HUASMCs (3 × 10^5^ cells/ml) in the lower chamber for 24 or 48 h using DMEM.

### HUASMC proliferation assay

Ki67 immunofluorescence staining was used to detect proliferating HUASMCs after co-culture for 48 h with THP-1-derived macrophages treated with ALA-SDT. The percentage of Ki67-positive cells was calculated from total cells. At least 1,000 total cells from five random microscopic fields for each well at 20× magnification were counted.

### HUASMC migration assay

TNF-α-stimulated HUASMCs were wounded by manual scraping with a 200 μl pipette tip and washed. The HUASMCs were then co-cultured with THP-1-derived macrophages treated with ALA-SDT using serum-free DMEM, and their migration distance was recorded at 0, 12 and 24 h from the beginning of co-culture. Cell migration was photographed at identical locations for each time point. Phase-contrast images were recorded using a microscope (Olympus, Tokyo, Japan). The width of the wound in three random fields at 4× magnification in each well was analysed by Image Pro-plus 6.0 at each time point to determine the healed distance.

### Enzyme linked immunosorbent assay (ELISA)

The supernatants from THP-1-derived macrophages and TNF-α-stimulated HUASMCs were collected at 4 h after ALA-SDT, and the supernatants from the co-culture system were collected at 24 h after co-culture. TNF-α, IL-6 and IL-1β were measured using ELISA kits (Elabscience Biotechnology Co., Ltd., Wuhan, China) according to the manufacturer’s instructions.

### Flow cytometry analysis

Flow cytometry was performed as previously described^11^. Briefly, 6 h following ALA-SDT in the absence or presence of GW9662, THP-1-derived macrophages were collected and analysed by a fluorescence-activated cell sorting system within 1 h. Annexin V^+^/PI^−^ and Annexin V^+^/PI^+^ represent the apoptotic cells in the early and late phases, respectively. The data were analysed using BD FACSDiva Software 7.0 (Becton–Dickinson, USA).

### Reactive oxygen species determination

Intracellular ROS generation was determined using DCFH-DA (Beyotime Biotechnology, Beijing, China) as described previously^20^. Briefly, seeded THP-1-derived macrophages (2 × 10^5^ cells/ml) were pre-treated with NAC for 1 h and loaded with DCFH-DA for 30 min, followed by ALA-SDT treatment. Immediately after ALA-SDT treatment, the cells were analysed using a multimode microplate reader at 488 nm excitation and 525 nm emission wavelengths. Then, the cells were photographed under a fluorescence microscope (Olympus Corporation, Tokyo, Japan).

### Transfection

Knockdown of PPARγ in THP-1-derived macrophages was obtained by siRNA as previously described^34^. A scrambled 21-nucleotide siRNA was used as a negative control (GenePharma, Shanghai, China). The target sequences were as follows: PPARγ siRNA: 5′-GGUUGCAGAUUACAAGUAUTT-3′; Scrambled siRNA: 5′-UUCUCCGAACGUGUCACGUTT-3′. The efficiency of transfection was confirmed using Western blot analysis (Supplementary Fig. S7).

### Confocal laser scanning

Confocal imaging was performed as previously described^11^. Briefly, six hours after ALA-SDT, THP-1-derived macrophages seeded in glass bottom cell culture dishes were stained with p65 and examined with a confocal laser scanning microscope (Zeiss, Germany).

### Transcription factor profiling array

The activity of 96 different transcription factors in THP-1-derived macrophages was analysed by a Transcription Factor Activation Profiling Plate Array II (Signosis, Sunnyvale, CA, USA). Nuclear components were isolated using a Nuclear Extraction Kit (Signosis). In brief, both the control group and the SDT group contained ten samples from 35-mm Petri dishes of THP-1-derived macrophages. In each group, the nuclear components were extracted 4 h after treatment from pooled 10 dish samples. Then, biotin-labelled probes were mixed with 15 μg of nuclear protein extract to form transcription factor/probe complexes. After separation, elution and hybridization, the captured DNA probe was detected with streptavidin–horseradish peroxidase (HRP), and signal intensity was measured with a microplateluminometer (Luminoskan Ascent, Thermo Scientific). Data were collected from three independent experiments.

### Statistical analysis

All quantitative data are expressed as the mean ± SEM. The statistical analysis was performed by GraphPad 6.0 (La Jolla, CA, USA). The normality test (Shapiro–Wilk) was performed to determine whether the data were normally distributed. Student’s unpaired test was used to determine the significant differences between two groups. One-way analysis of variance (ANOVA) with repeated measures and ANOVA followed by Tukey's test were used to determine the significance of differences among the groups in plasma cholesterol values and the other data, respectively. Two-way ANOVA with repeated measures followed by Dunnett's test was used to determine the significance of differences among the groups in HUASMC migration. A *p* value < 0.05 was considered to be statistically significant.

## Supplementary information

Supplementary information.

## Data Availability

The datasets generated during and/or analysed during the current study are available from the corresponding author on reasonable request.
