# Supplementary material for: Early modulation of macrophage ROS-PPARγ-NF-κB signalling by sonodynamic therapy attenuates neointimal hyperplasia in rabbits
Source: Sci Rep. 2020 Jul 15;10:11638. doi: 10.1038/s41598-020-68543-9 (PMC7363872; doi:10.1038/s41598-020-68543-9)
Supplement: Supplementary file 1 — Supplementary information. [file 41598_2020_68543_MOESM1_ESM.docx]

**Early Modulation of Macrophage ROS-PPARγ-NF-κB Signalling by Sonodynamic Therapy Attenuates Neointimal Hyperplasia in Rabbits**

Jianting Yao^1*^; Xuezhu Zhao^1*^; Fancheng Tan^2*^; Xiaoru Cao^2^; Shuyuan Guo^1^; Xiang Li^2^; Zhen Huang^1^; Kamal Diabakte^1^; Lu Wang^1^; Mingyu Liu^1^; Zhaoqian Shen^1^; Bicheng Li^1^; Zhengyu Cao^1^; Siqi Sheng^1^; Minqiao Lu^2^; Yang Cao^1^; Hong Jin^3^; Zhiguo Zhang^4^; Ye Tian^1,2,5^^†^

^1^Department of Cardiology, the First Affiliated Hospital, Cardiovascular Institute, Harbin Medical University, Harbin, China; ^2^Department of Pathophysiology and Key Laboratory of Cardiovascular Pathophysiology, Harbin Medical University, Key Laboratory of Cardiovascular Medicine Research (Harbin Medical University), Ministry of Education, Harbin, China; ^3^Karolinska Institute, Department of Medicine, Stockholm, Sweden; ^4^Laboratory of Photo- and Sono-theranostic Technologies and Condensed Matter Science and Technology Institute, Harbin Institute of Technology, Harbin, China; ^5^Heilongjiang Academy of Medical Sciences, Harbin, China.

*Jianting Yao, Xuezhu Zhao and Fancheng Tan contributed equally to this study.

^†^**Corresponding Author**

Ye Tian, MD, PhD

Department of Cardiology, the First Affiliated Hospital, Cardiovascular Institute, Harbin Medical University, 23 Youzheng Street, Harbin 150001, China

Tel.: +86 451 85555943,

Fax: +86 451 87530341

E-mail: [yetian@ems.hrbmu.edu.cn](mailto:yetian@ems.hrbmu.edu.cn)

**Supplementary Fig.S1**

**
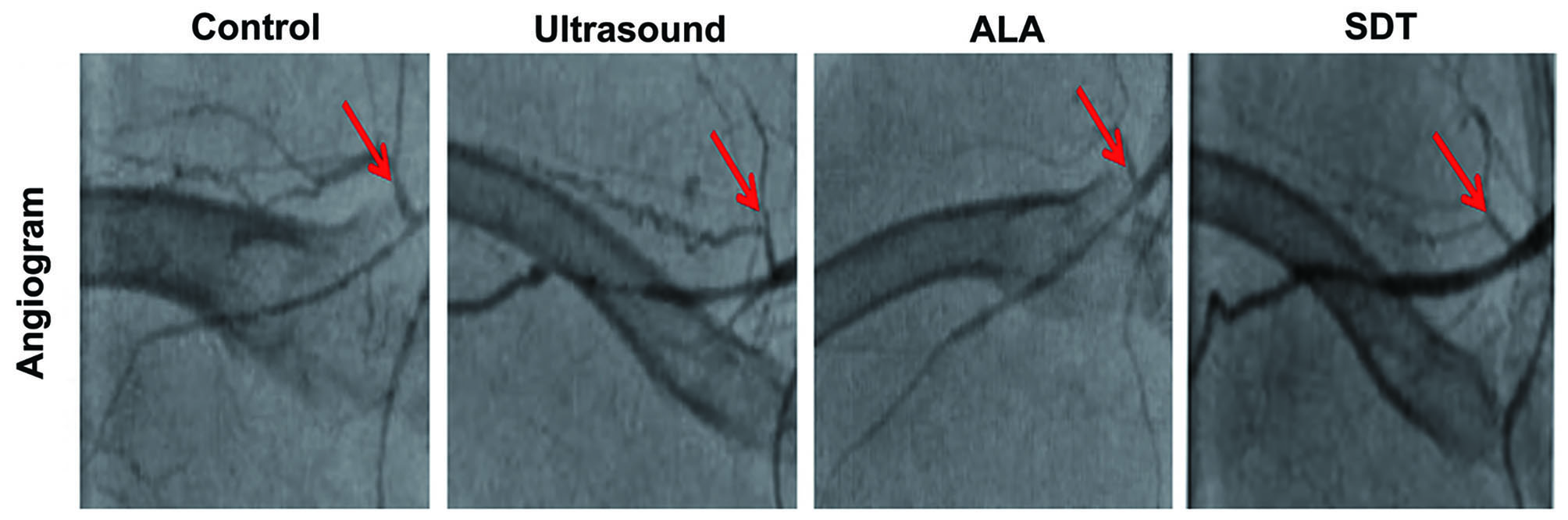
**

**Supplementary Fig. S1** Angiogram showing the increase in lumen diameter in a balloon-denuded right femoral artery at day 28 after ALA-SDT treatment. Red arrows indicate the femoral bifurcation.

**Supplementary Fig.S2**

**
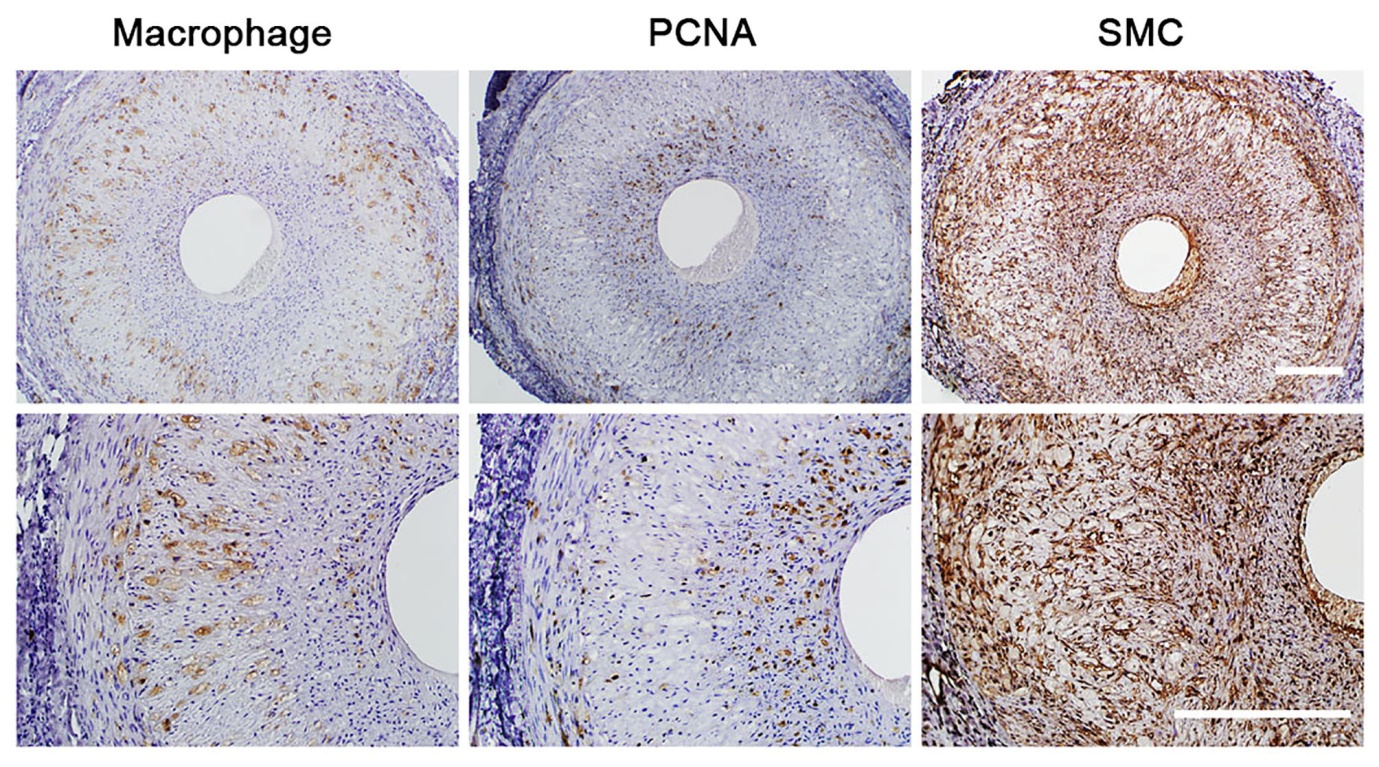
**

**Supplementary Fig. S2** Immunohistochemistry analysis of macrophages (RAM-11), PCNA and smooth muscle cells (α-SMA) in serial sections of balloon-denuded rabbit femoral arteries. PCNA, proliferating cell nuclear antigen. Scale bar = 500 μm.

**Supplementary Fig. S3**

**
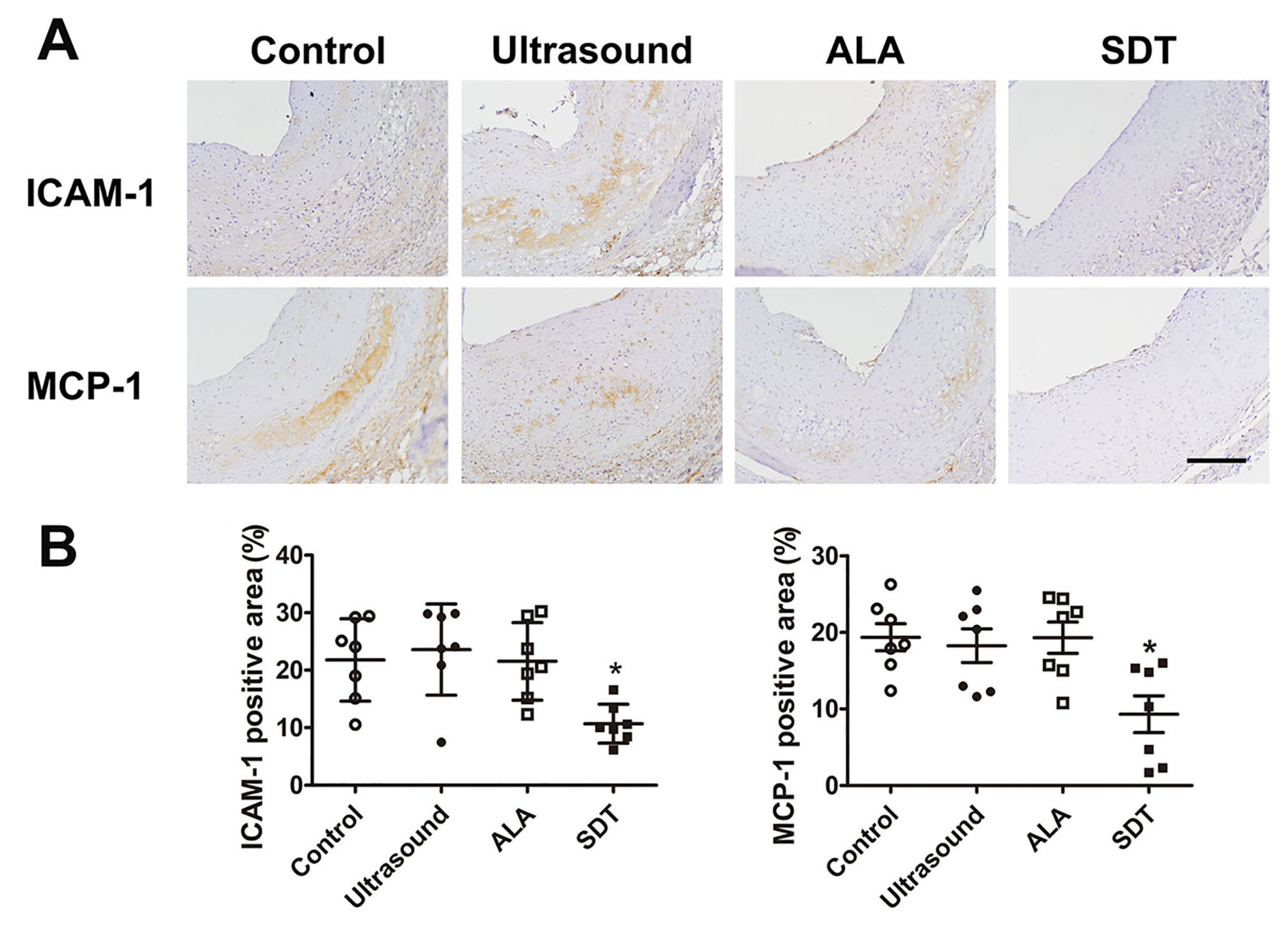
**

# Supplementary Fig. S3 Immunohistochemical analysis of intercellular adhesion molecule-1 (ICAM-1) and monocyte chemotactic protein-1 (MCP-1) expression in the neointima at day 28 after ALA-SDT treatment (n = 7). Scale bar = 250 μm. Each data point represents one rabbit. **p* < 0.05 versus control.

**Supplementary Fig.S4**

**
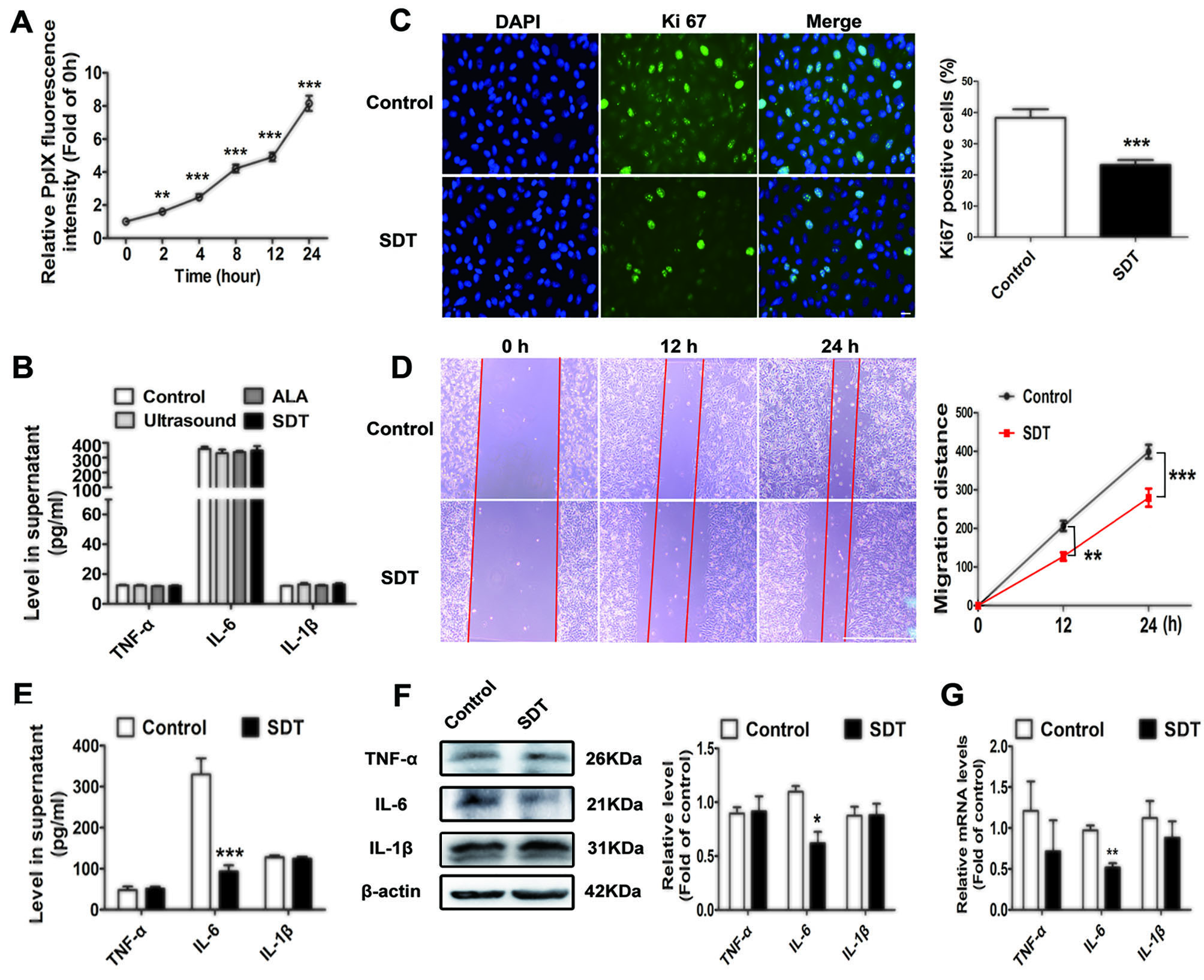
**

**Supplementary Fig. S4** ALA-SDT-treated THP-1-derived macrophages inhibit HUASMC proliferation, migration and IL-6 secretion. (**A**) Representative fluorescence intensity of ALA-PpIX in HUASMCs. TNF-α-stimulated HUASMCs were incubated with 1 mM ALA for 0 - 24 h, and the fluorescence intensity of ALA-PpIX in HUASMCs was measured by a microplate reader (n = 6). (**B**)ELISA analysis of TNF-α, IL-6 and IL-1β in the supernatant 4 h after ALA-SDT treatment (n = 4). TNF-α-stimulated HUASMCs were treated with ALA-SDT, and the supernatant was harvested. **(C)** Representative immunofluorescence staining and quantification of the proliferation of HUASMCs at 48 h after co-culture (n = 4). THP-1-derived macrophages were divided into control and SDT groups. Six hours after ALA-SDT, THP-1-derived macrophages were harvested, transferred to the upper chamber of co-culture plates and co-cultured with TNF-α-stimulated HUASMCs in the lower chamber using DMEM. Scale bar = 20 μm. **(D)** Representative photographs and migration distance of HUASMCs at 0, 12 and 24 h from the beginning of co-culture (n = 3). TNF-α-stimulated HUASMCs were wounded by manual scraping with a 200 μl pipette tip and washed. The HUASMCs were then co-cultured with THP-1-derived macrophages treated with ALA-SDT using serum-free DMEM. Scale bar = 2 mm. **(E)** ELISA analysis of TNF-α, IL-6 and IL-1β in the supernatant 24 h after co-culture(n = 4). **(F)** Western blot analysis of TNF-α, IL-6 and IL-1β expression in HUASMCs at 24 h after co-culture. **(G)** Quantitative real-time polymerase chain reaction analysis of the mRNA levels of TNF-α, IL-6 and IL-1β in HUASMCs at 24 h after co-culture. Data on the graph are representative of three independent experiments. HUASMCs, human umbilical artery smooth muscle cells. **p* < 0.05, ***p* < 0.01 and ****p* < 0.001 versus 0 h or control.

**Supplementary Fig.S5**

**
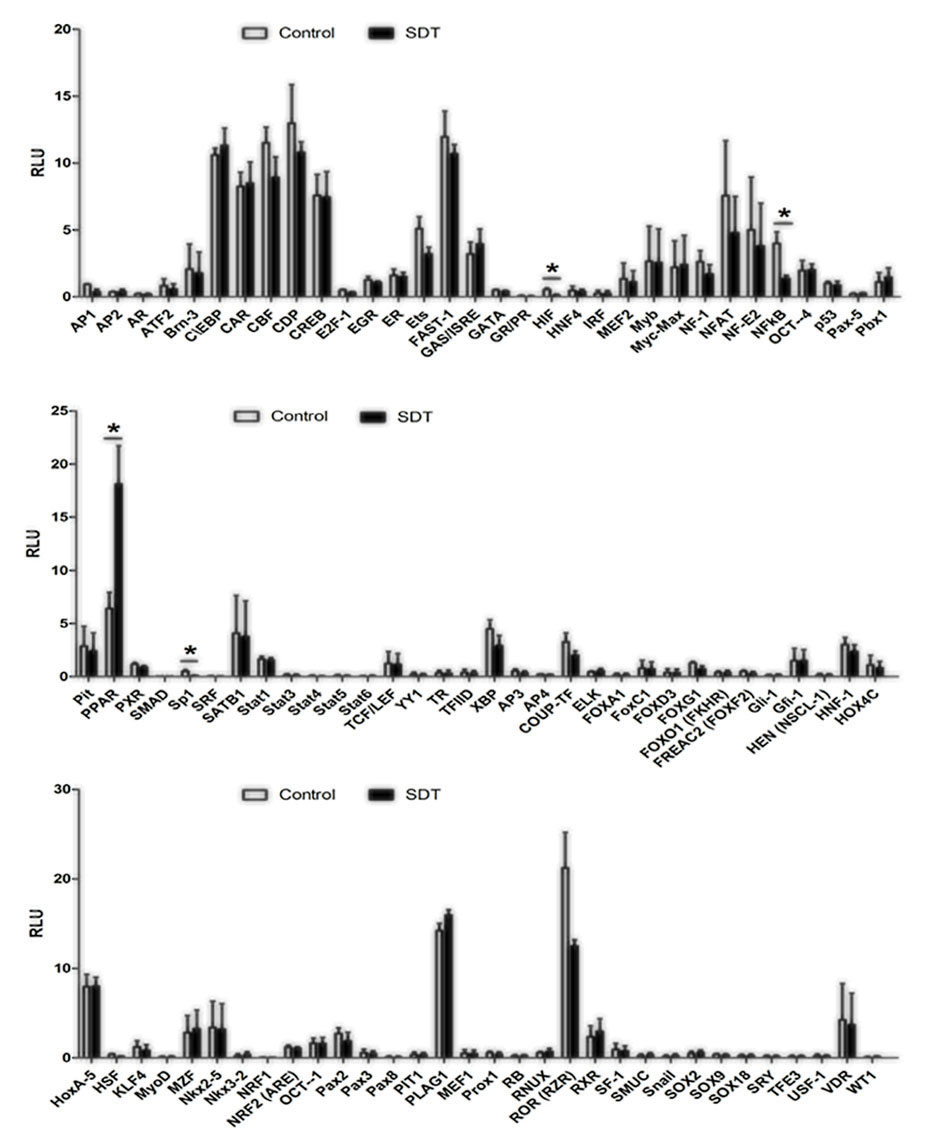
**

**Supplementary Fig. S5** Effect of ALA-SDT on 96 transcription factor activities in THP-1-derived macrophages. ALA-SDT upregulated PPAR and downregulated HIF, NF-κB and SP-1 DNA-binding activity compared with the control group. **p* < 0.05 versus control. RLU, relative light units.

**Supplementary Fig.S6**

**
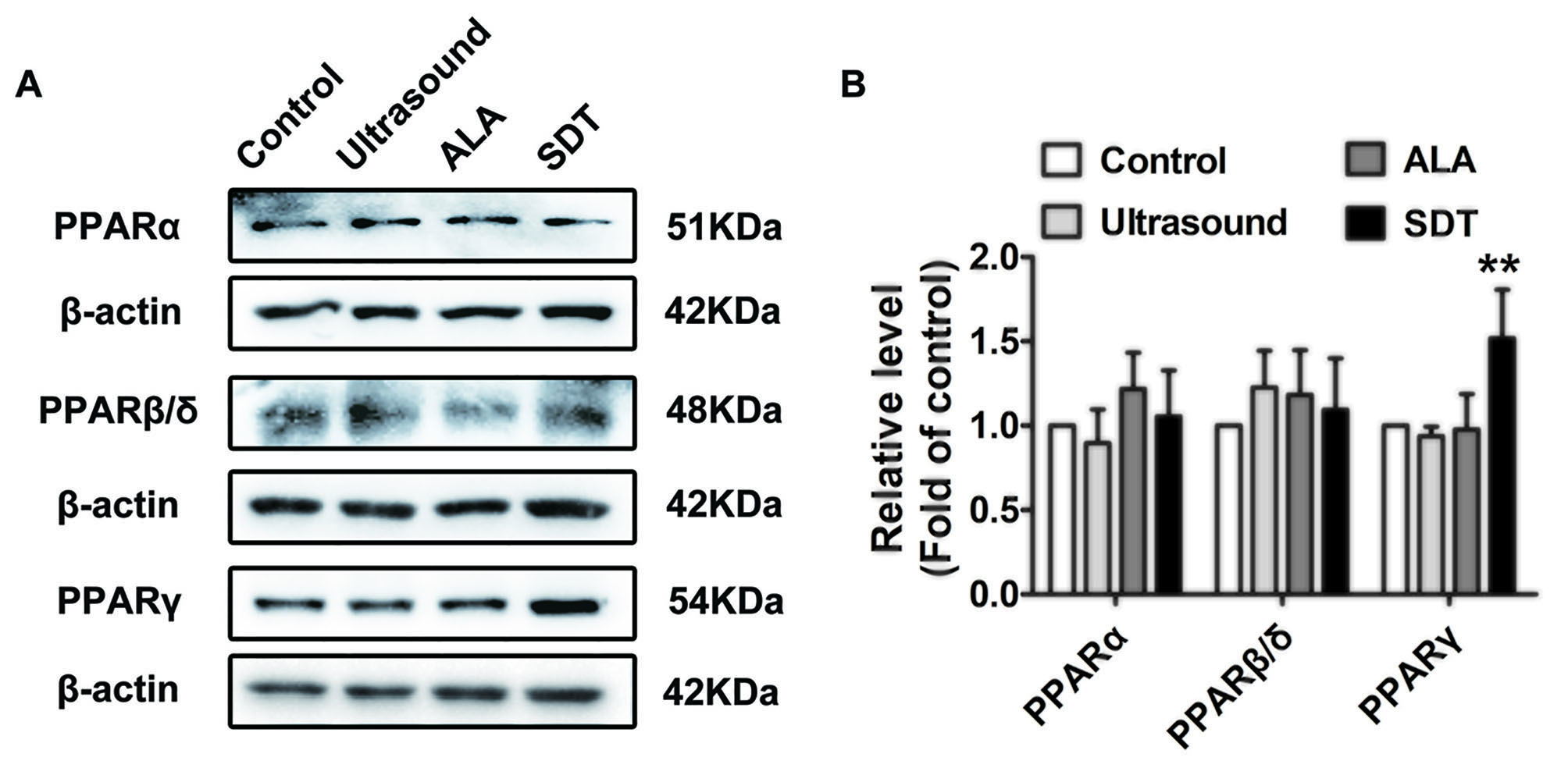
**

**Supplementary Fig. S6** Western blot analysis of the protein expression of PPARα, PPARβ/δ, and PPARγ in whole lysates of THP-1-derived macrophages. ***p* < 0.01 versus control.

**Supplementary Fig.S7**

**
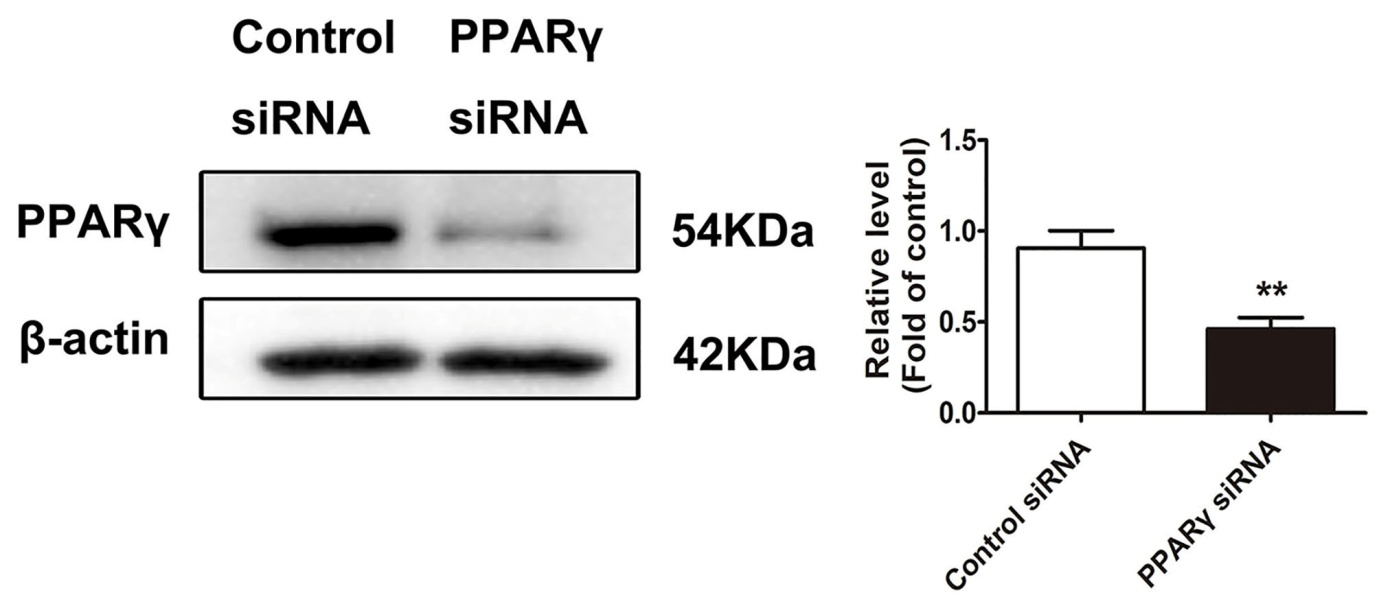
**

**Supplementary Fig. S7** Western blot analysis of PPARγ following siRNA treatment. ** *p* < 0.01 versus control siRNA.

**Supplementary Fig.S8**

**
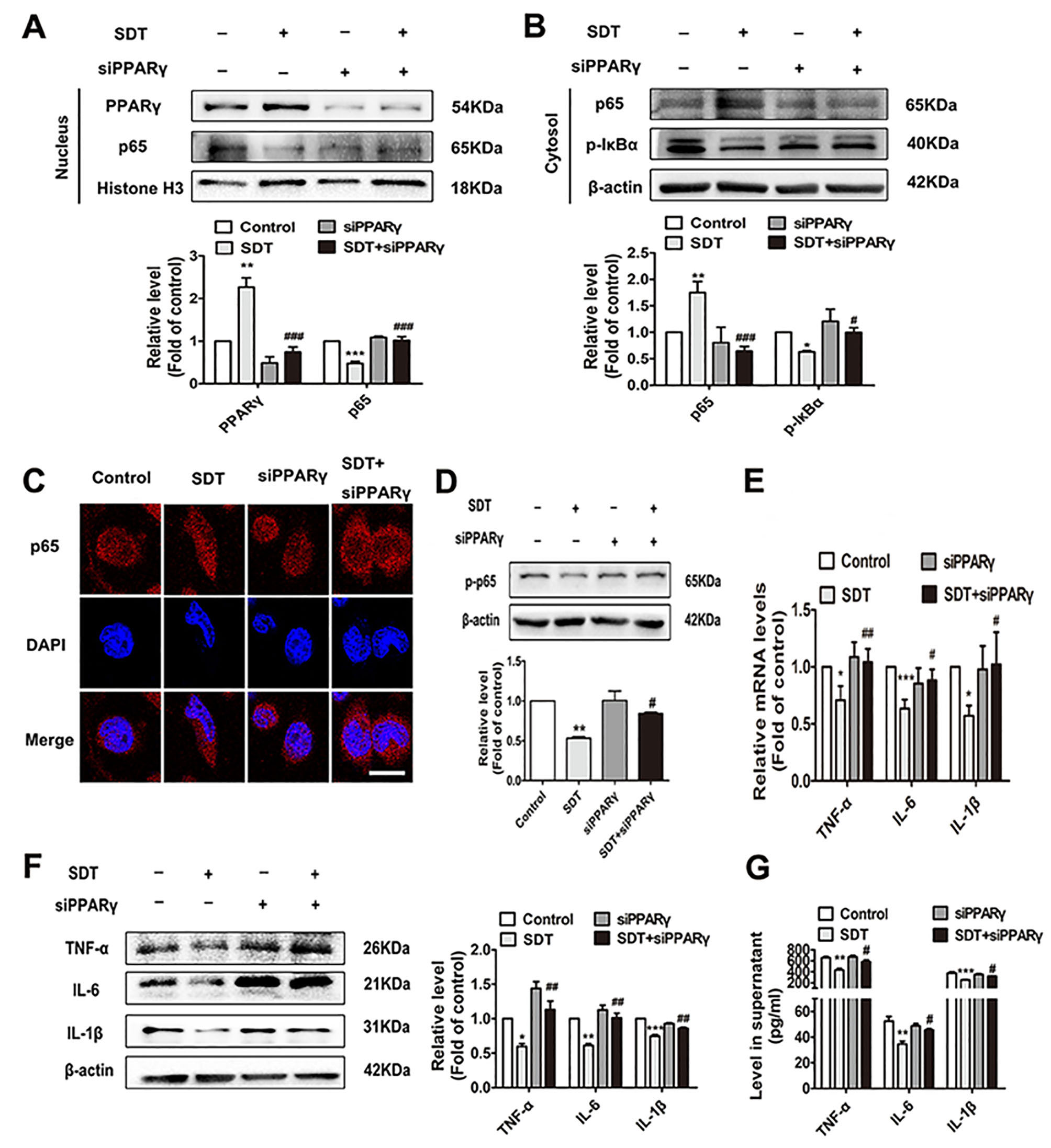
**

**Supplementary Fig. S8** ALA-SDT inhibits NF-κB signaling and reduces the expression and secretion of TNF-α, IL-6 and IL-1β by activating PPARγ in THP-1-derived macrophages. (**A**) Western blot analysis of the nuclear protein levels of PPARγ and NF-κB p65. (**B**) Western blot analysis of the cytoplasmic protein expression of NF-κB p65 and p-IκBα. (**C**) Immunofluorescence was evaluated to detect NF-κB p65 nuclear translocation. Scale bar = 20 μm. (**D**) Western blot analysis of the p-p65 level. (**E**) Quantitative real-time polymerase chain reaction analysis of mRNA levels of TNF-α, IL-6 and IL-1β. (**F**) Western blot analysis of TNF-α, IL-6 and IL-1β. (**G**) ELISA analysis of TNF-α, IL-6 and IL-1β in supernatants (n = 5). **p* < 0.05, ***p* < 0.01 and ****p* < 0.001 versus control. ^#^*p* < 0.05, ^##^*p* < 0.01 and ^###^*p* < 0.001 versus SDT.

**Supplementary Fig.S9**


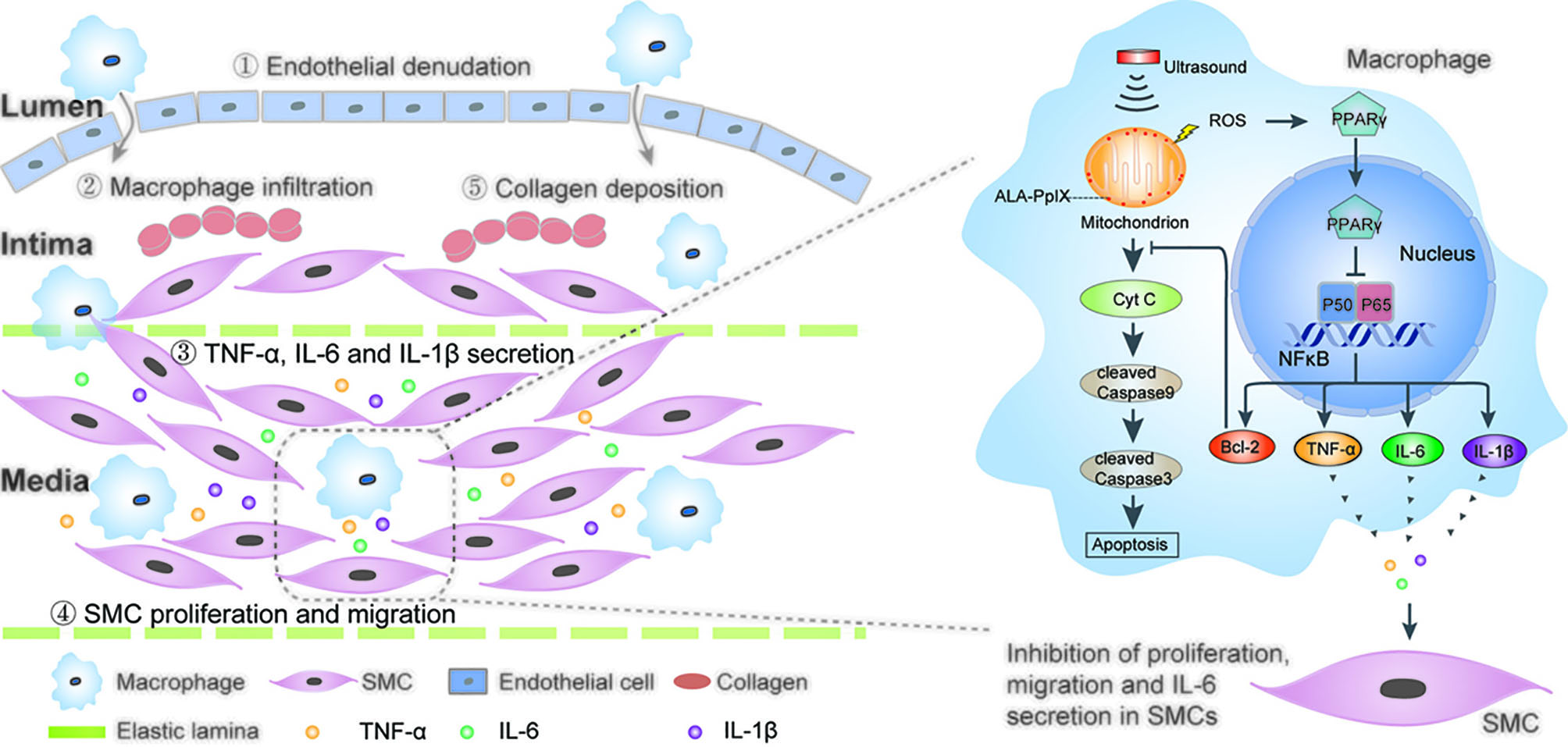


**Supplementary Fig. S9** Schematic illustration of the mechanism underlying the inhibitory effect of ALA-SDT on intimal hyperplasia. After endothelial denudation, infiltrated macrophages express proinflammatory cytokines (TNF-α, IL-6 and IL-1β) to initiate the proliferation and migration of smooth muscle cells (SMCs), leading to collagen deposition and intimal hyperplasia. ALA-SDT induces macrophage apoptosis and decreases the secretion of TNF-α, IL-6 and IL-1β via the ROS-PPARγ-NF-κB pathway. The cross-talk between SMCs and ALA-SDT-treated macrophages inhibits the proliferation, migration and IL-6 secretion in SMCs, resulting in the suppression of neointima formation.

**Supplementary Table 1. Primary antibodies used in this study**

|  | Antibody Name | Cat.Number | Application | Manufacturer Brand |
| --- | --- | --- | --- | --- |
| 1 | Rabbit anti-TNF-alpha | bs-2150R | WB=1:500 IHC-P=1: 400 | Bioss (Beijing, China) |
| 2 | IL-6 polyclonal antibody | 21865-1-AP | WB=1: 1000 | Proteintech (Wuhan, China) |
| 3 | Anti-IL1 beta antibody | ab9722 | WB=1: 2500 | Abcam (Cambridge, UK) |
| 4 | p65; RELA polyclonal antibody | 10745-1-AP | WB=1:1000 IF=1:100 | Proteintech (Wuhan, China) |
| 5 | Phospho-IκBα (Ser32) (14D4) rabbit mAb | #2859 | WB=1: 1000 | Cell Signaling Technology (Beverly, MA, USA) |
| 6 | Phospho-NF-kB p65 (Ser536) (93H1) rabbit mAb | #3033 | WB=1: 1000 | Cell Signaling Technology (Beverly, MA, USA) |
| 7 | Bcl-2 (124) mouse mAb | #15071 | WB=1: 1000 | Cell Signaling Technology (Beverly, MA, USA) |
| 8 | Cleaved caspase-9 (Asp330) (E5Z7N) rabbit mAb | #52873 | WB=1: 1000 | Cell Signaling Technology (Beverly, MA, USA) |
| 9 | caspase-9 p35 (A-9) | sc-133109 | WB=1: 100 | Santa Cruz Biotechnology (Santa Cruz, CA, USA) |
| 10 | Bax(2D2) | sc-20067 | WB=1: 100 | Santa Cruz Biotechnology (Santa Cruz, CA, USA) |
| 11 | Anti-caspase-3 antibody | ab4051 | WB=1: 500 | Abcam (Cambridge, UK) |
| 12 | Anti-active caspase-3 antibody | ab2302 | WB=1: 1000 | Abcam (Cambridge, UK) |
| 13 | Anti-BCL2L1 antibody produced in rabbit | AV30475 | WB=1: 1000 | Sigma-Aldrich (St Louis, MO, USA) |
| 14 | β-actin antibody | #4967 | WB=1: 1000 | Cell Signaling Technology (Beverly, MA, USA) |
| 15 | Anti-GAPDH antibody [GA1R] | ab125247 | WB=1: 1000 | Abcam (Cambridge, UK) |
| 16 | PPARγ(E-8) | sc-7273 | WB=1: 100 | Santa Cruz Biotechnology (Santa Cruz, CA, USA) |
| 17 | Rabbit anti-PPAR delta + beta polyclonal antibody | bs-0250R | WB=1: 500 | Bioss (Beijing, China) |
| 18 | PPARα polyclonal antibody | 15540-1-AP | WB=1: 1000 | Proteintech (Wuhan, China) |
| 19 | RAM-11 | M0633 | IHC-P=1: 1200 | Dako (Glostrup, Denmark) |
| 20 | Anti-α-SMA | A2547 | IHC-P=1: 2000 | Sigma-Aldrich (St Louis, MO, USA) |
| 21 | Anti-PCNA antibody [PC10] | ab912 | prediluted | Abcam (Cambridge, UK) |
| 22 | Anti-CD31 antibody [JC/70A] | ab9498 | IHC-P=1: 20 | Abcam (Cambridge, UK) |
| 23 | Rabbit anti-IL-6 | bs-6312R | IHC-P=1: 400 | Bioss (Beijing, China) |
| 24 | Rabbit Anti-IL-1 beta antibody | bs-0812R | IHC-P=1: 400 | Bioss (Beijing, China) |
| 25 | Rabbit anti-ICAM1 polyclonal antibody | bs-6326R | IHC-P=1: 400 | Bioss (Beijing, China) |
| 26 | Rabbit anti-MCP1 polyclonal antibody | bs-1955R | IHC-P=1: 400 | Bioss (Beijing, China) |
| 27 | Anti-Ki67 antibody | ab16667 | IF=1:250 | Abcam (Cambridge, UK) |
| 28 | Rabbit anti-collagen I antibody | bs-0578R | IHC-P=1: 200 | Bioss(Beijing, China) |
| 29 | Rabbit anti-collagen III antibody | bs-0549R | IHC-P=1: 200 | Bioss (Beijing, China) |

WB=western blots; IHC-P=immunohistochemistry for paraffin sections; IF=immunofluorescence.

**Supplementary Table 2. Sequences of qRT-PCR primers (5’-3’) used in this study**

| Human TNF-α | Forward | AGGACACCATGAGCACTGAA |
| --- | --- | --- |
|  | Reverse | CCGATCACTCCAAAGTGCAG |
| Human IL-6 | Forward | TGCGCAGCTTTAAGGAGTTC |
|  | Reverse | CCCATGCTACATTTGCCGAA |
| Human IL-1β | Forward | CTCTCTCCTTTCAGGGCCAA |
|  | Reverse | GCGGTTGCTCATCAGAATGT |
| Human β-actin | Forward | ACTCTTCCAGCCTTCCTTCC |
|  | Reverse | CAATGCCAGGGTACATGGTG |

**Uncropped blots**

**Figure 2A**

**
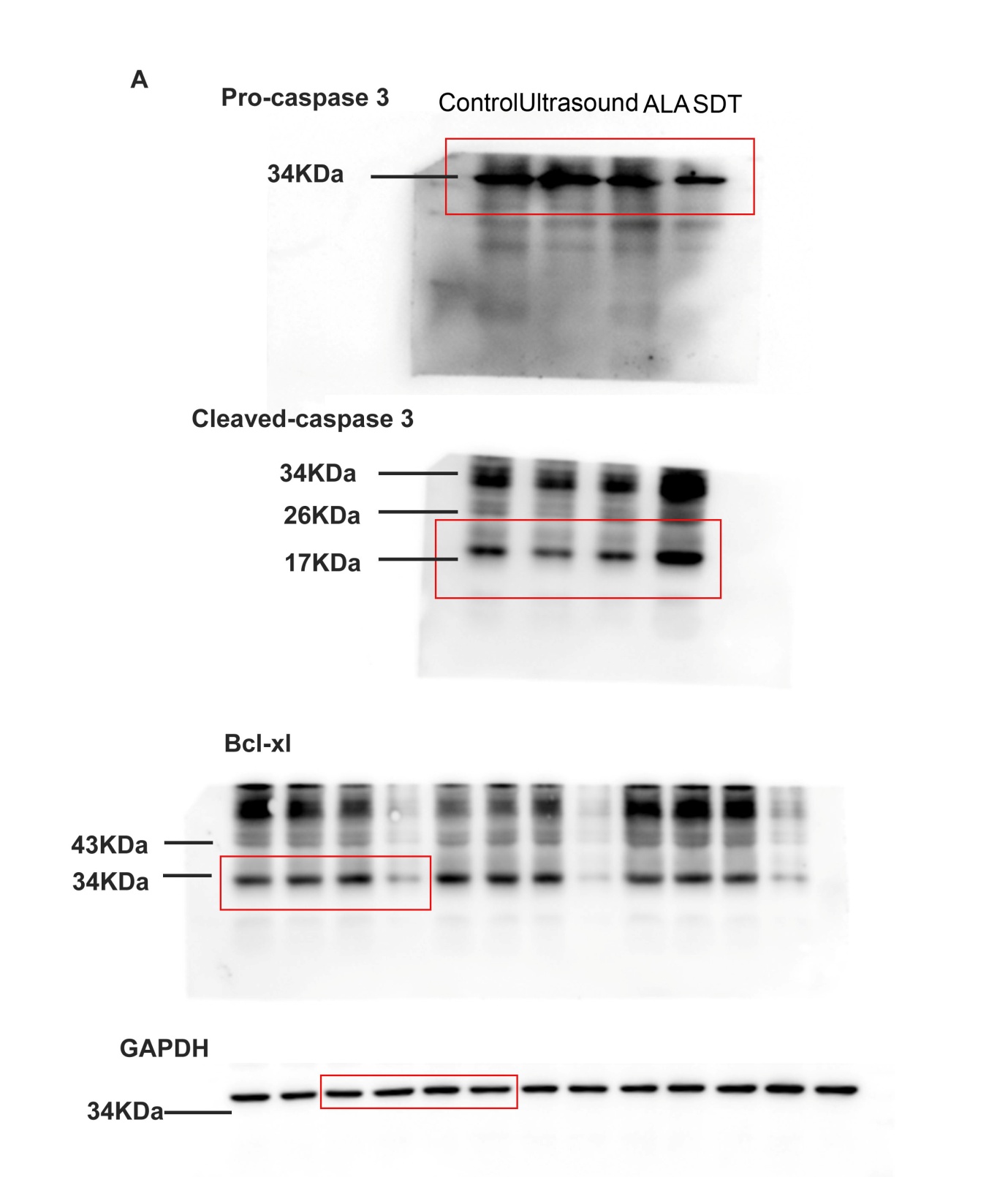
**

**Figure 5**

**
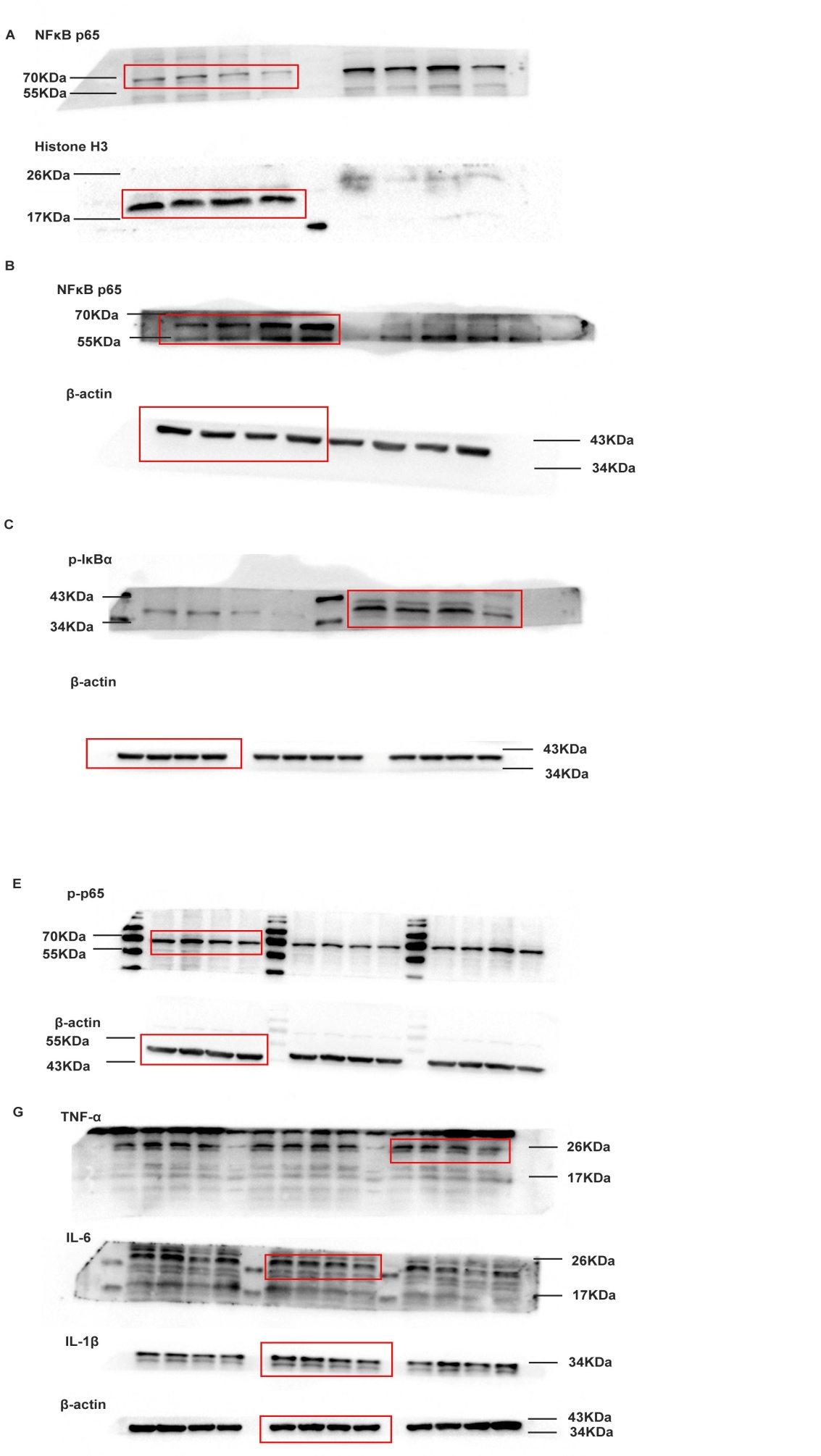
**

**Figure 6**

**
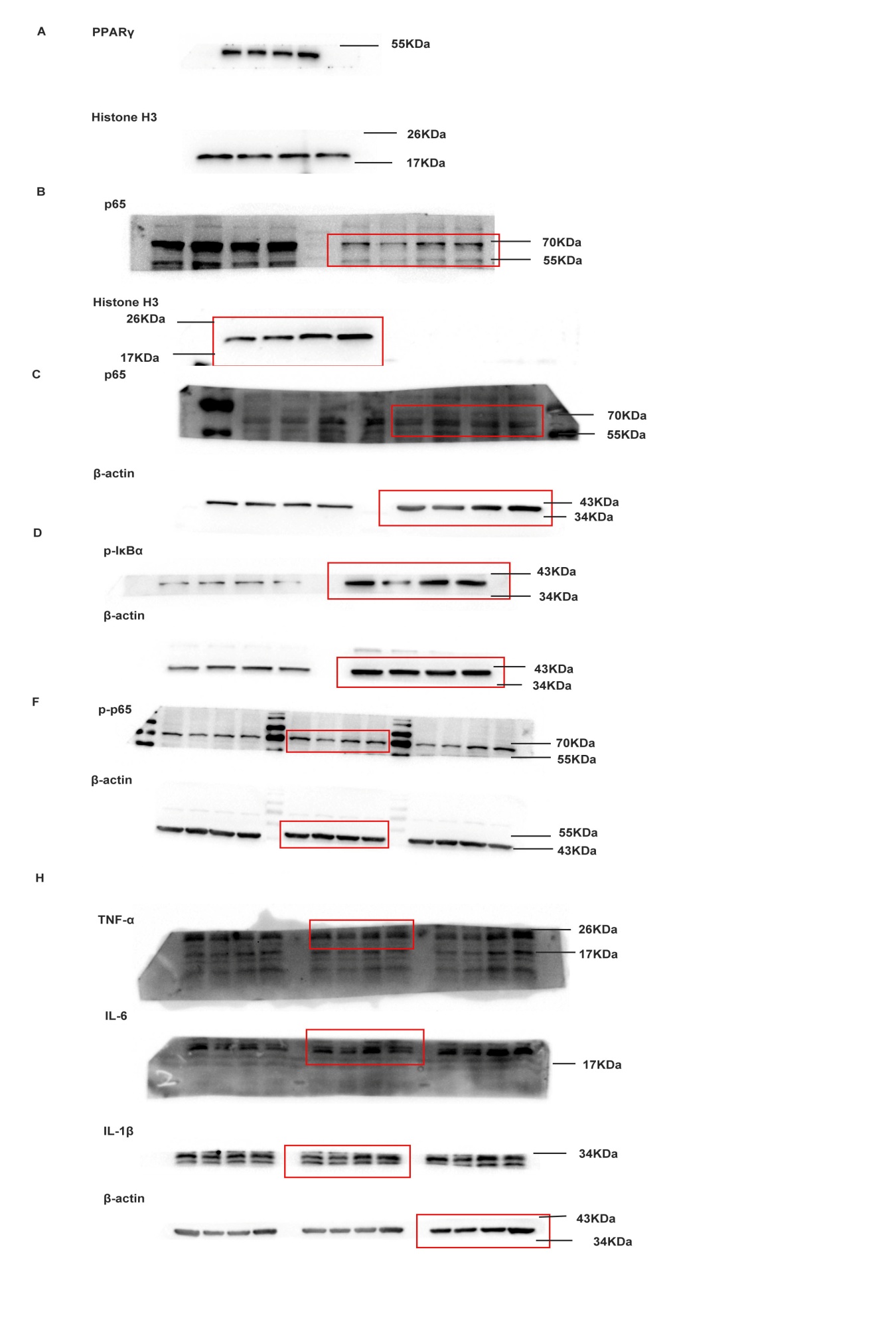
**

**Figure 7**

**
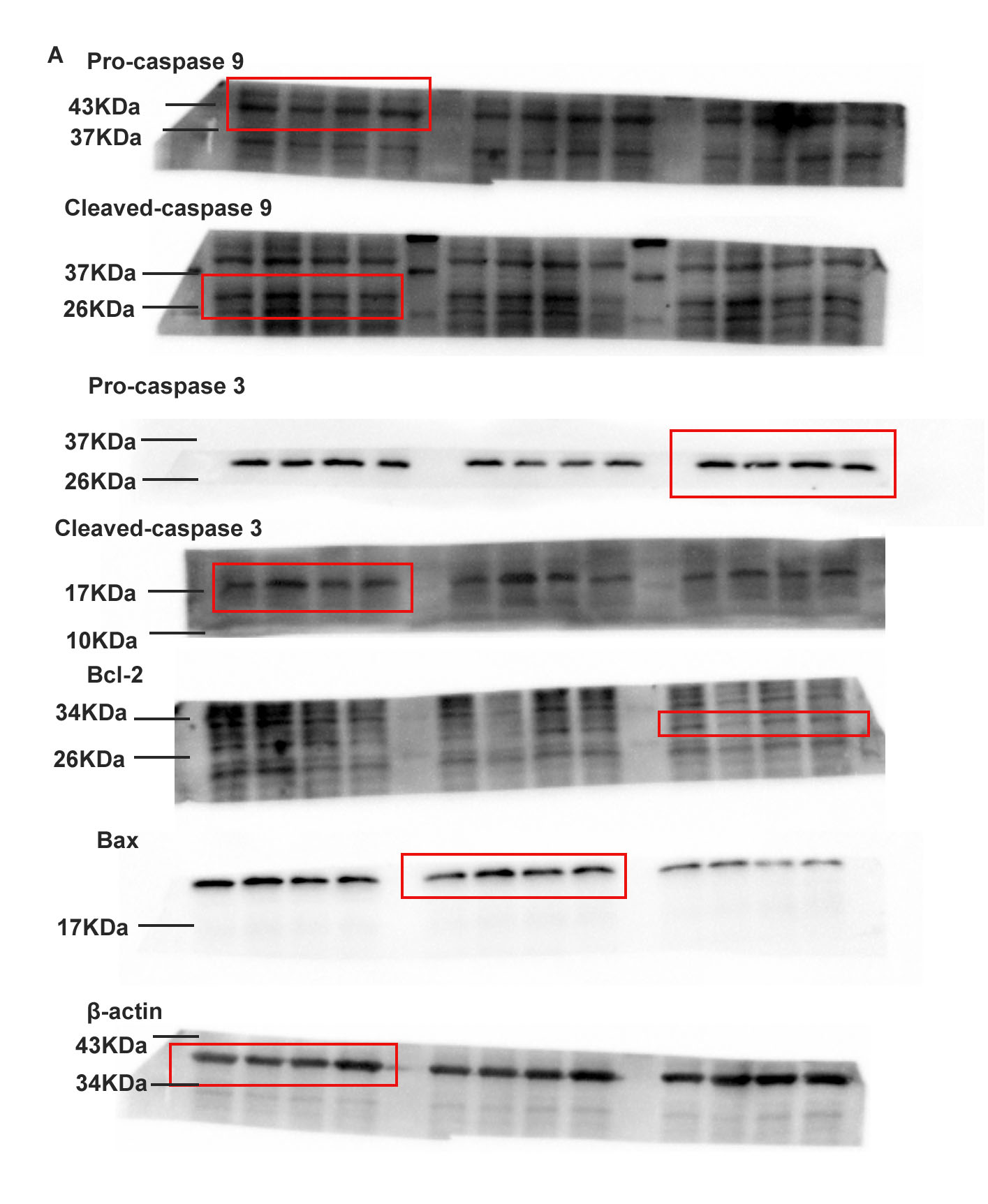
**

**Figure 8**

**
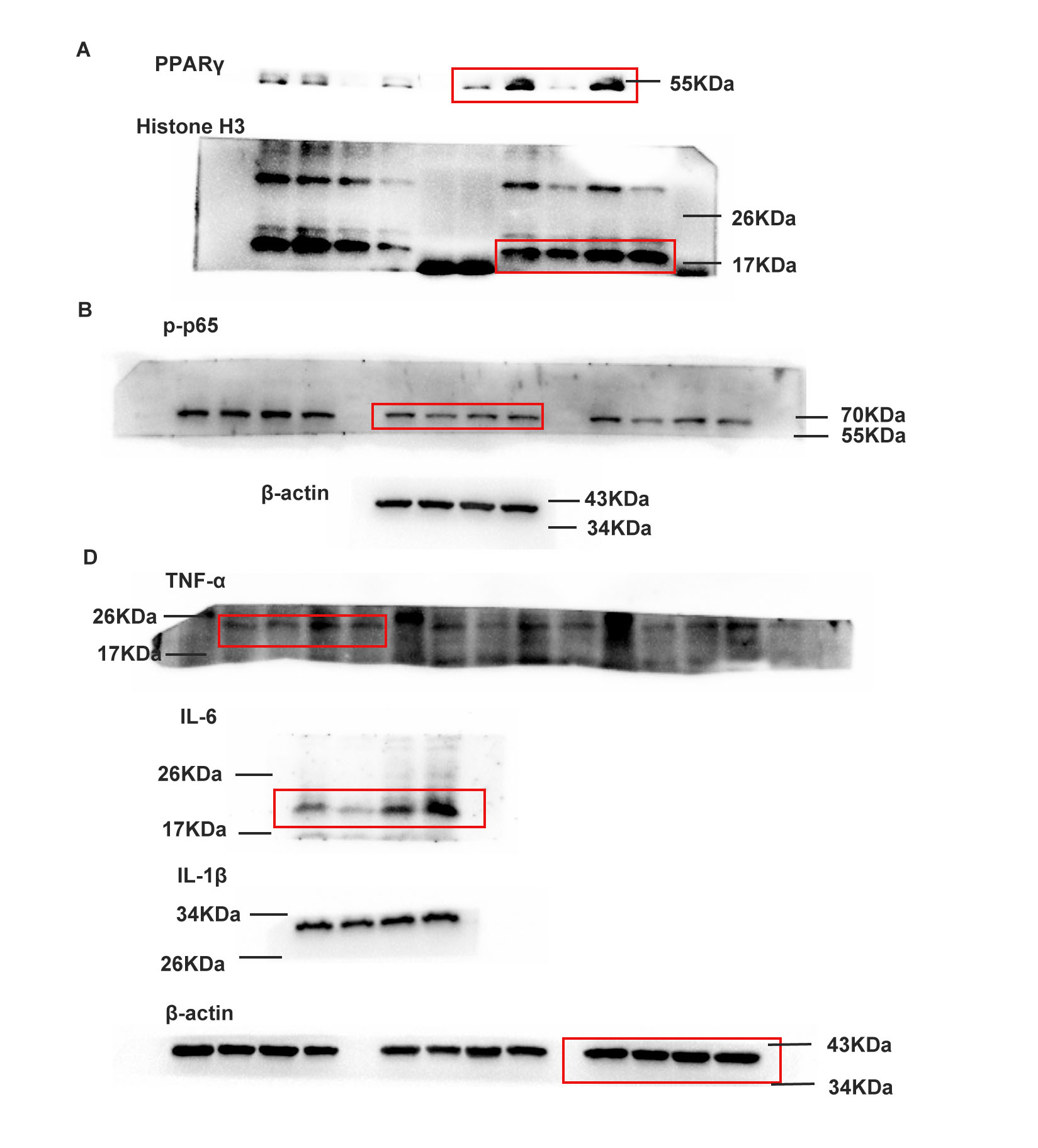
**

**Supplementary figure 4**

**
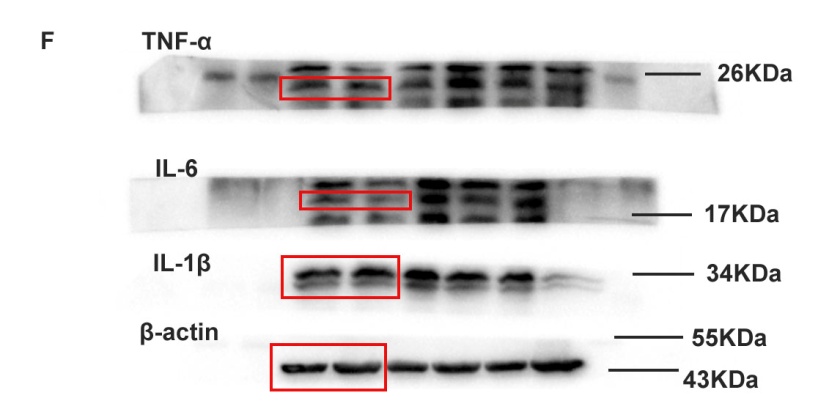
**

**Supplementary figure 6**

**
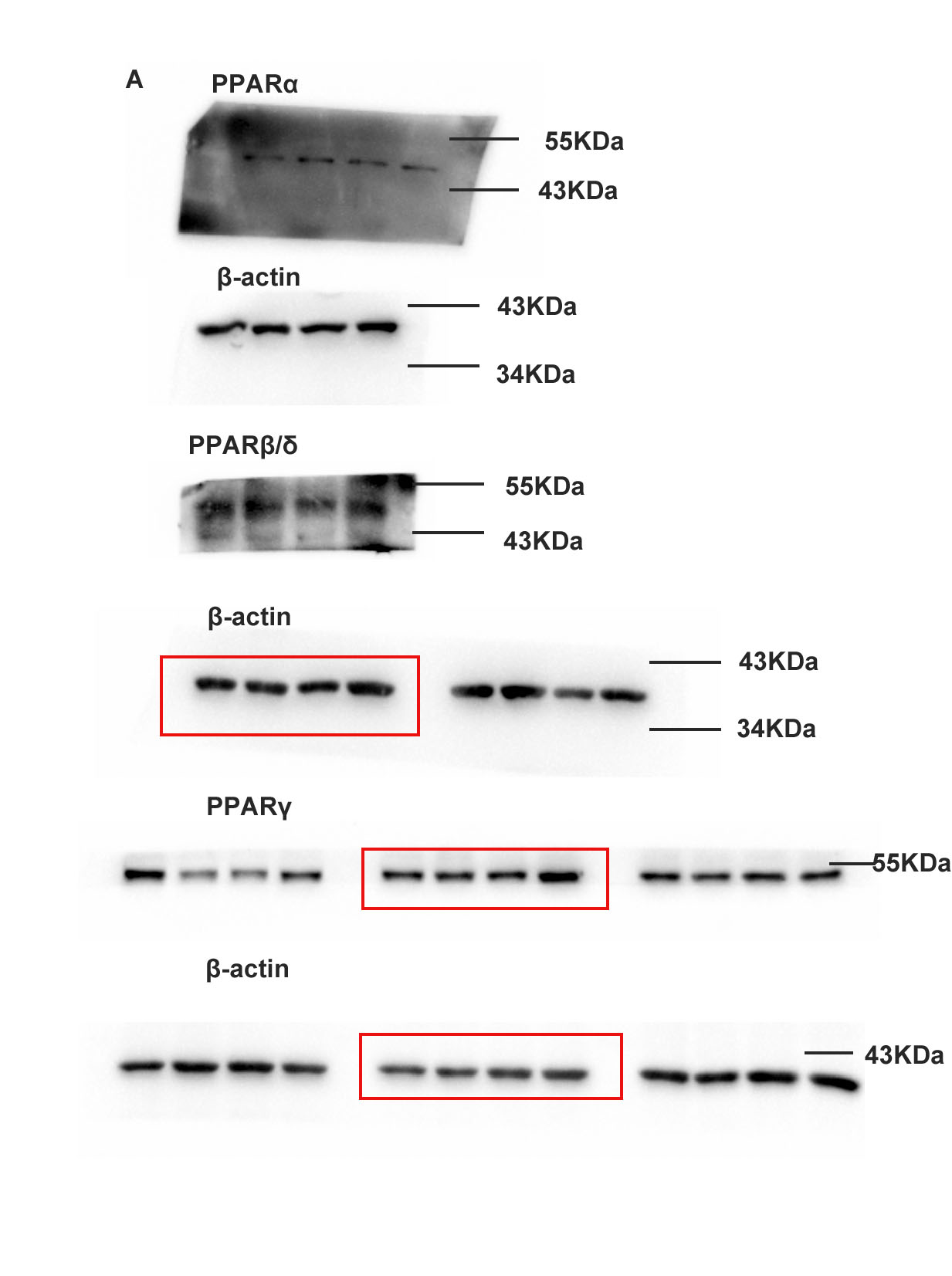
**

**Supplementary figure 7**

**
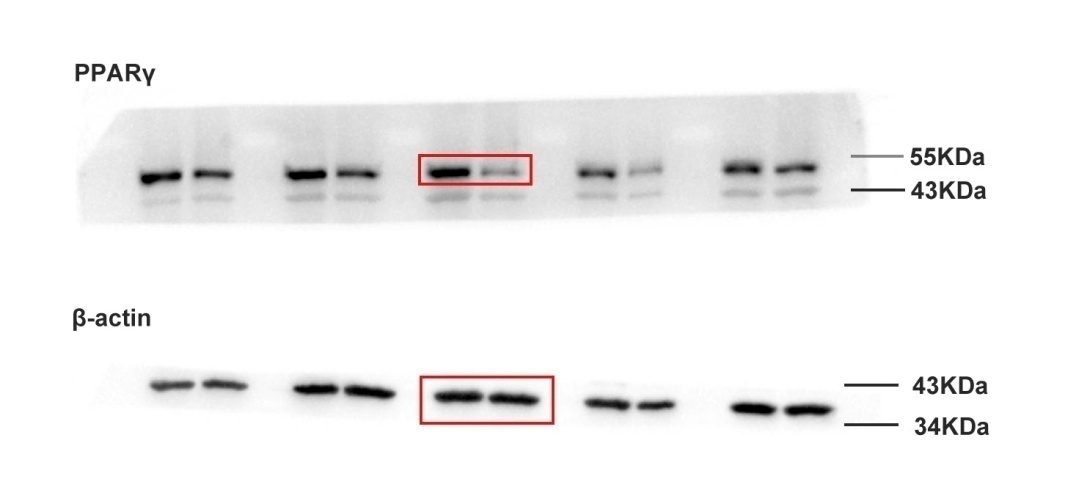
**

**Supplementary figure 8**

**
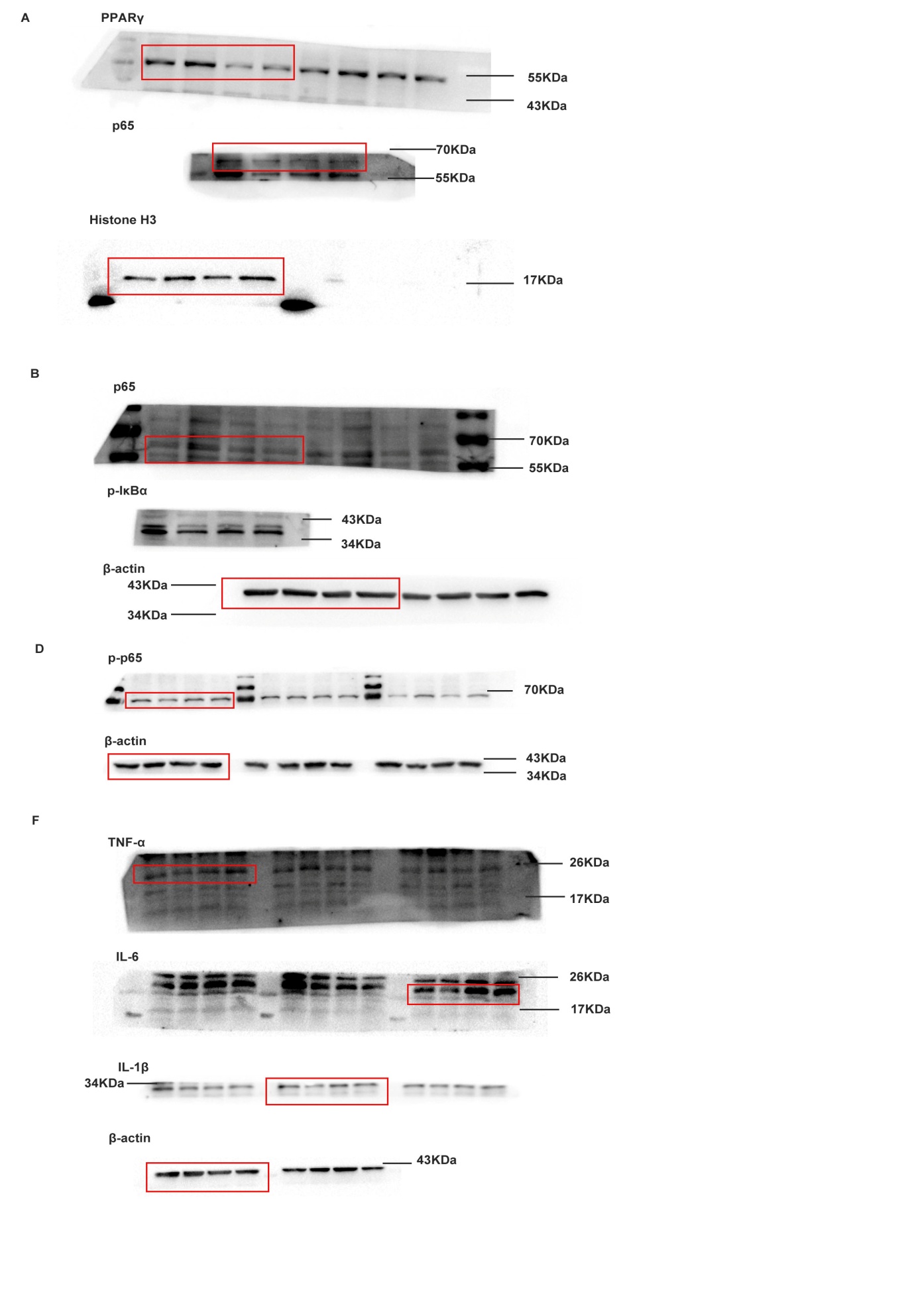
**

**Entire blot**

**
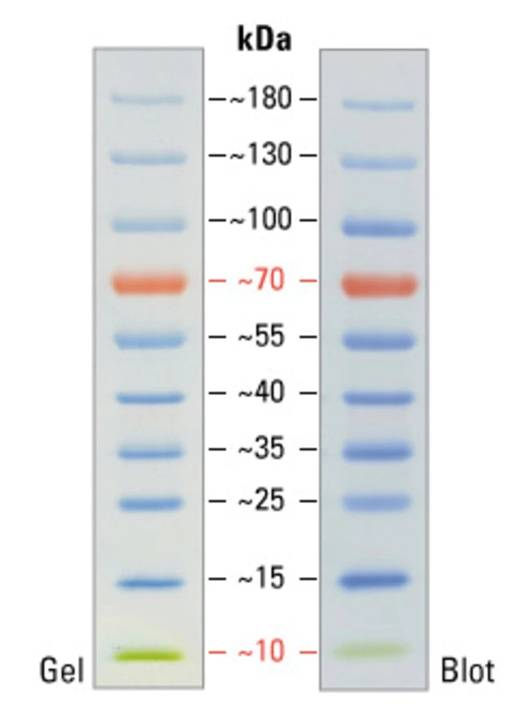
**

**
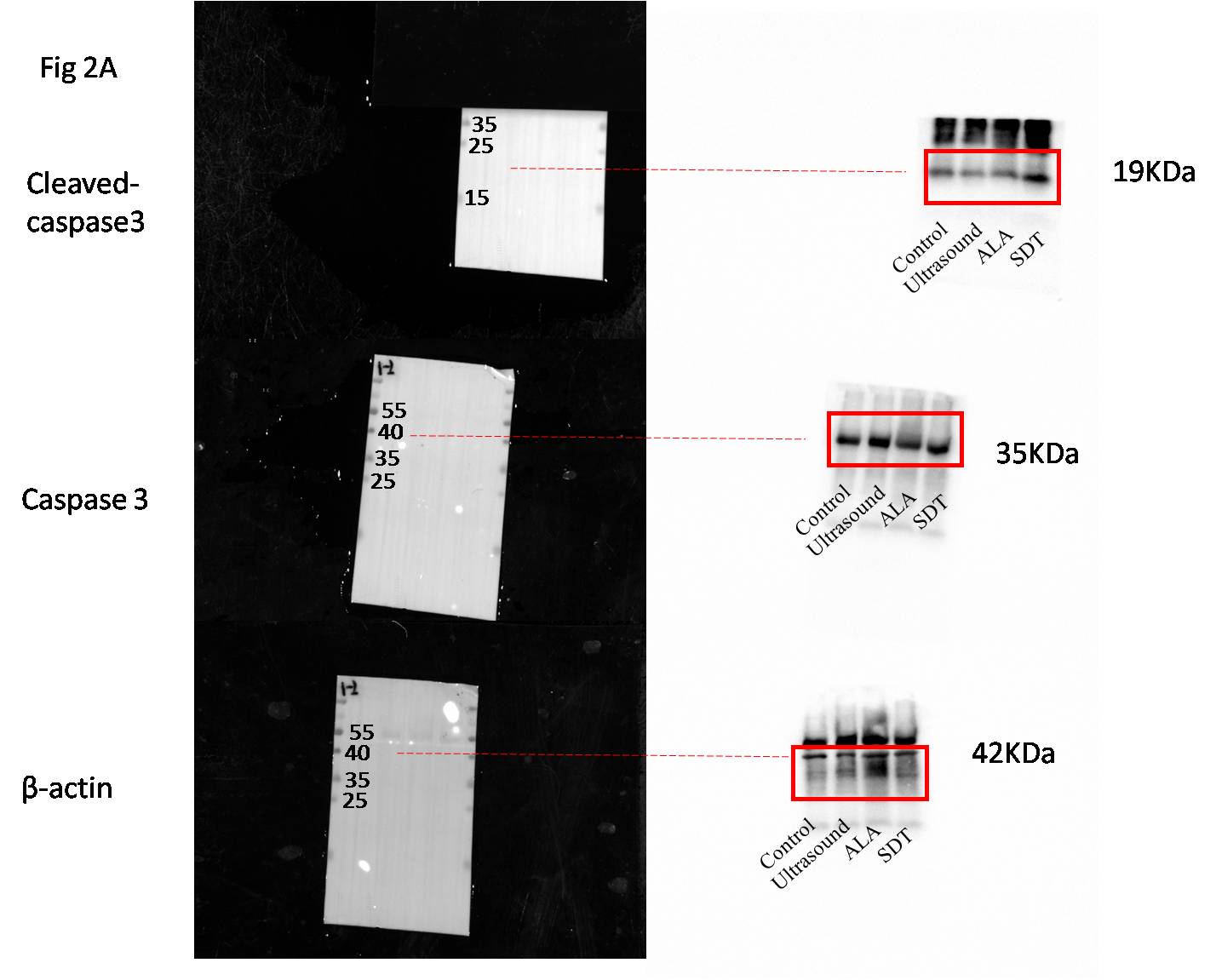
**

**
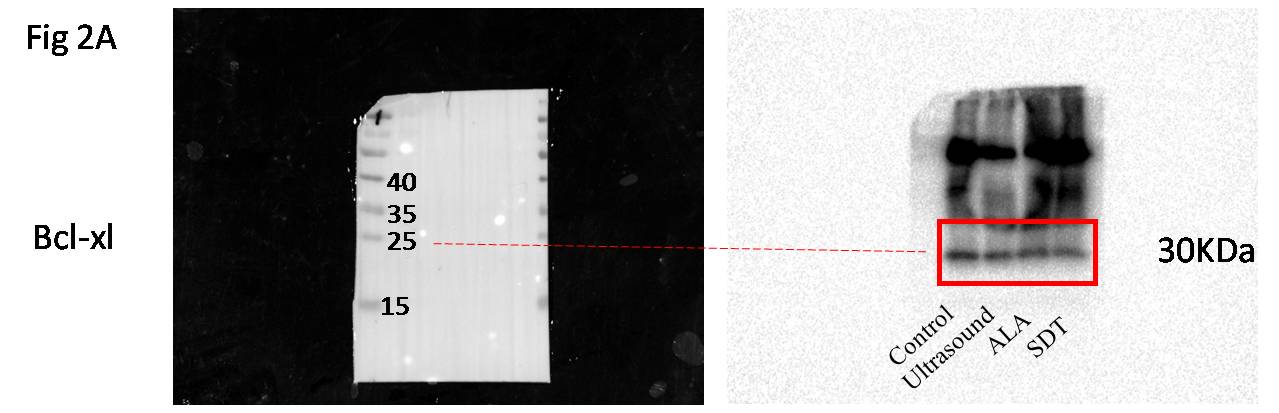
**

**
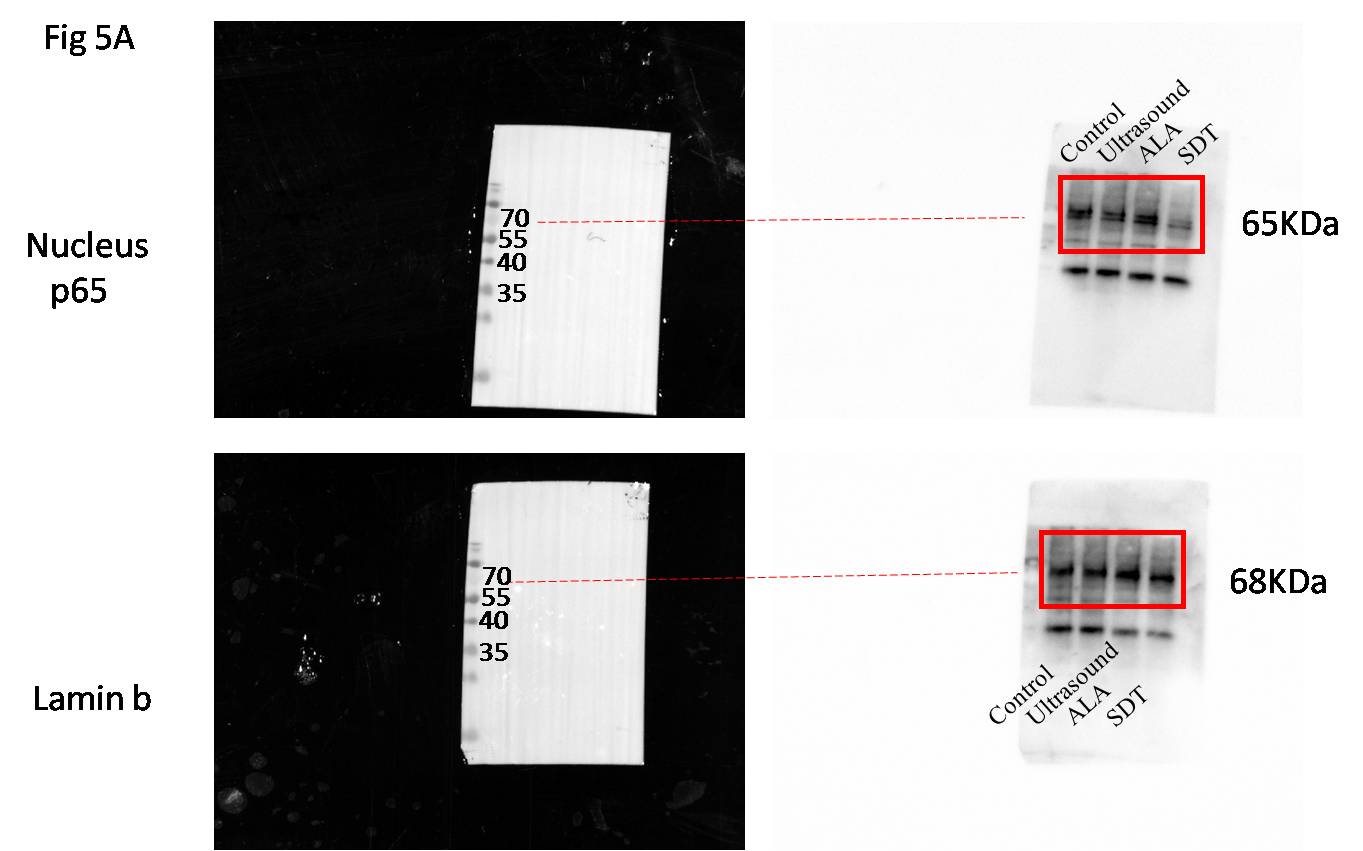
**

**
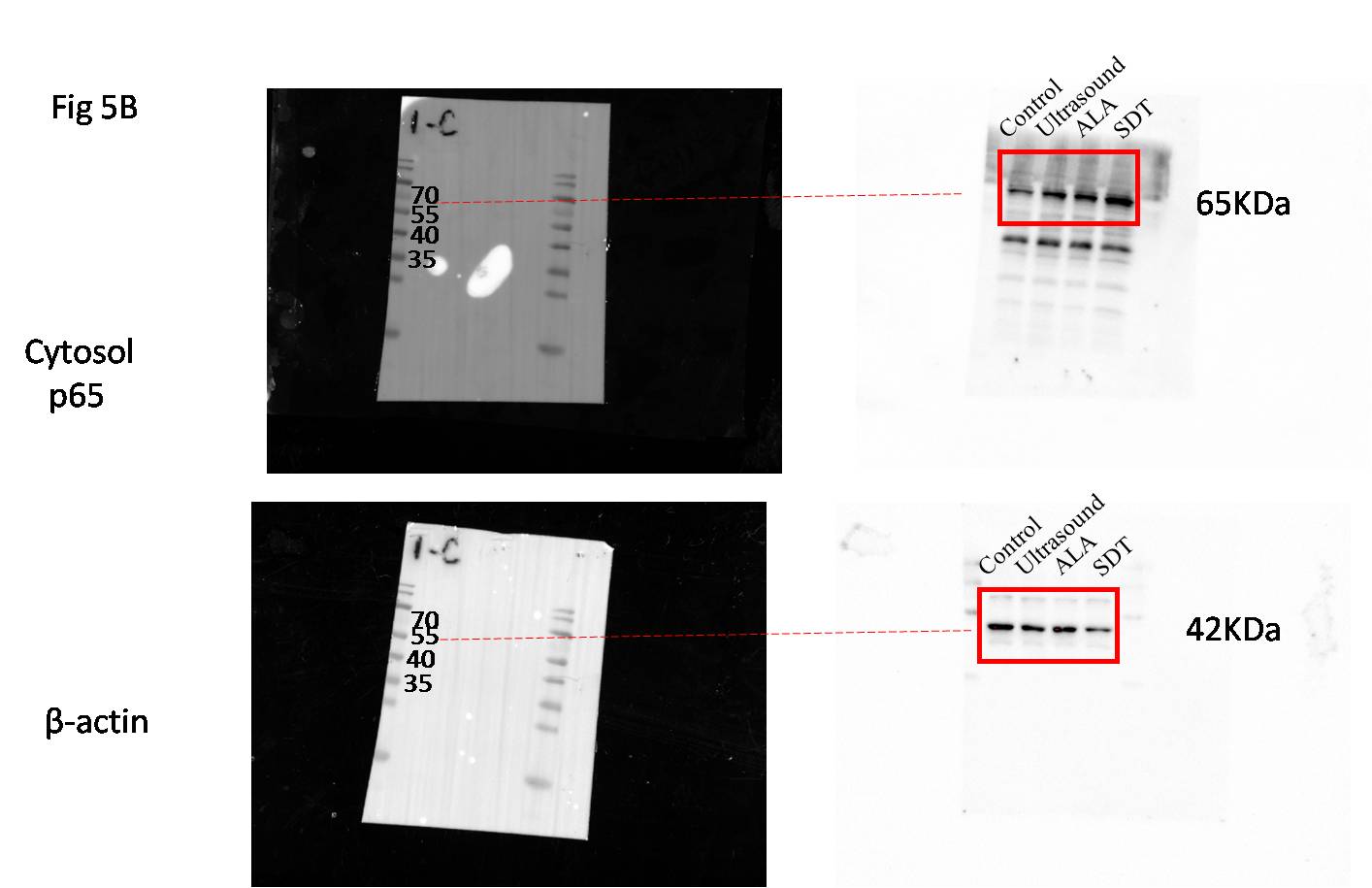
**

**
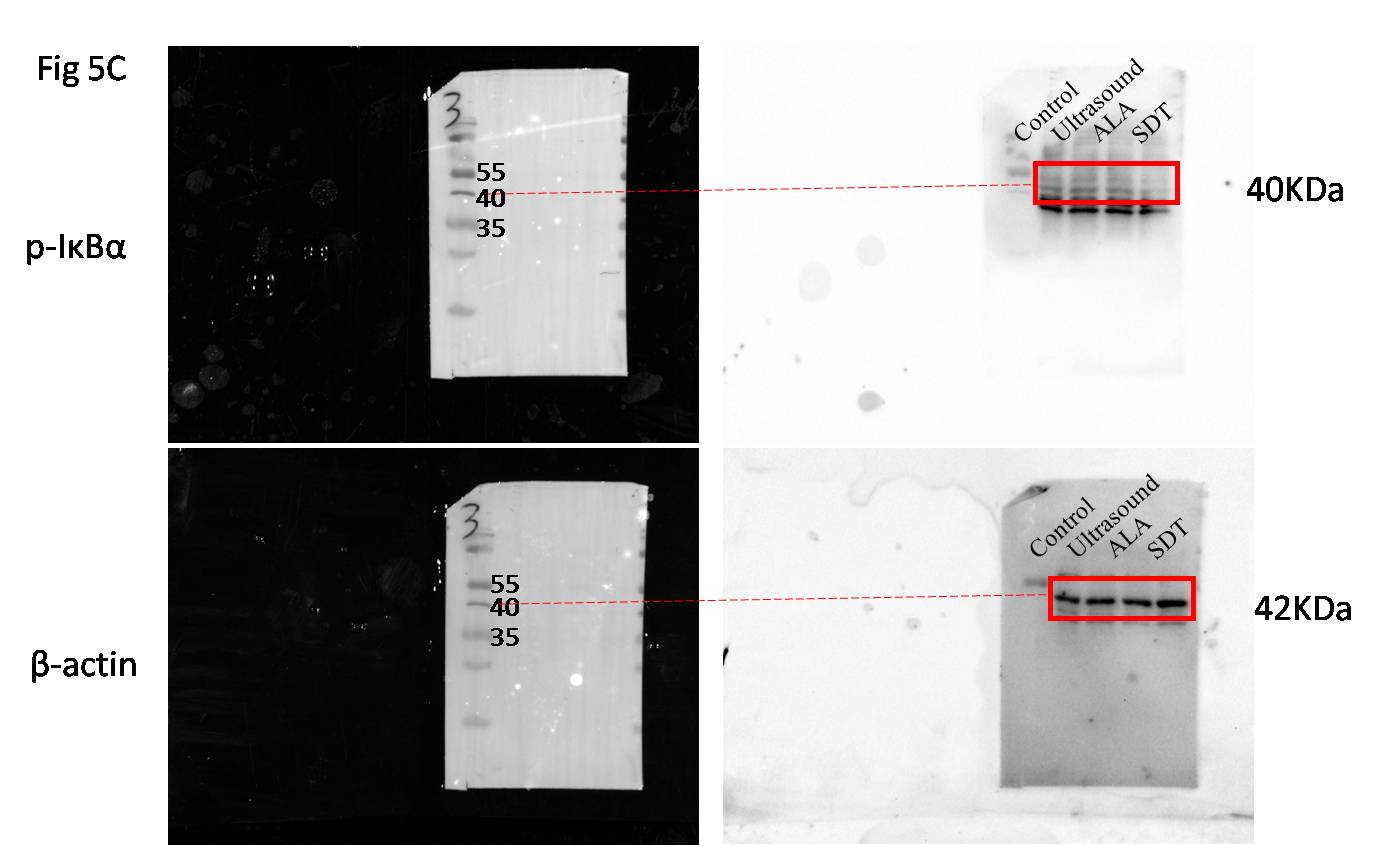
**

**
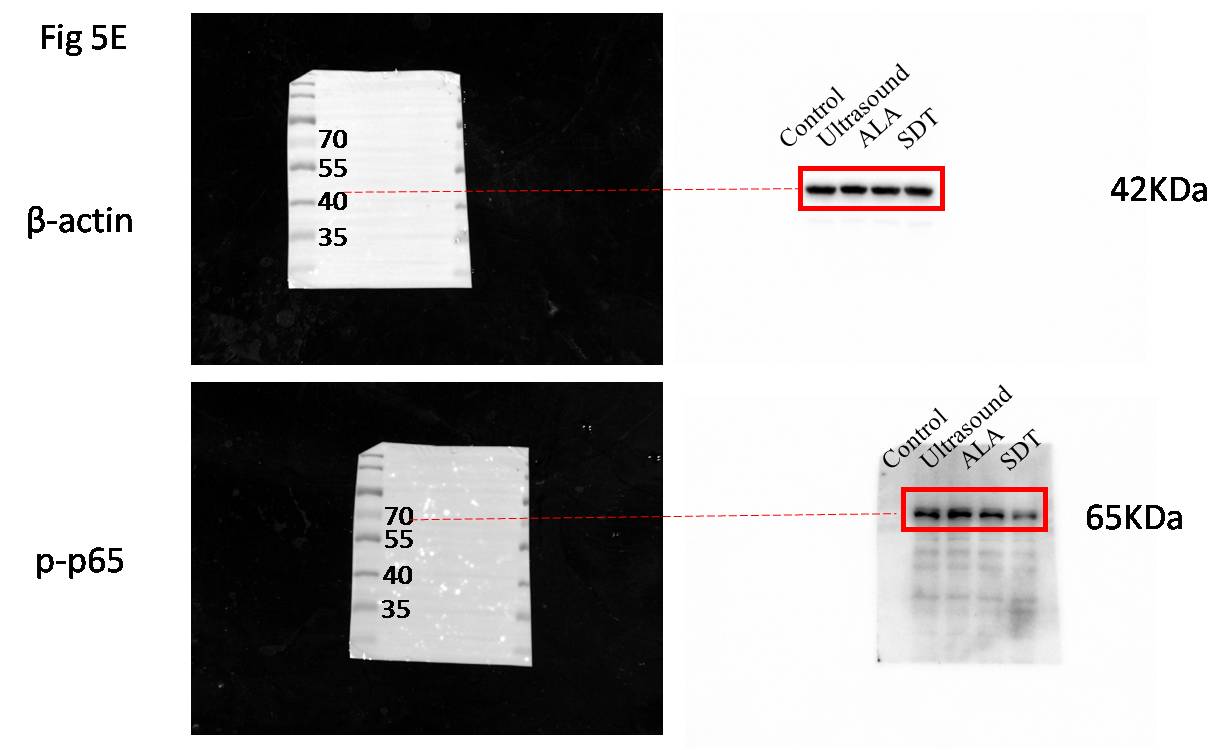
**

**
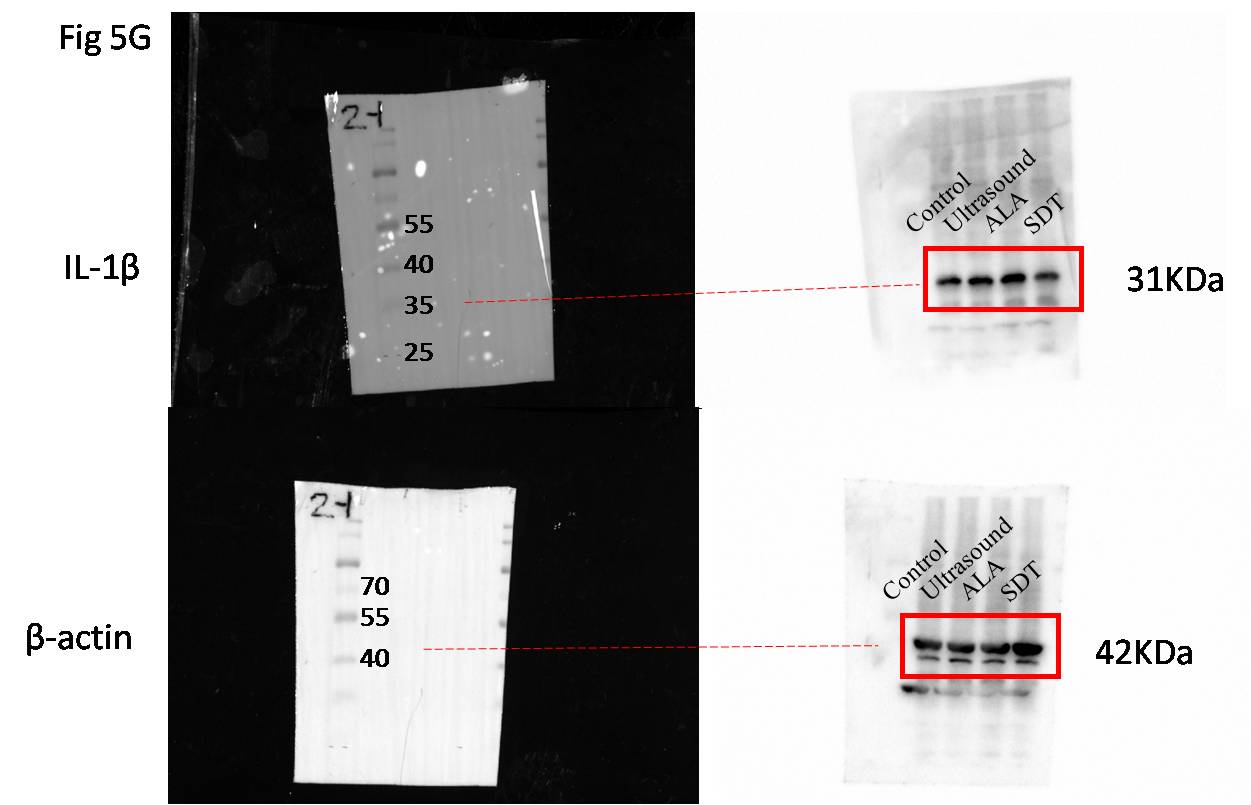
**

**
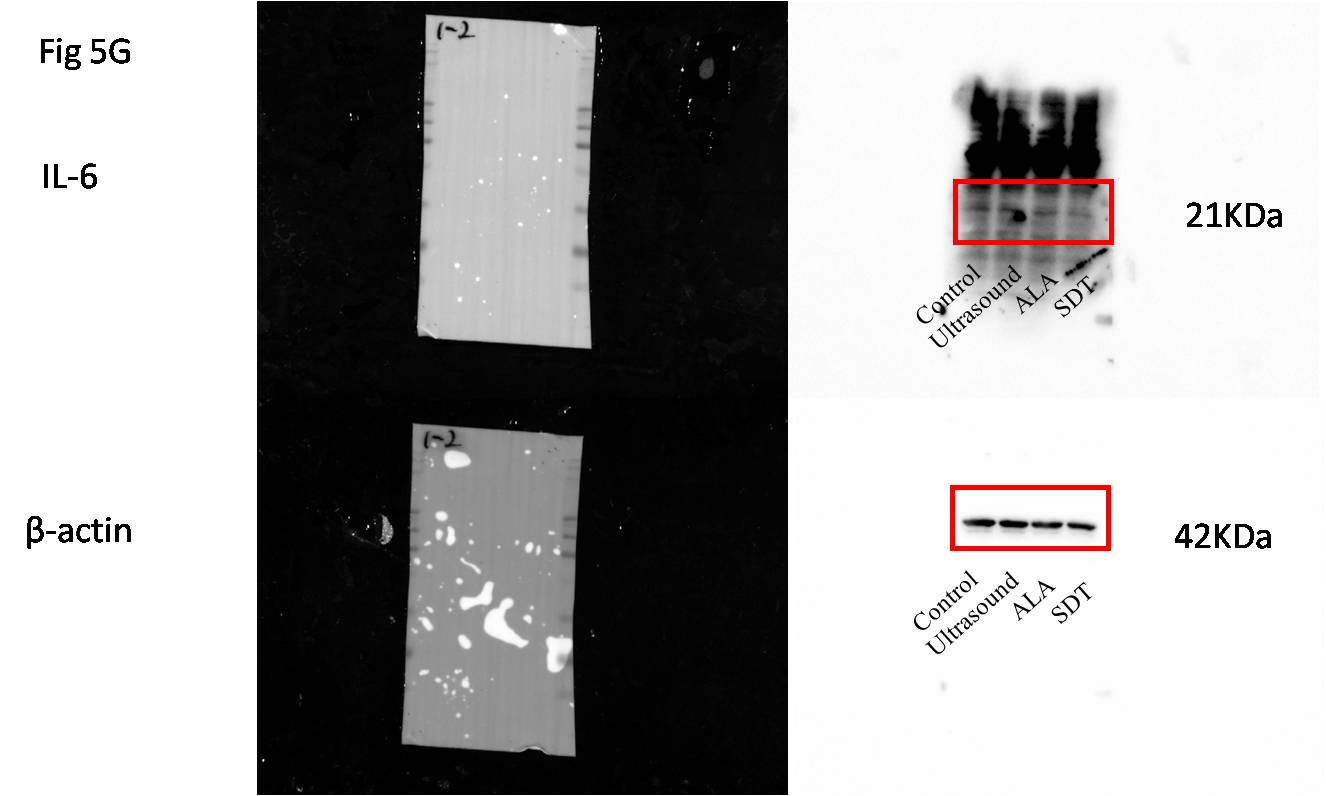
**

**
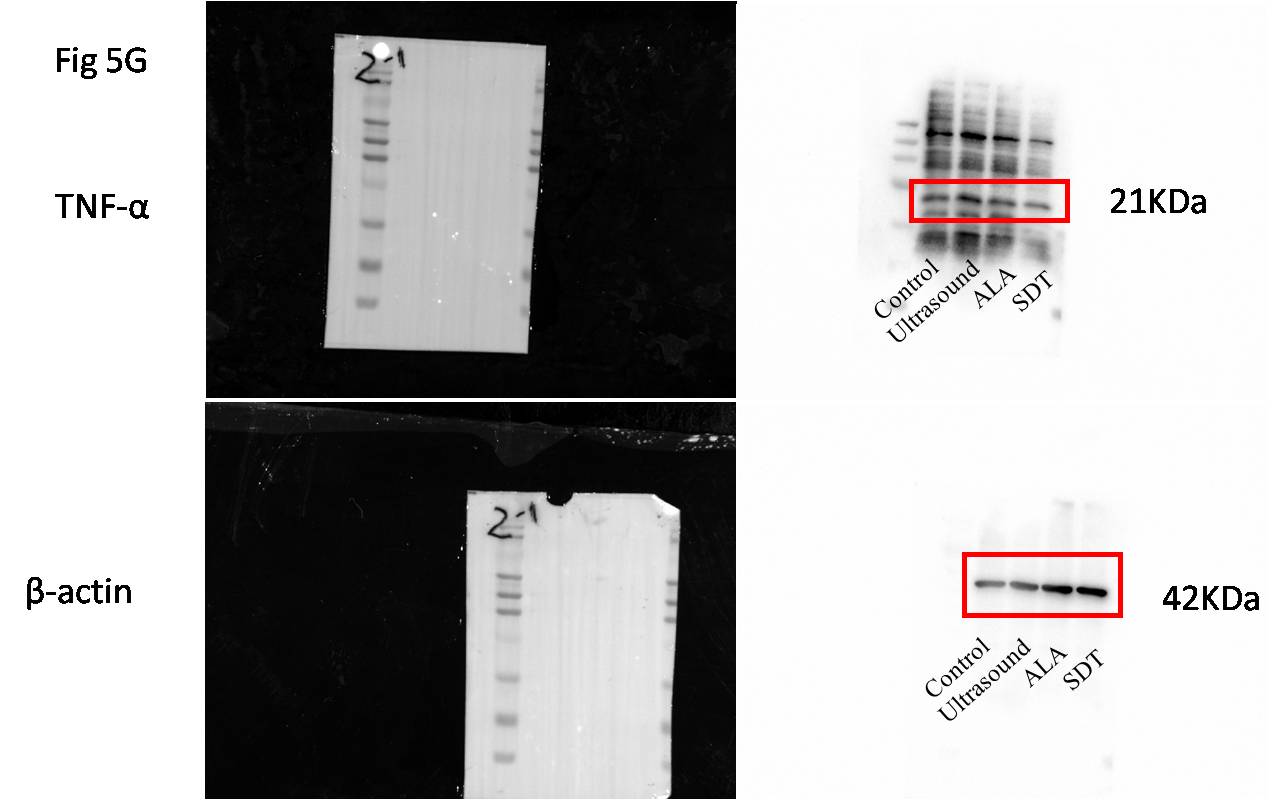
**

**
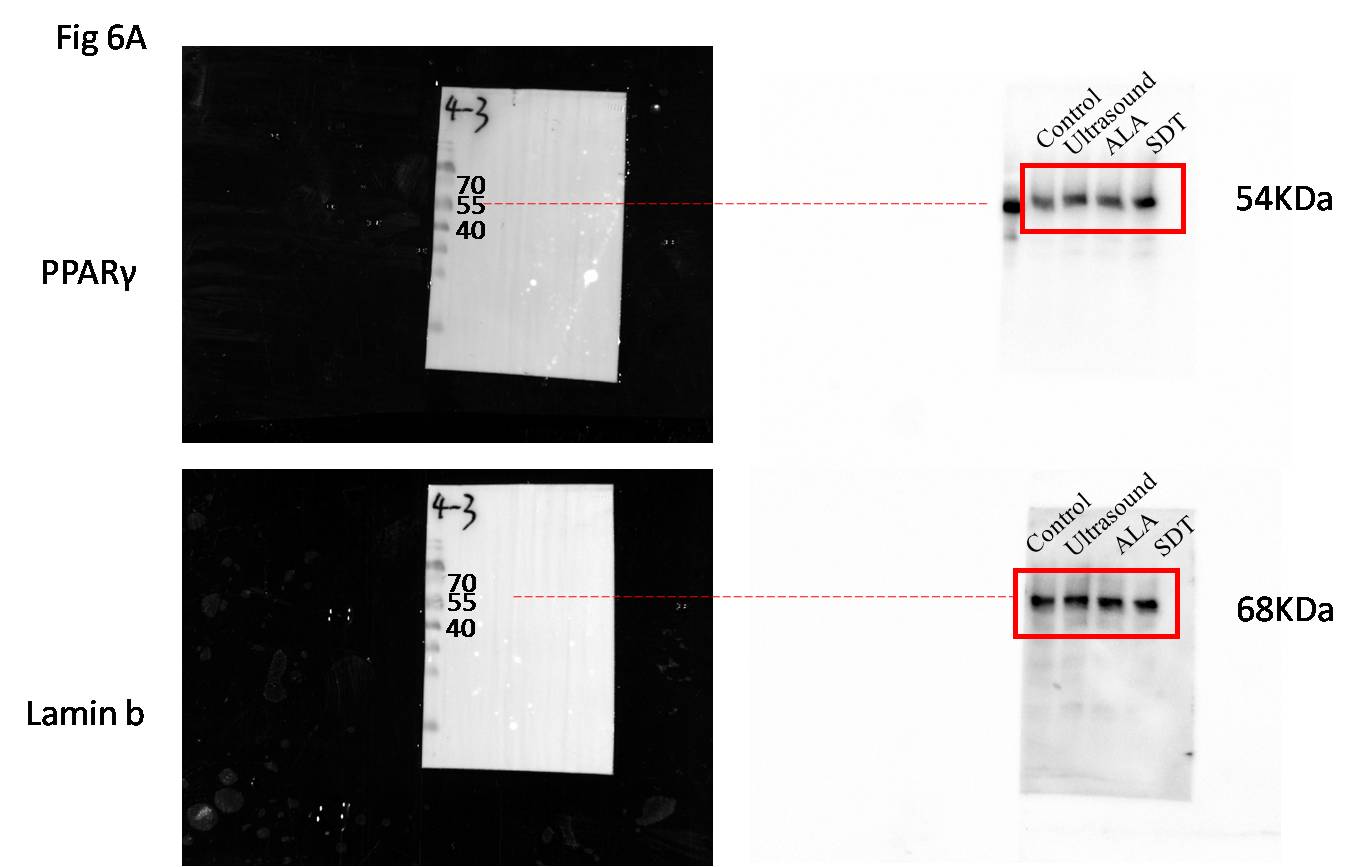
**

**
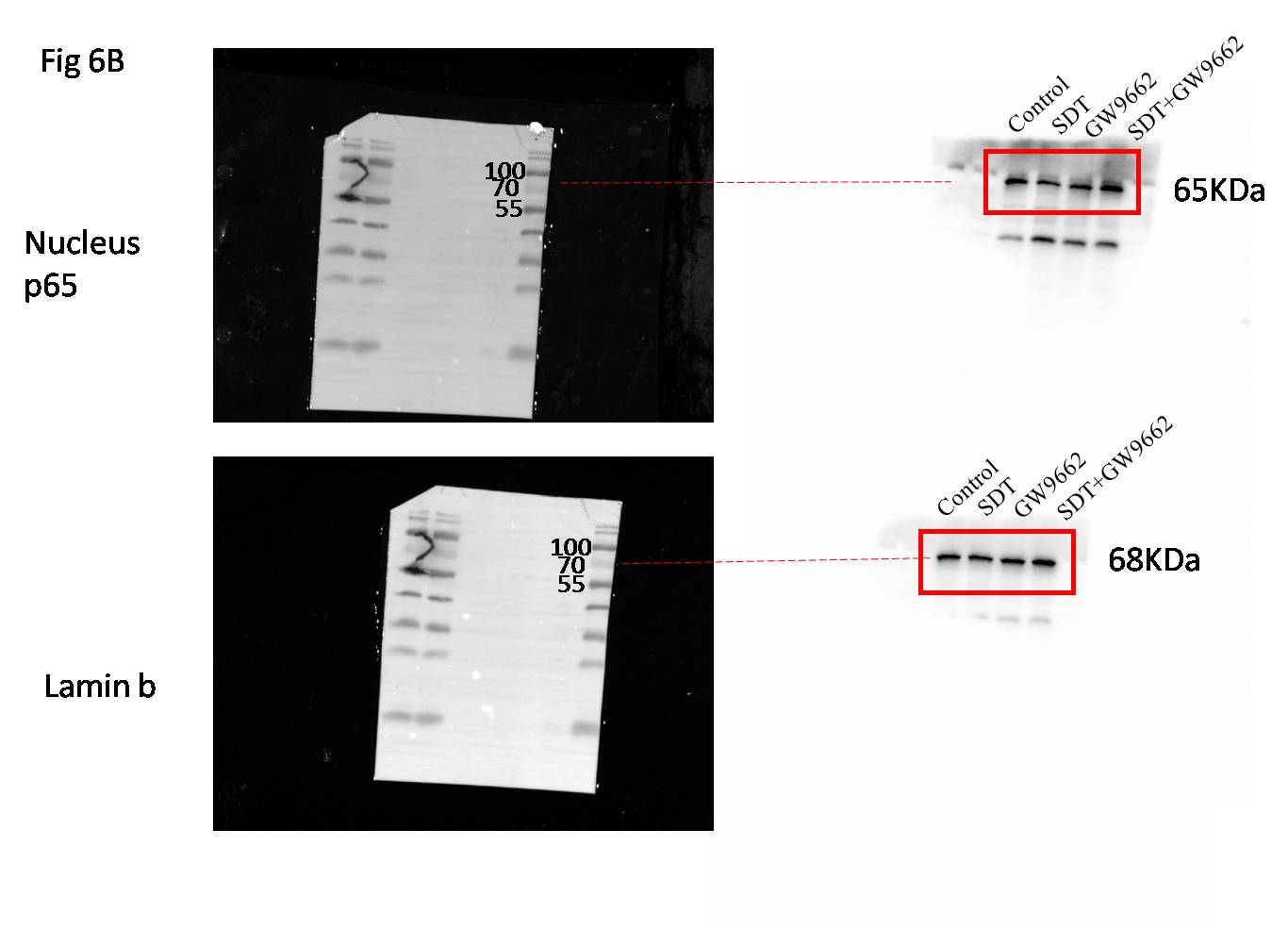
**

**
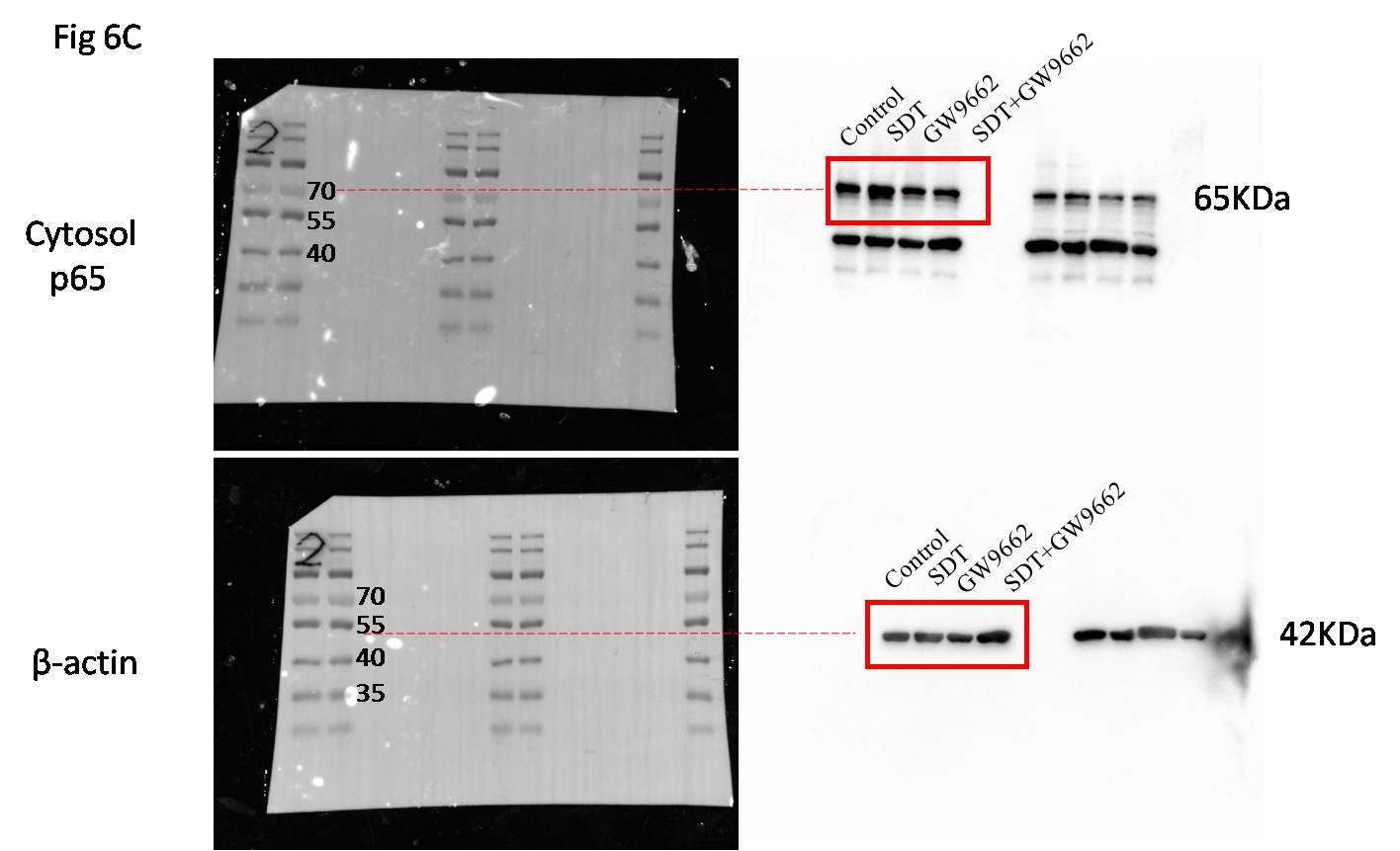
**

**
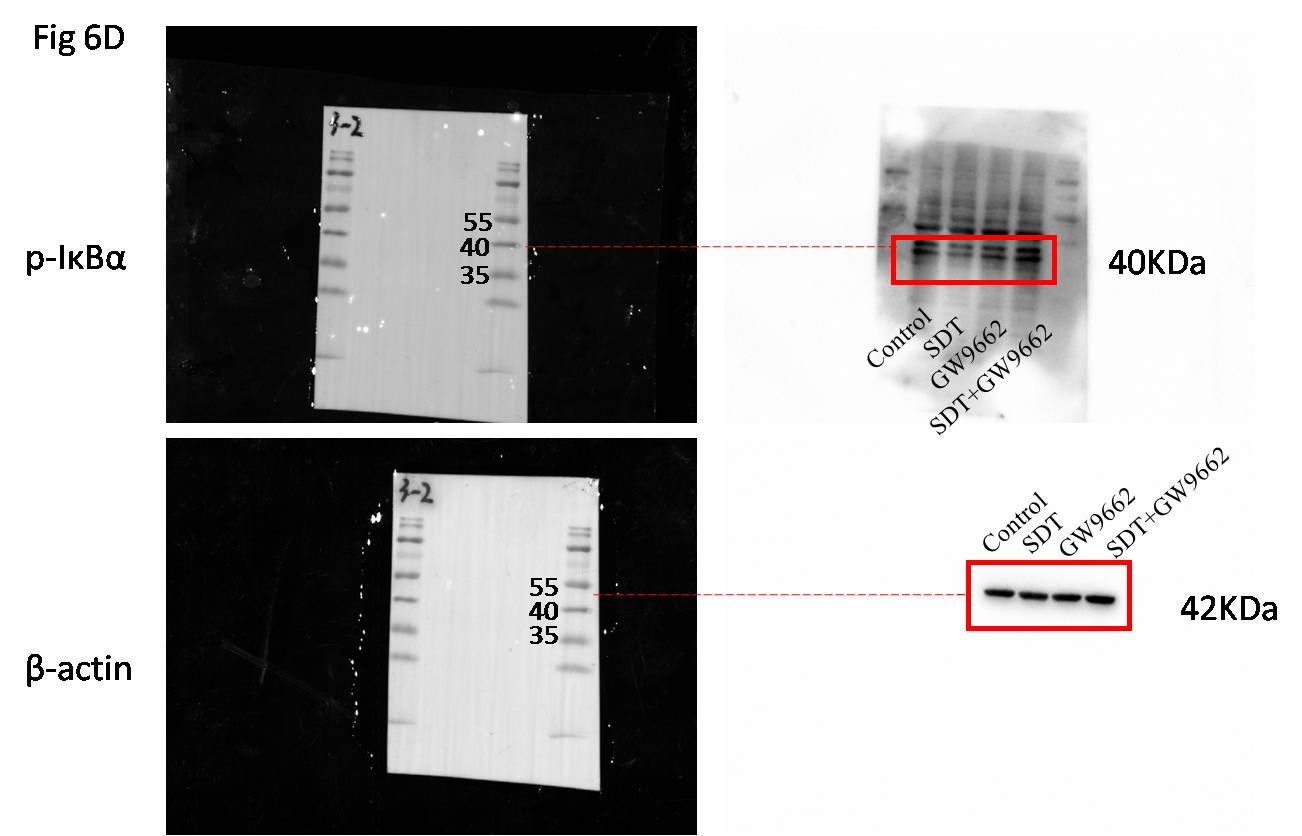
**

**
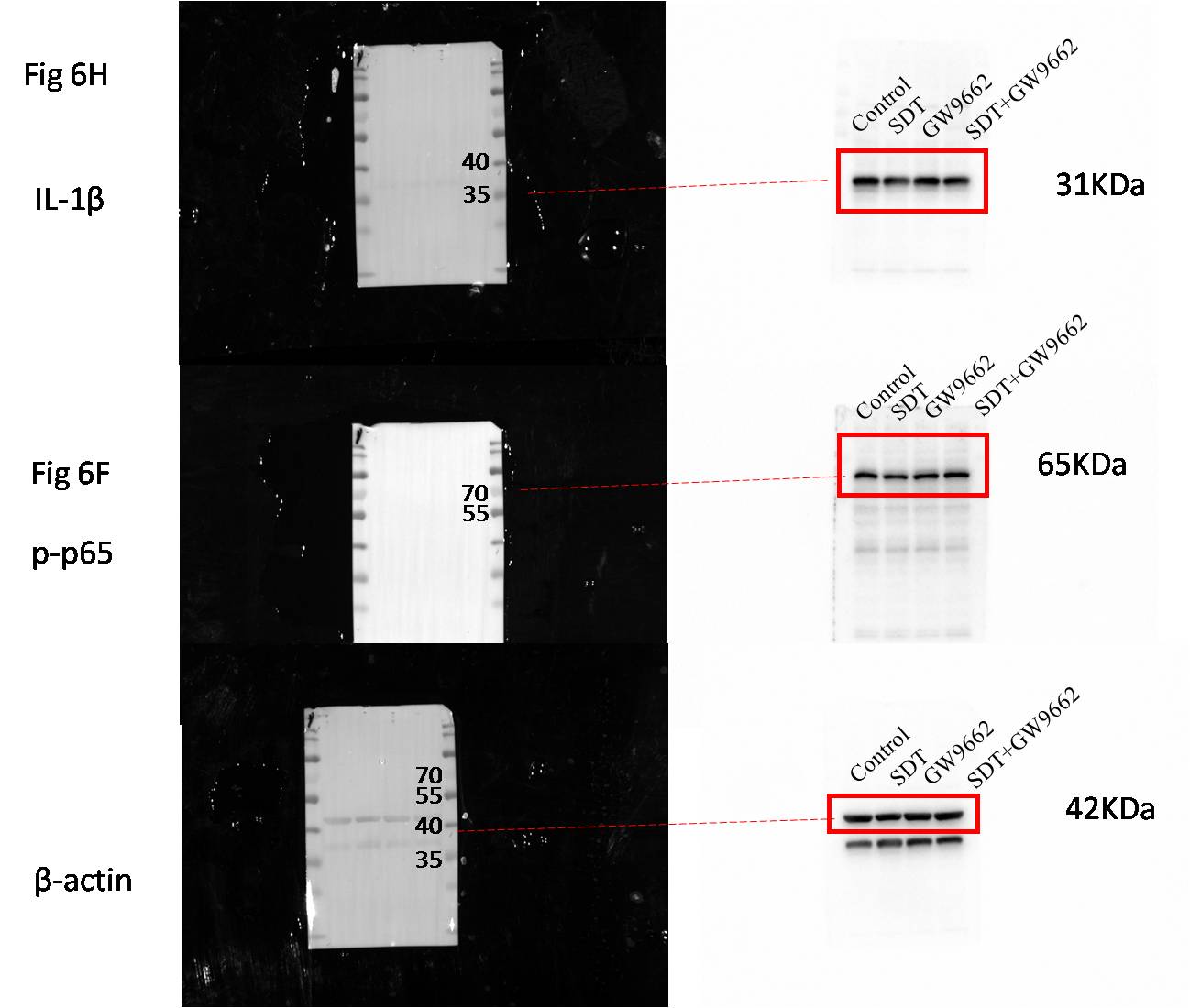
**

**
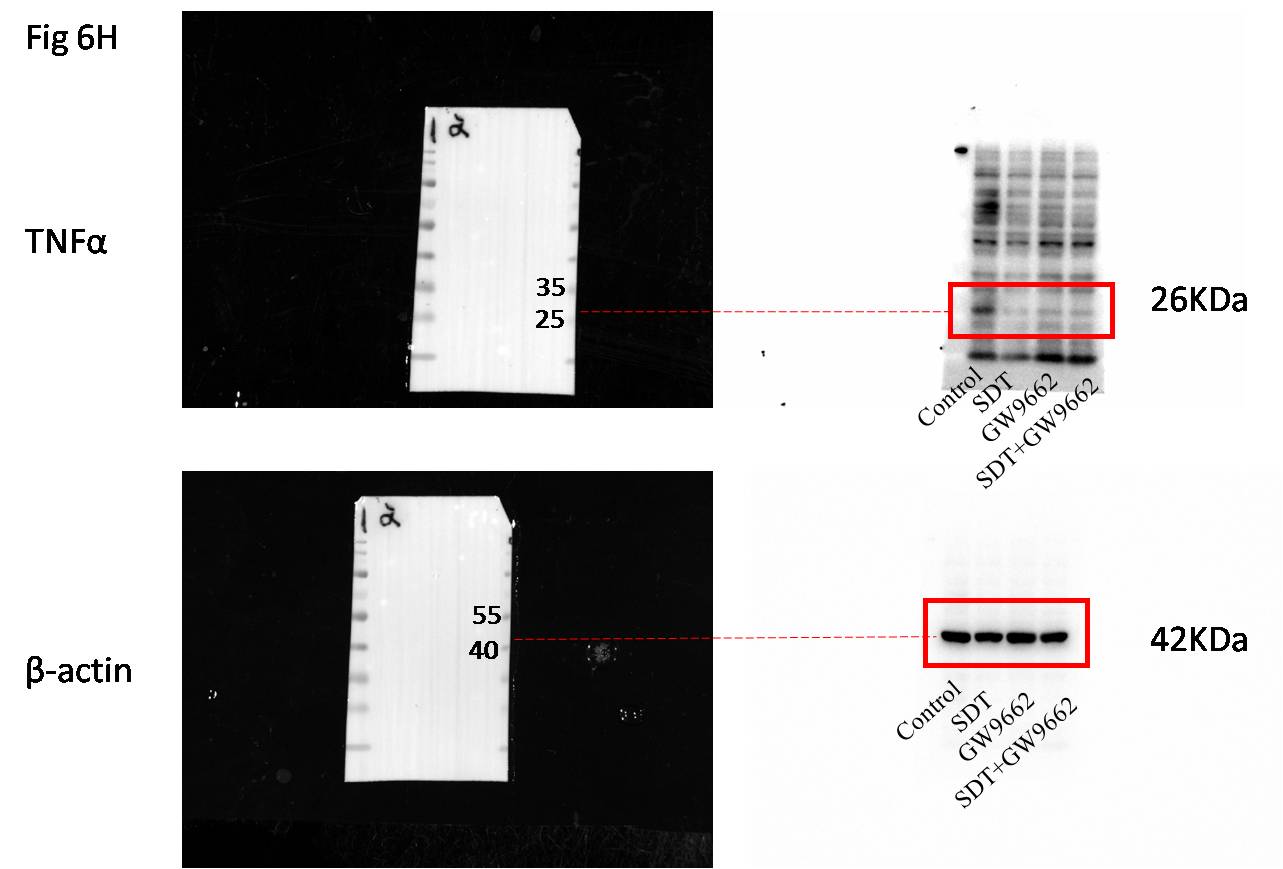
**

**
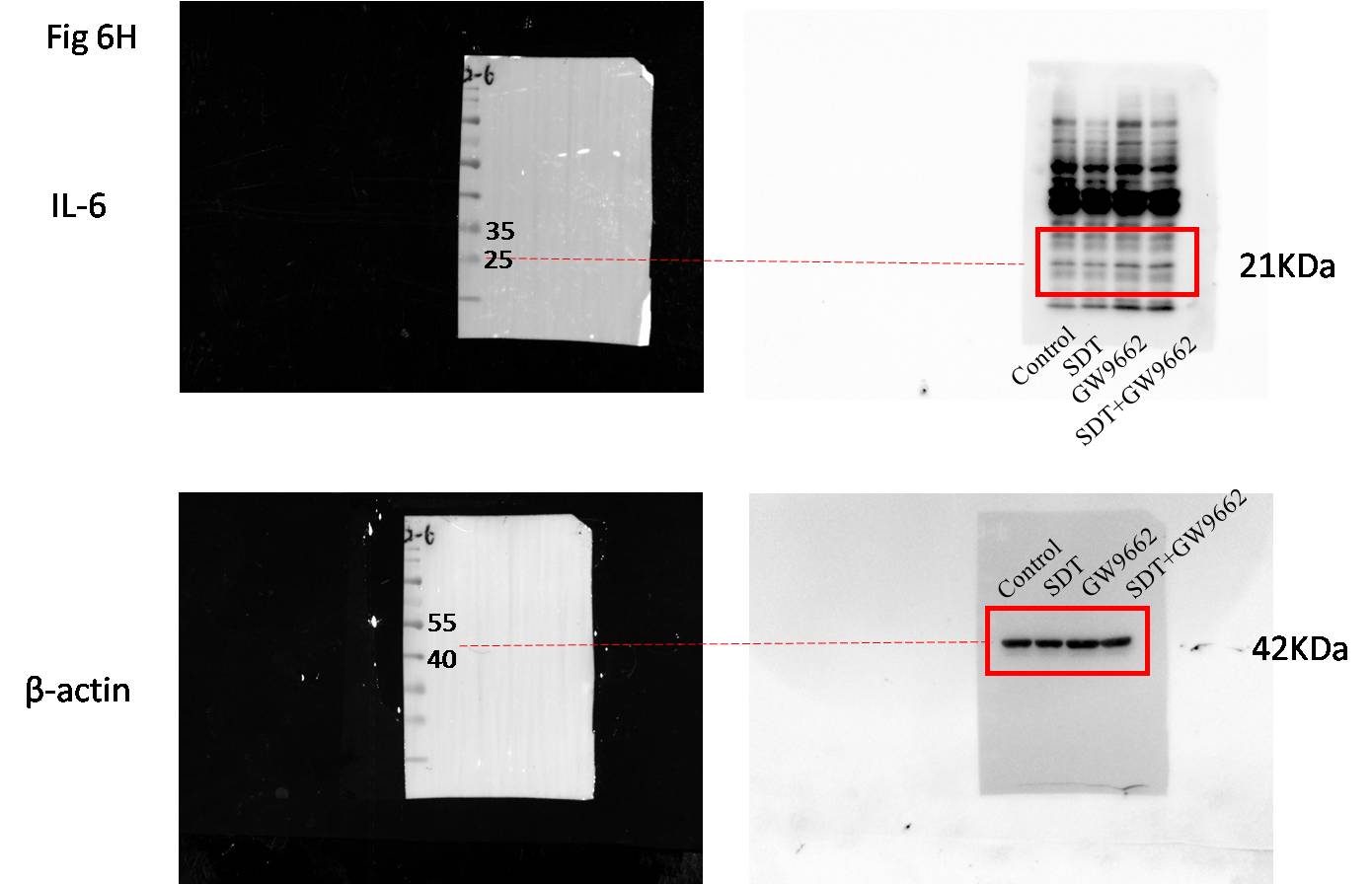
**

**
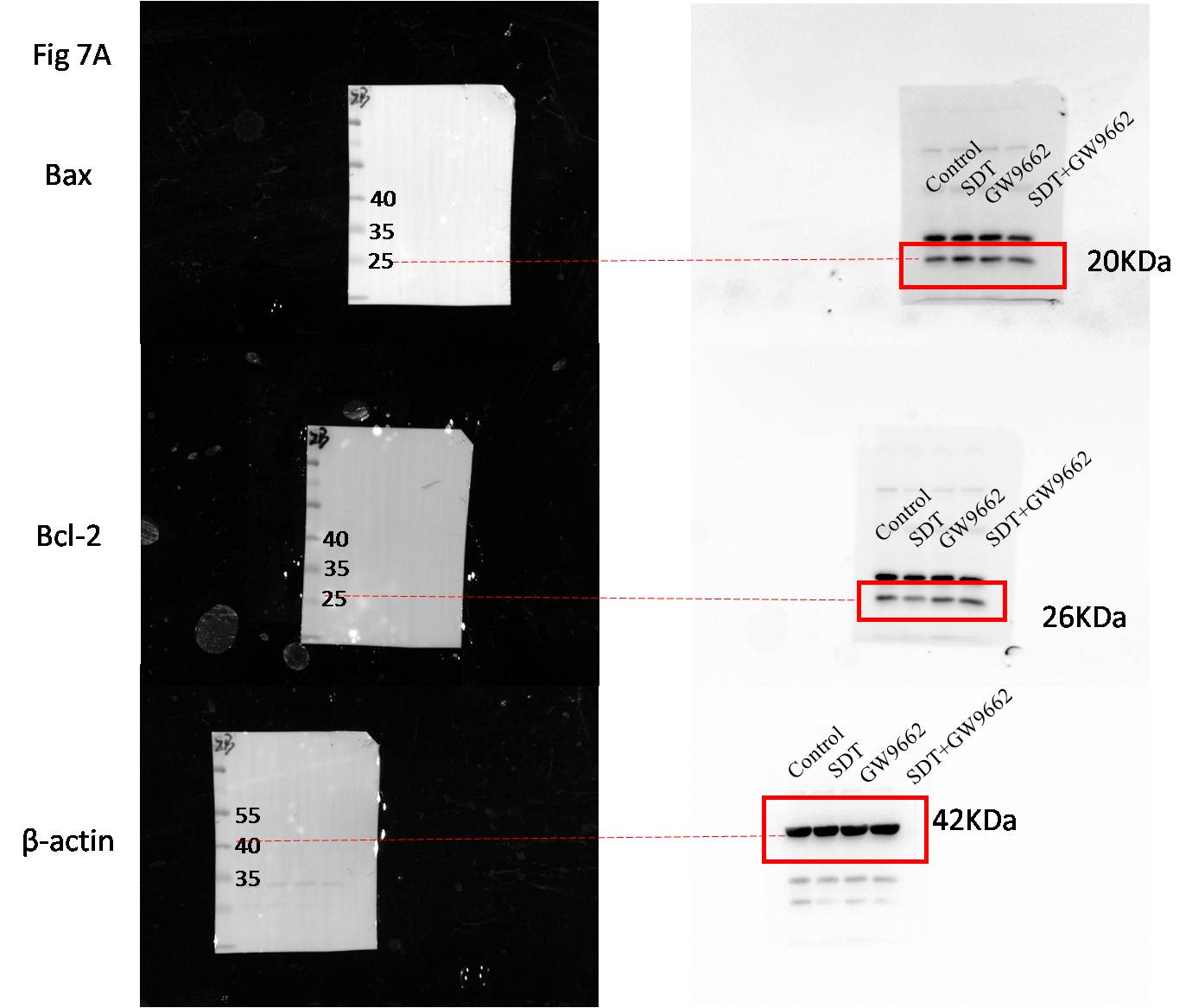
**

**
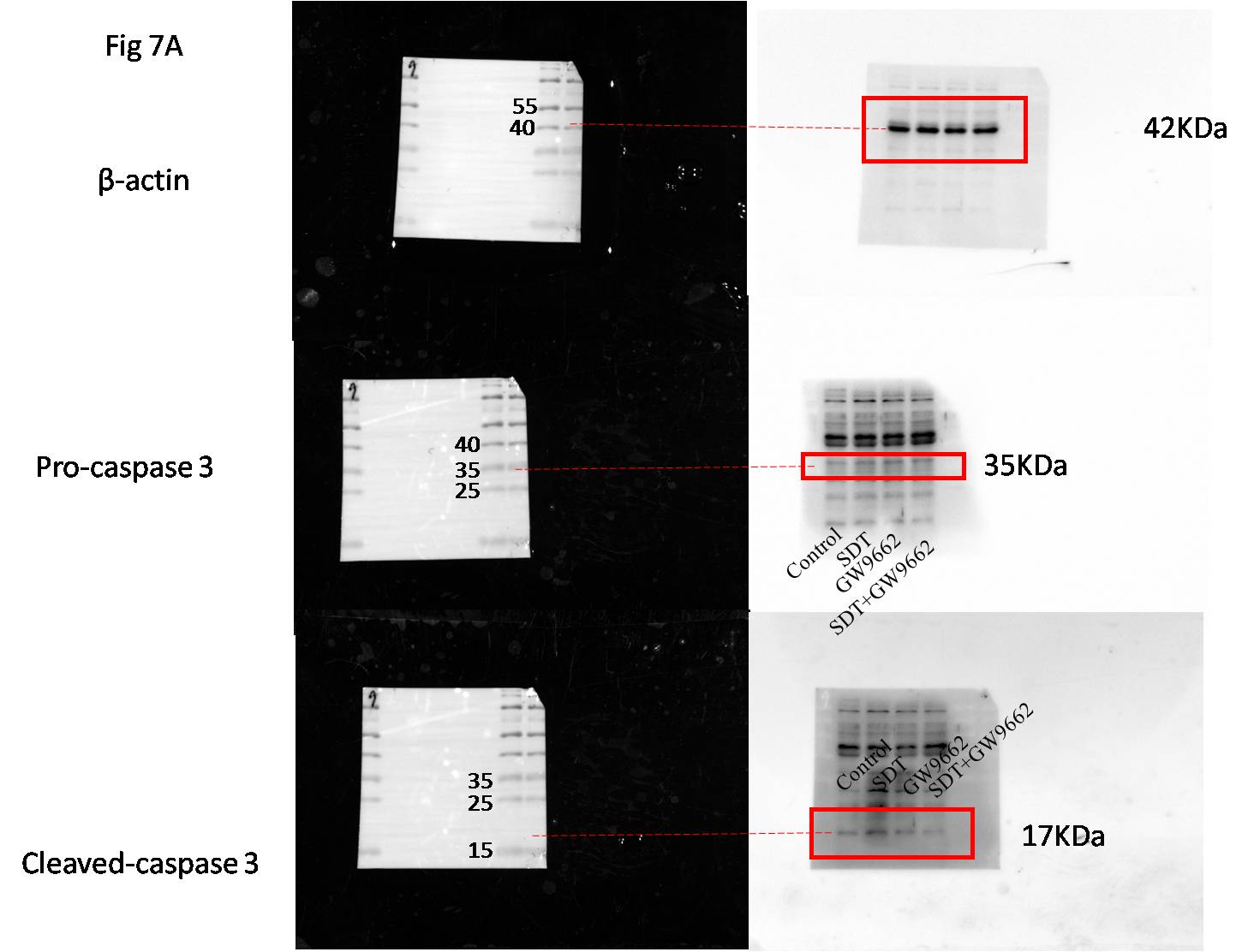
**

**
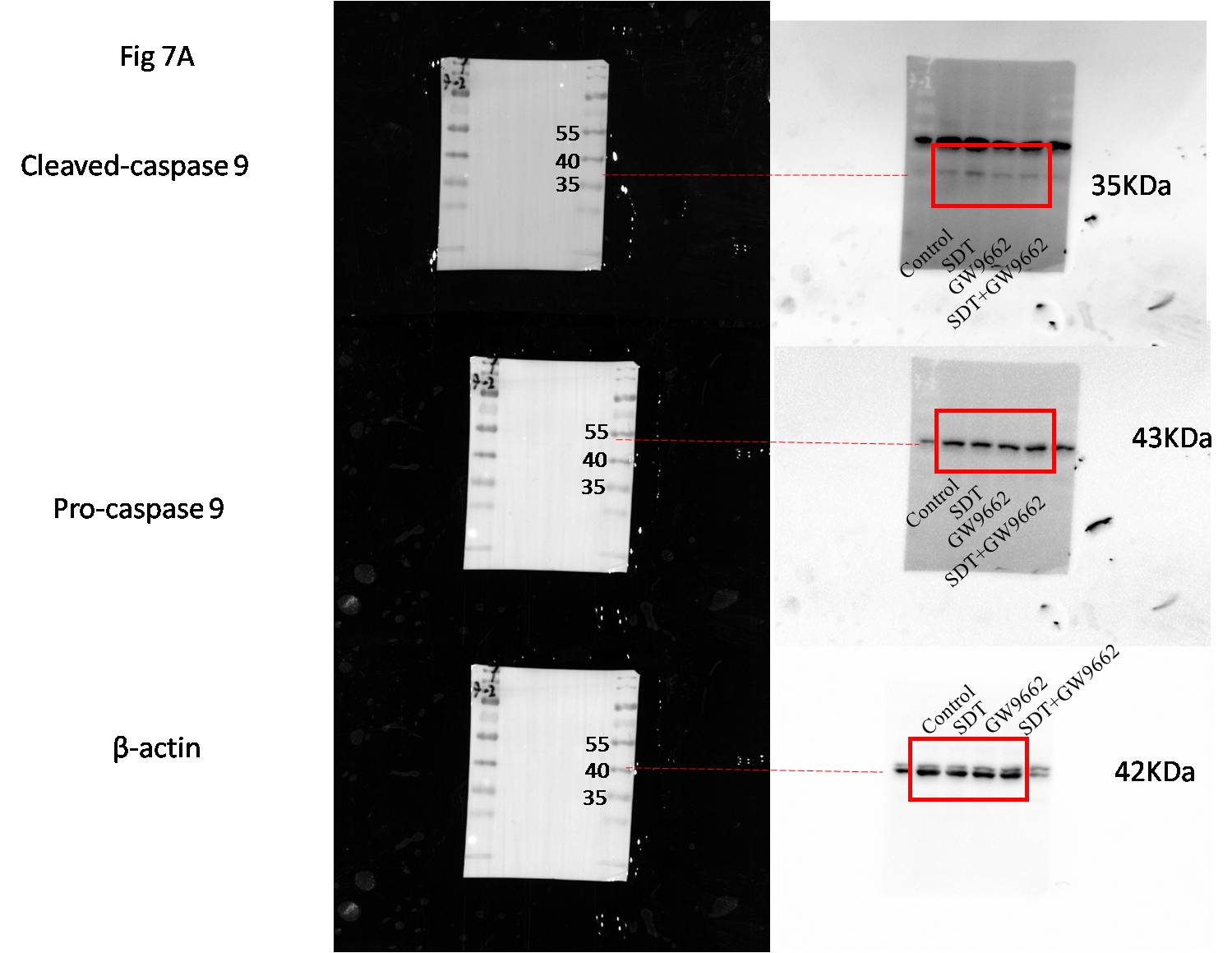
**

**
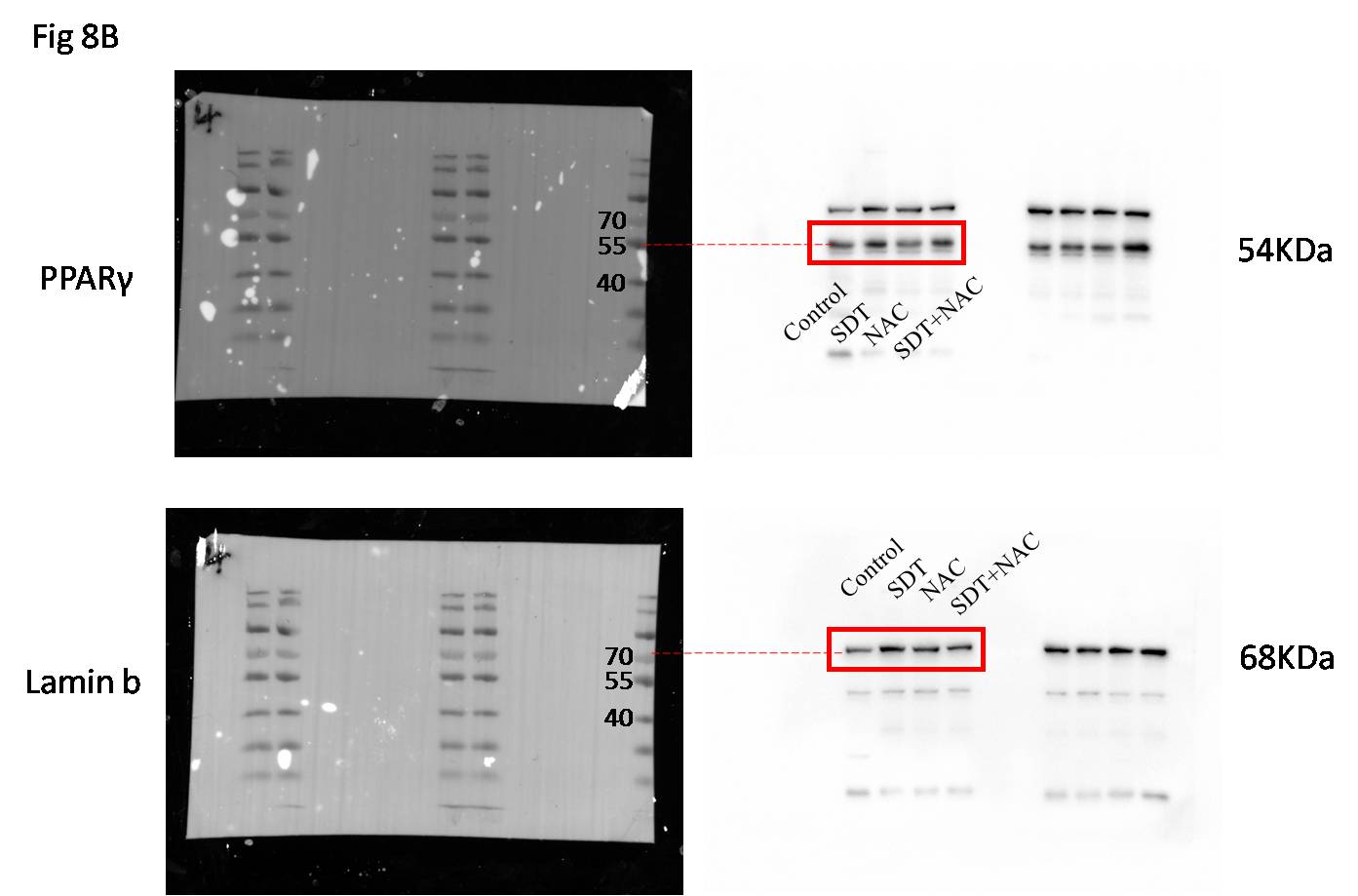
**

**
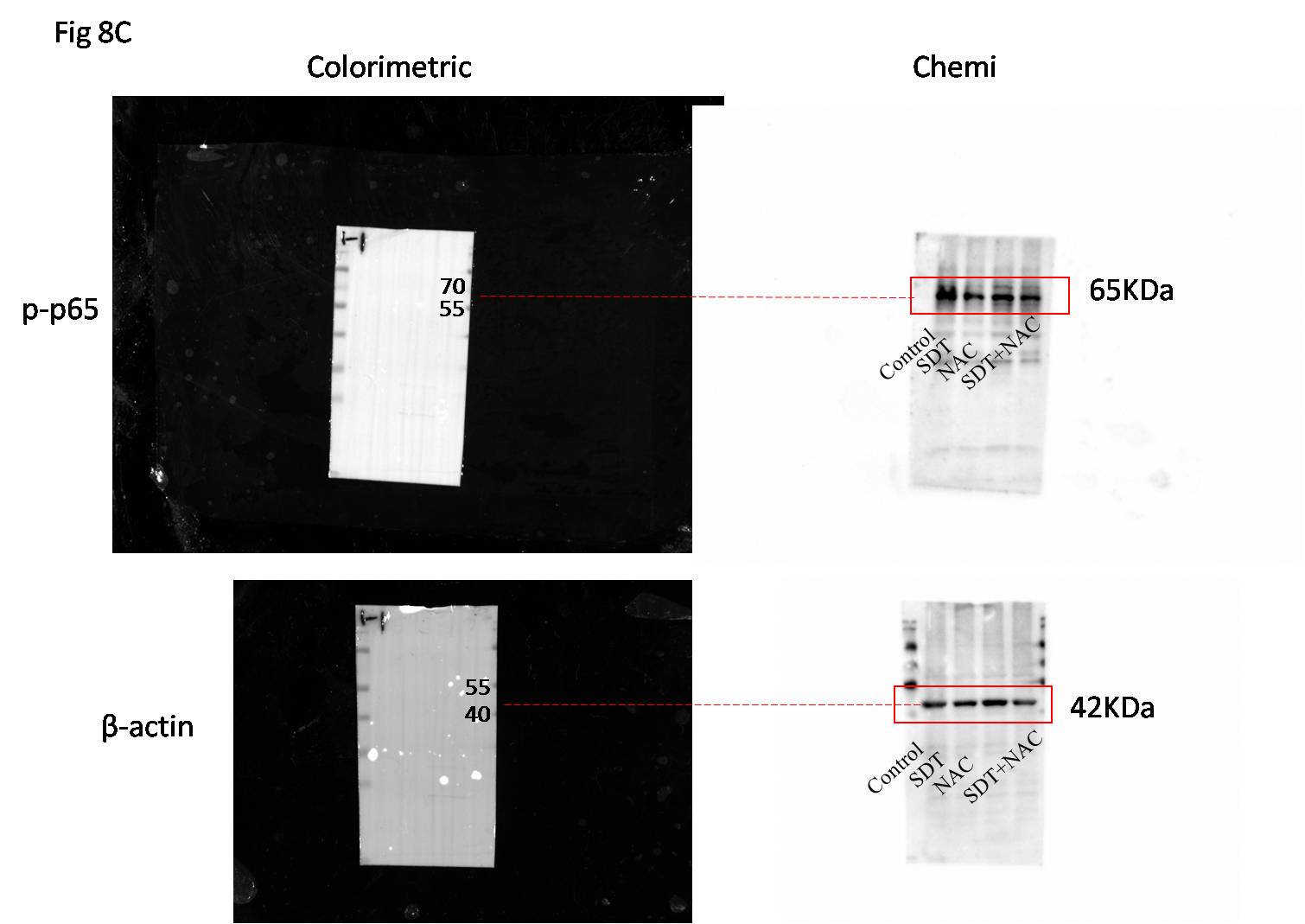
**

**
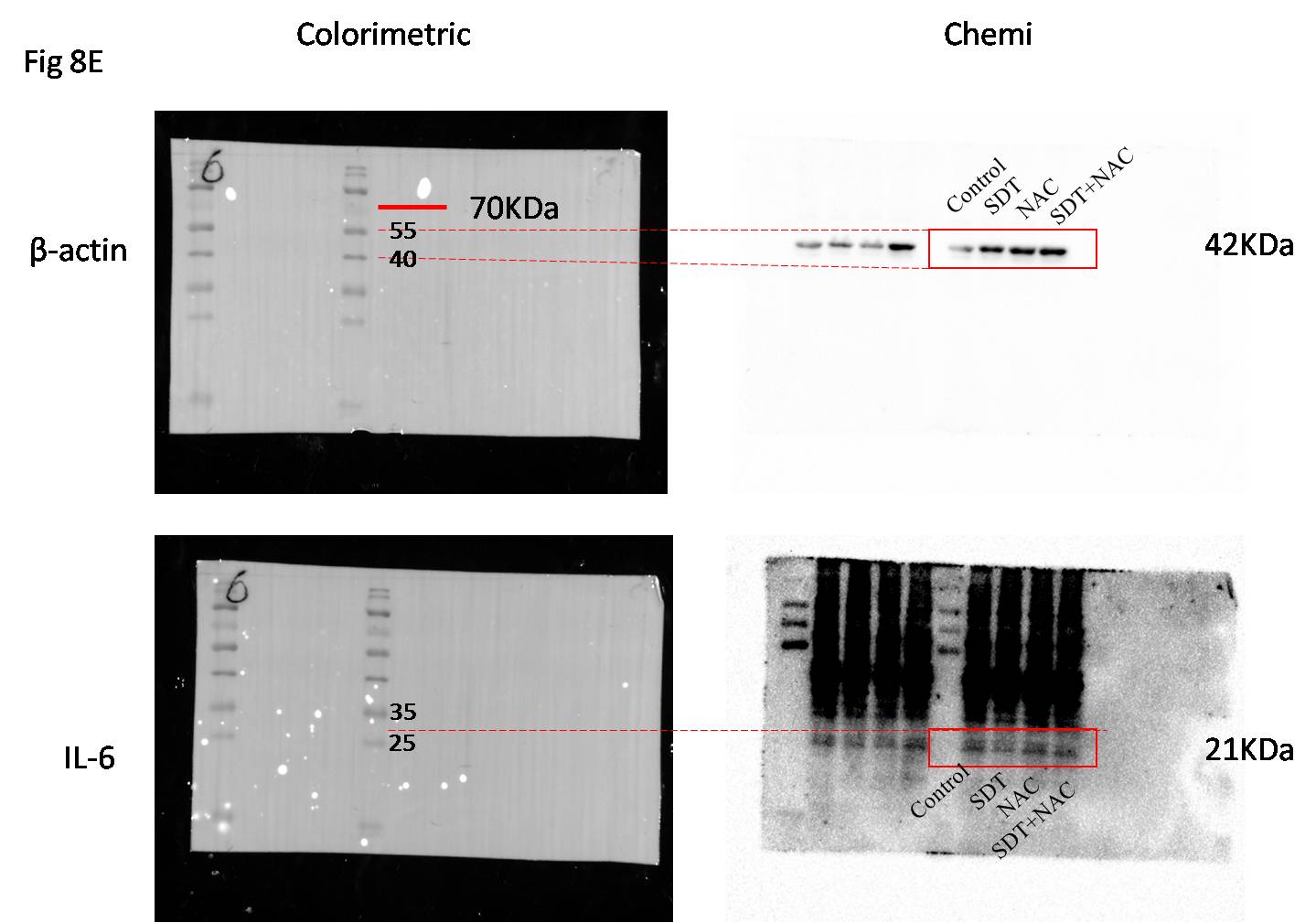
**

**
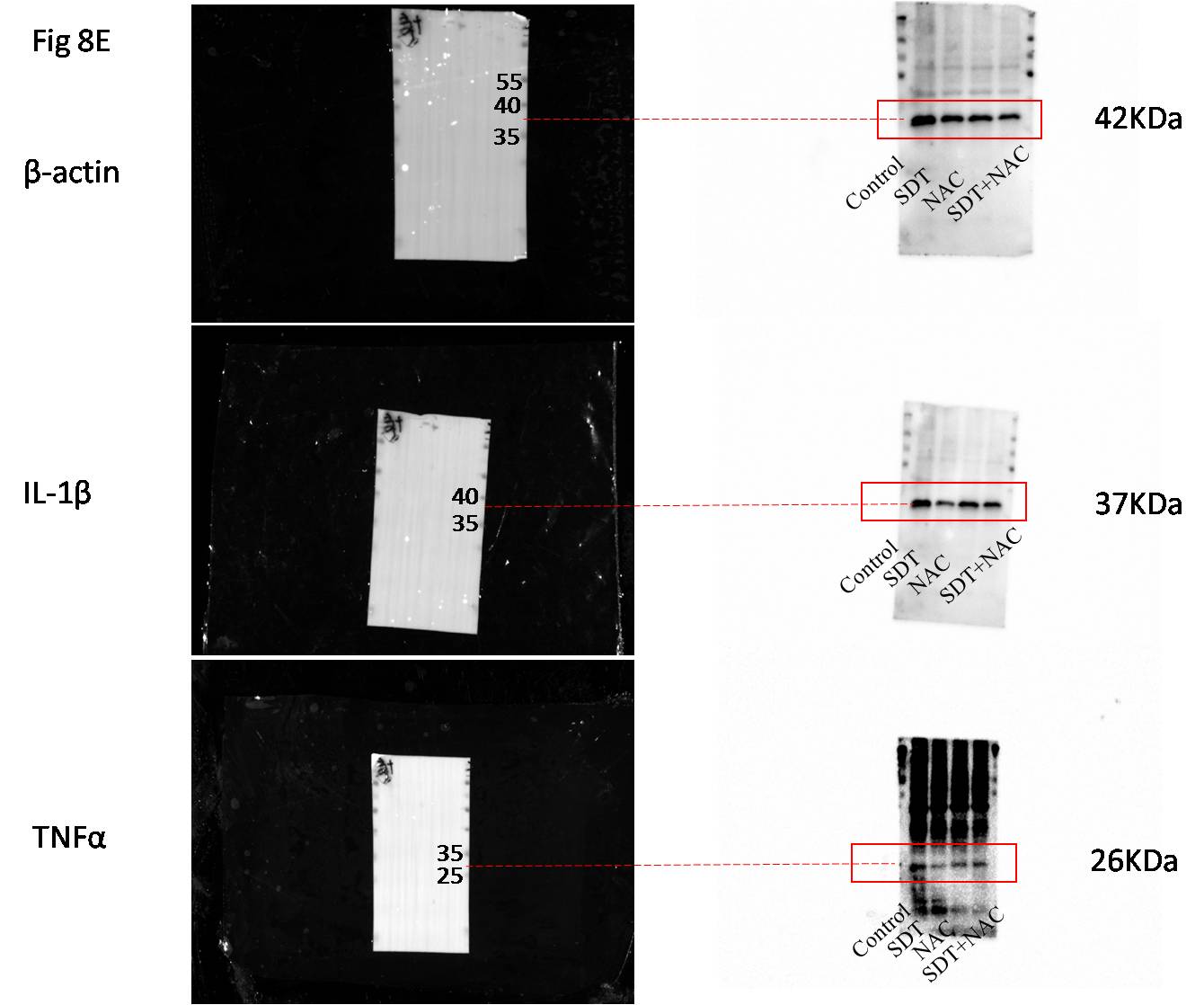
**

**
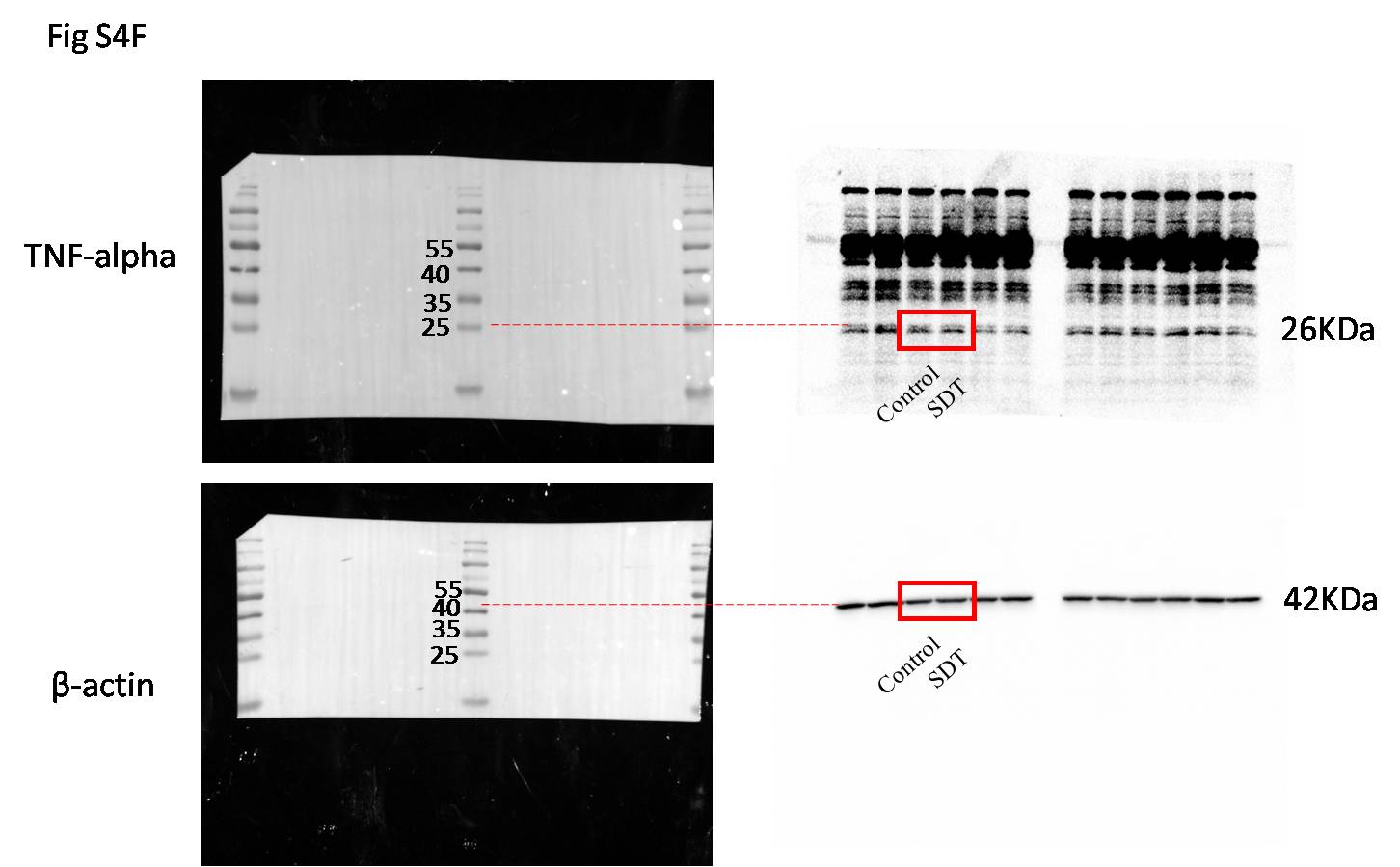
**

**
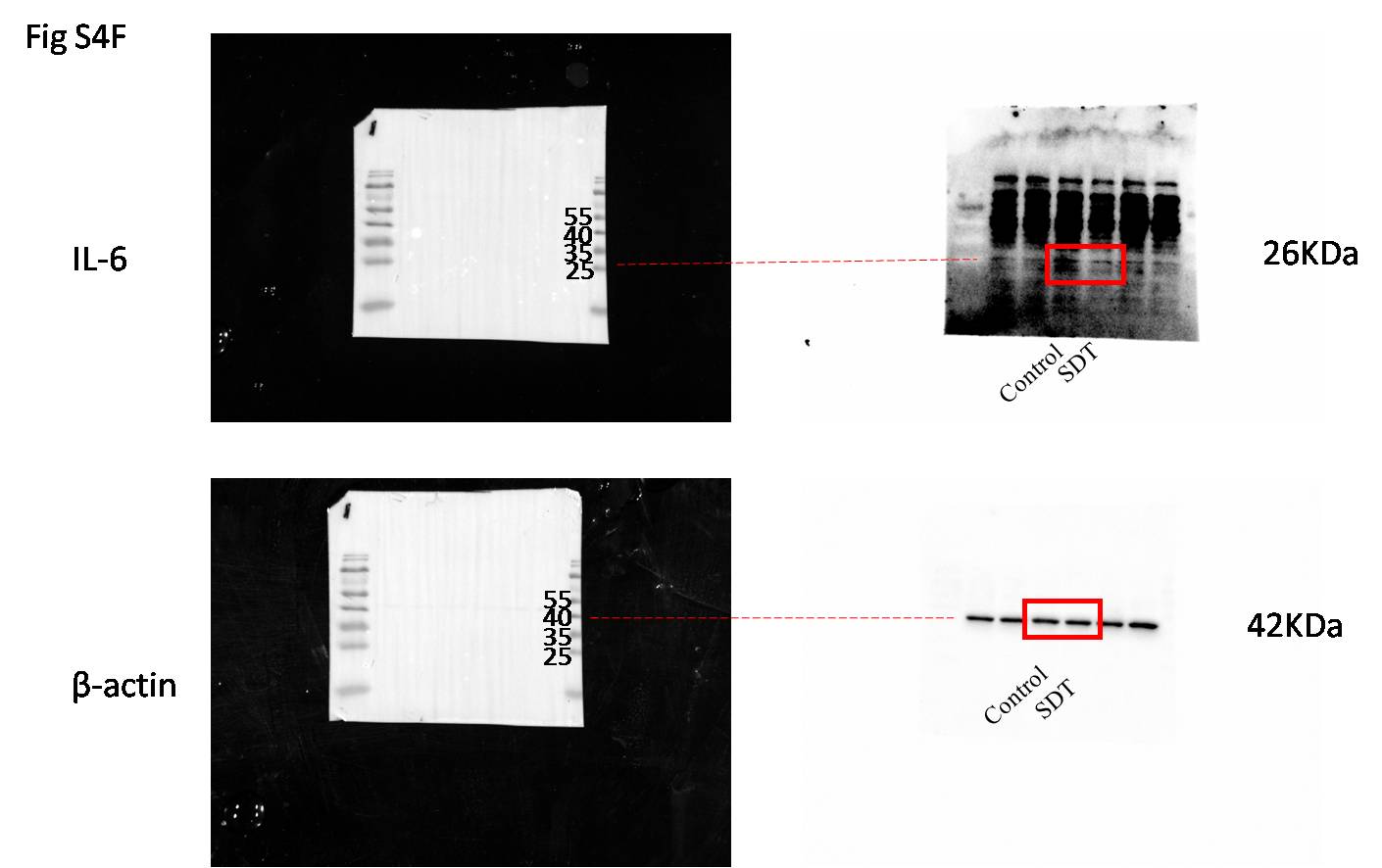
**

**
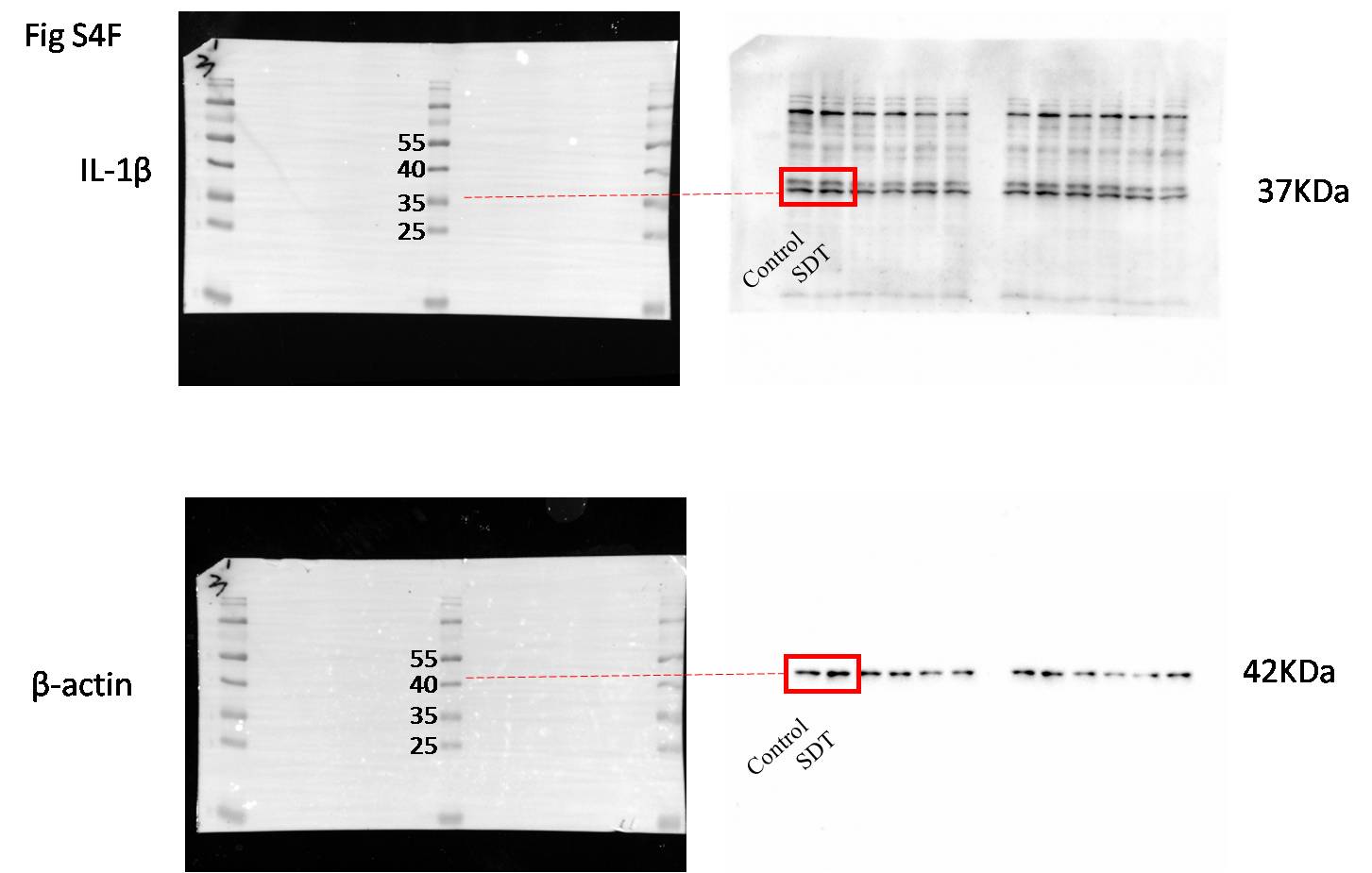
**

**
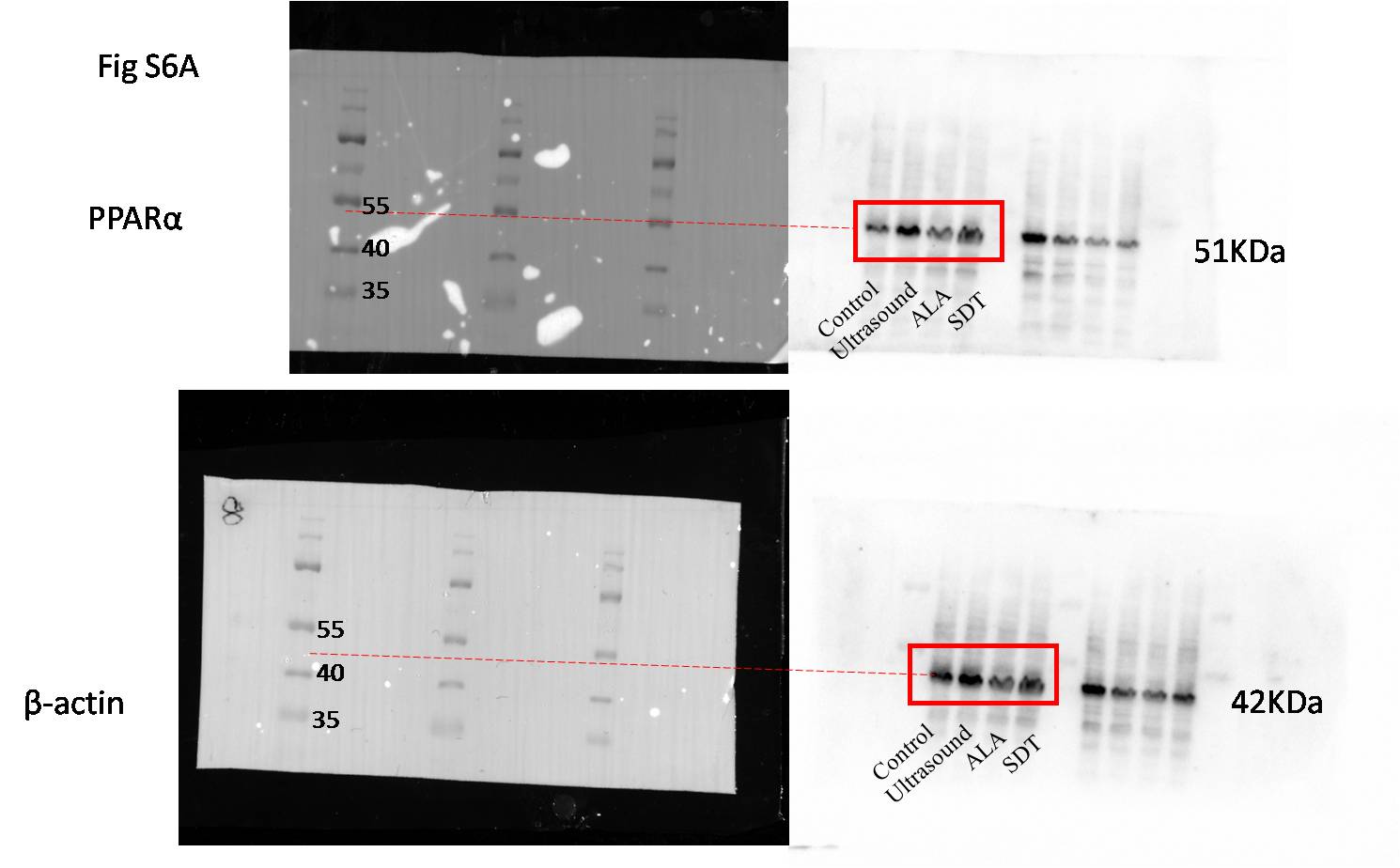
**

**
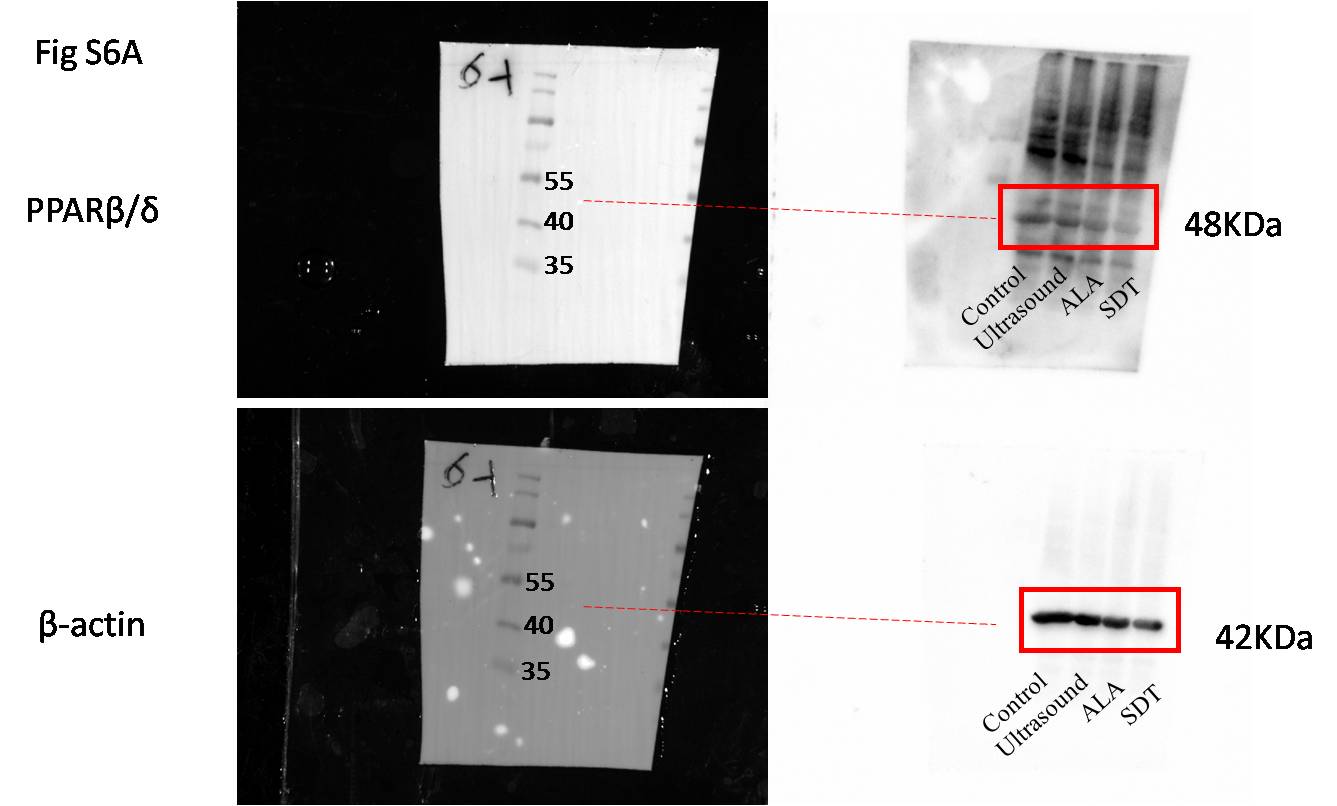
**

**
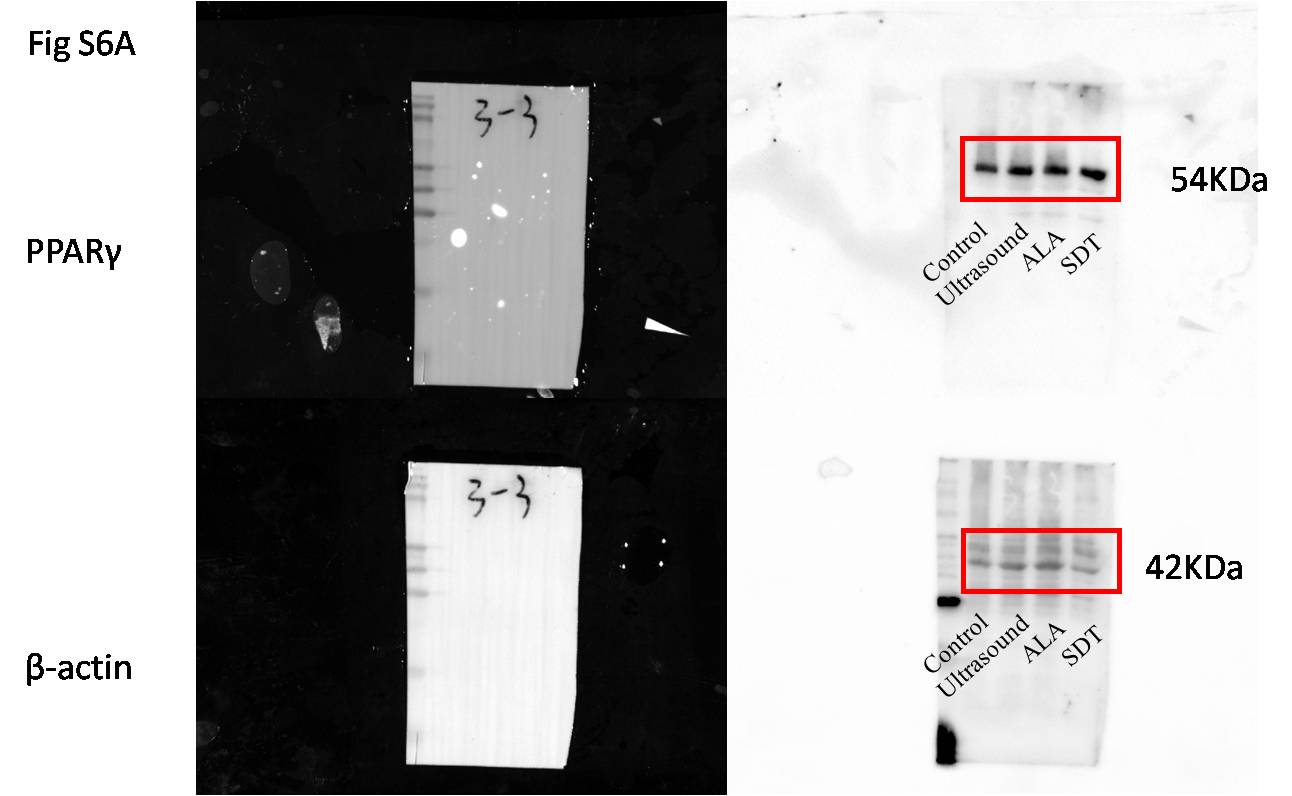
**

**
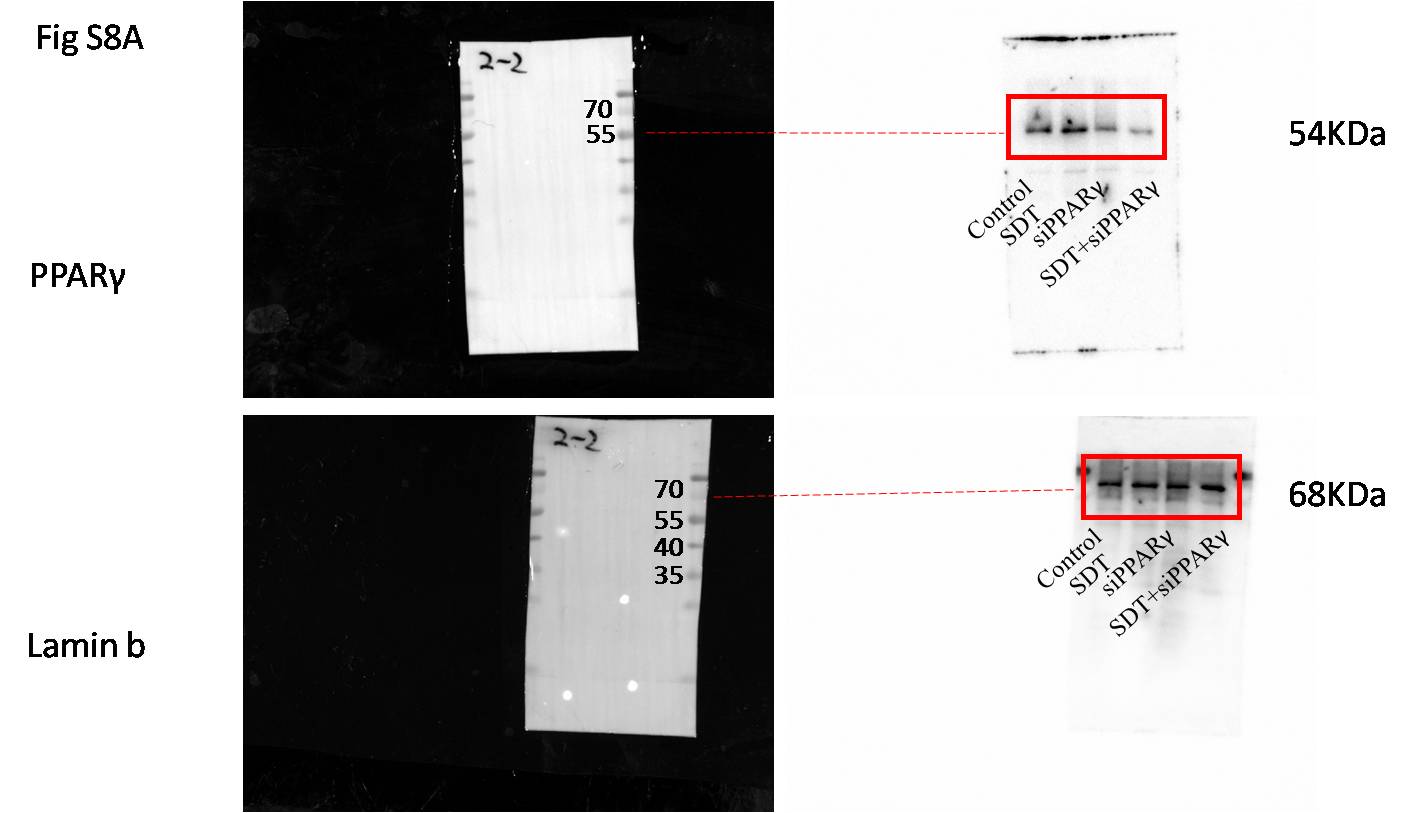
**

**
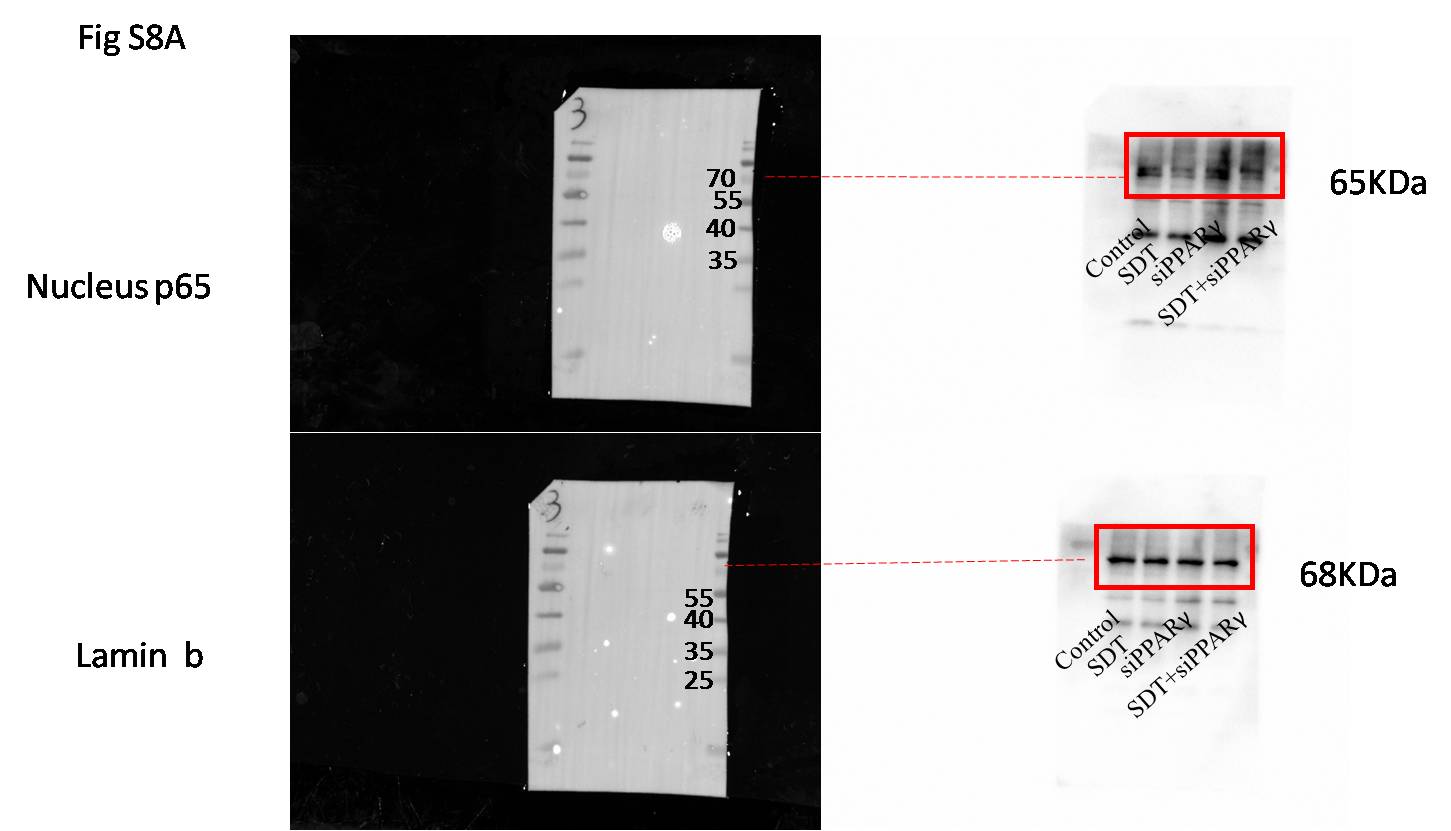
**

**
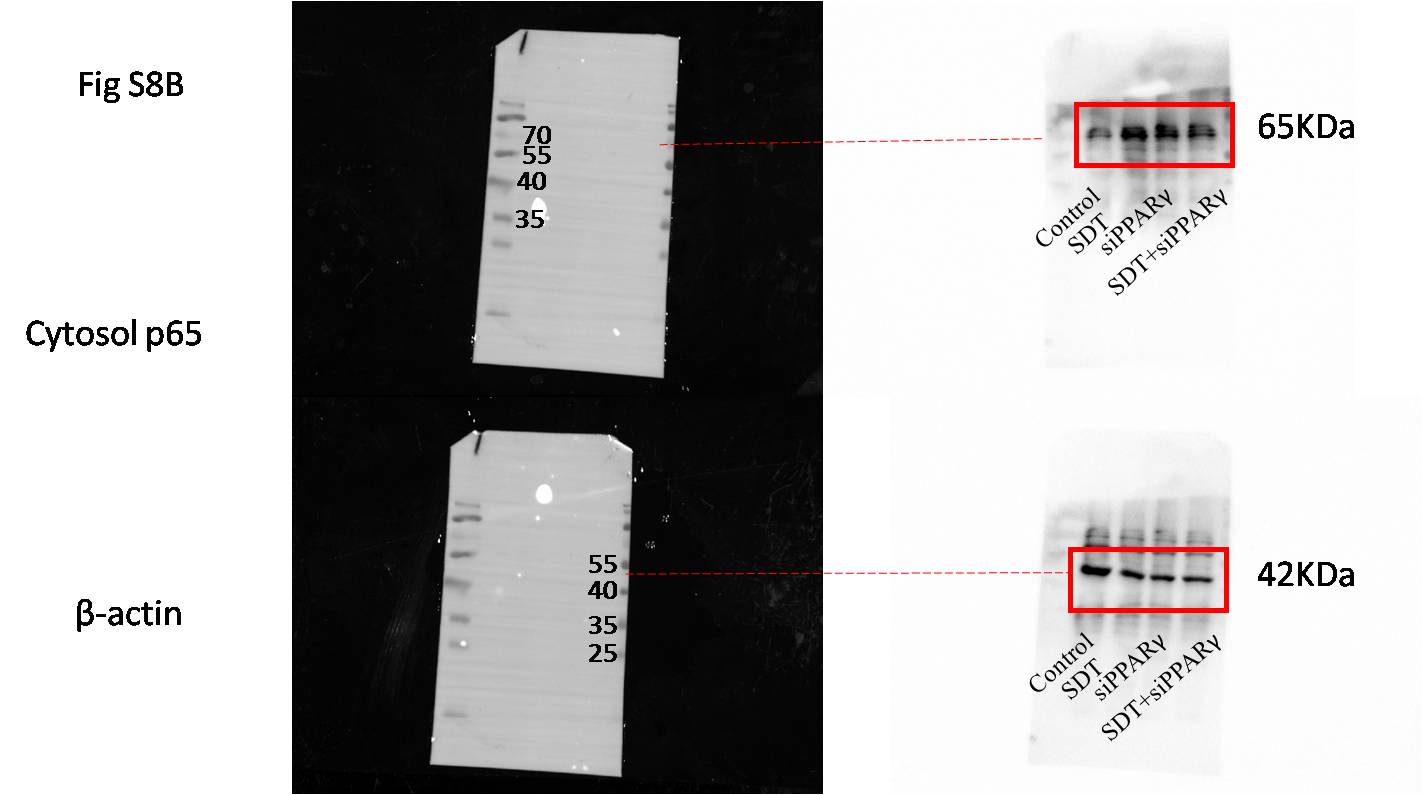
**

**
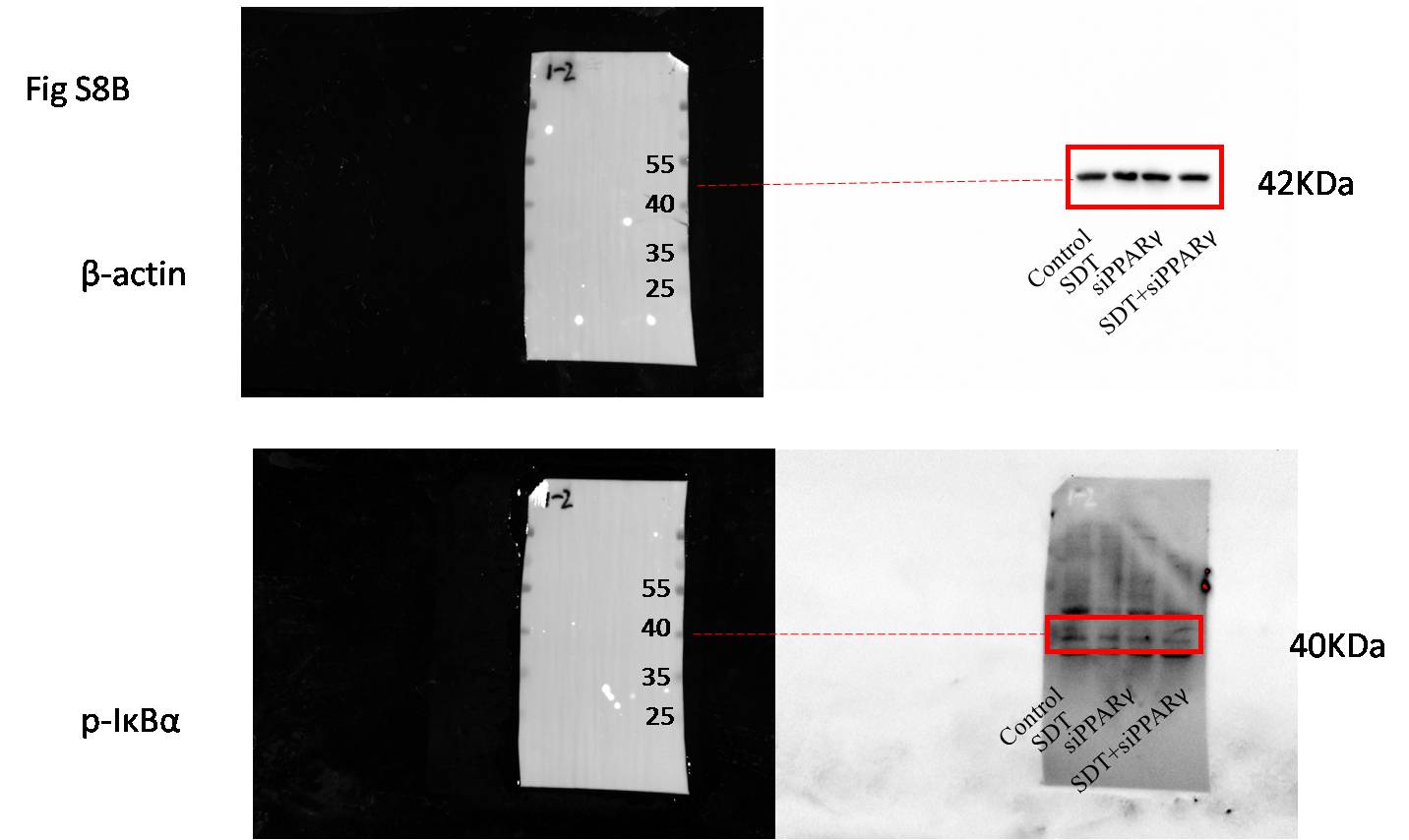
**

**
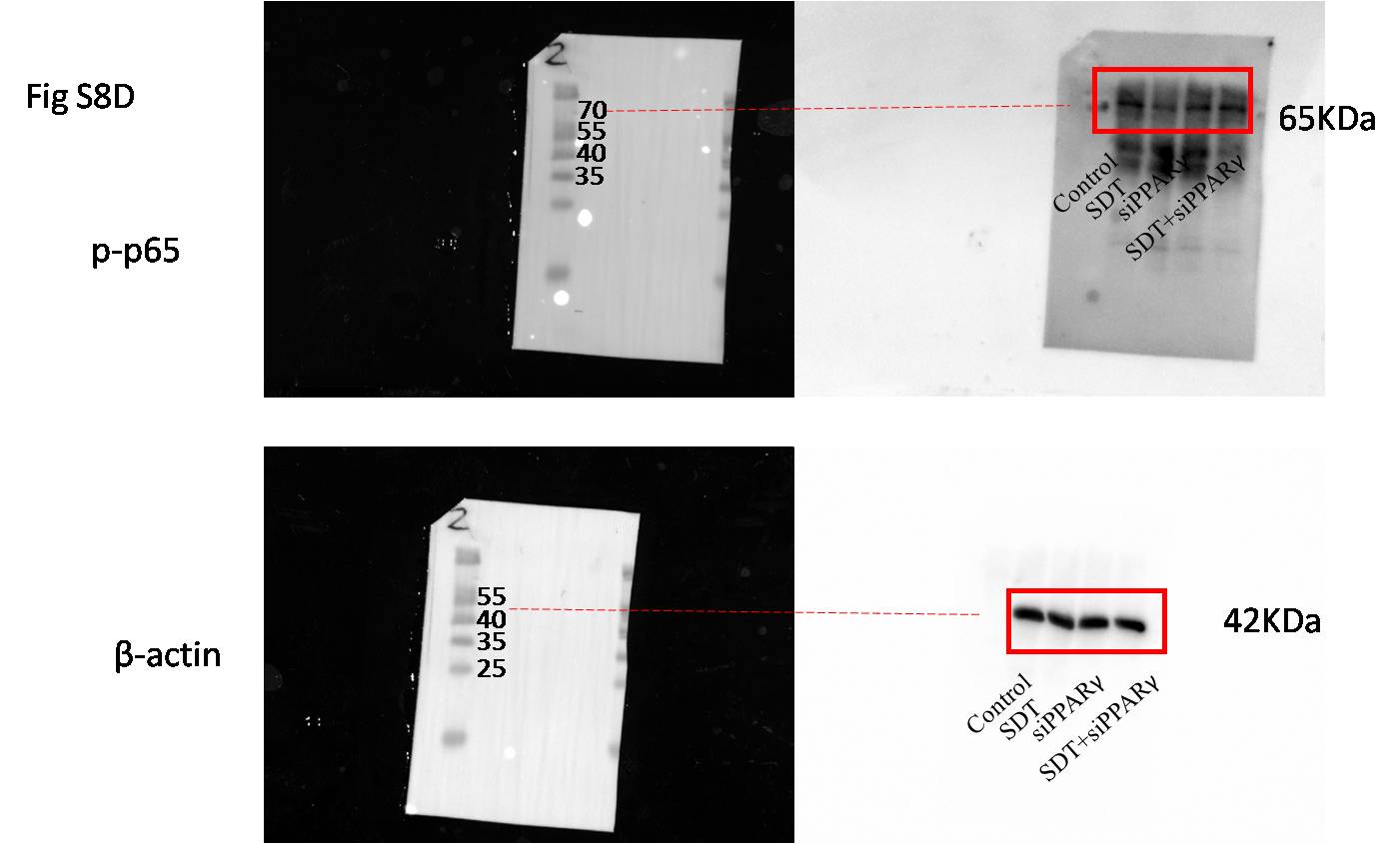
**

**
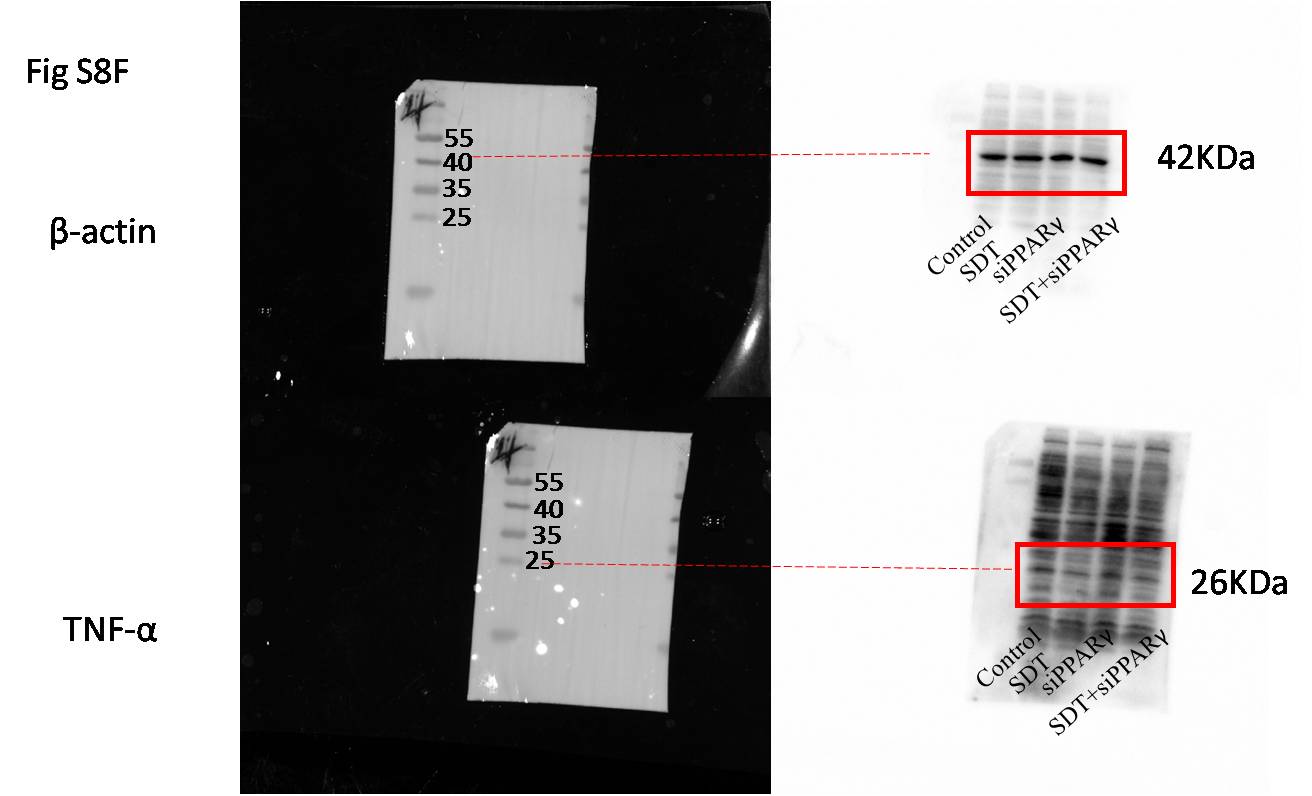
**

**
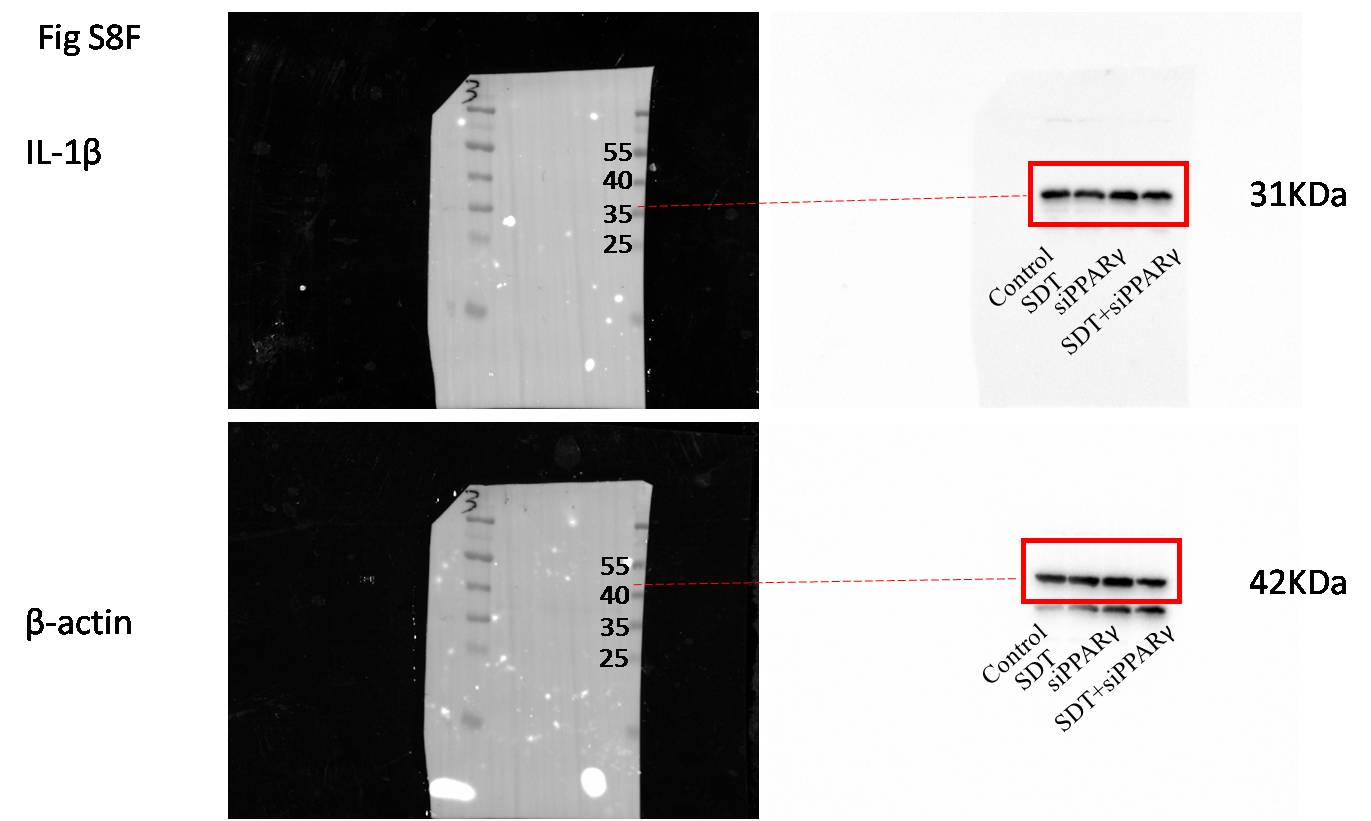
**

**
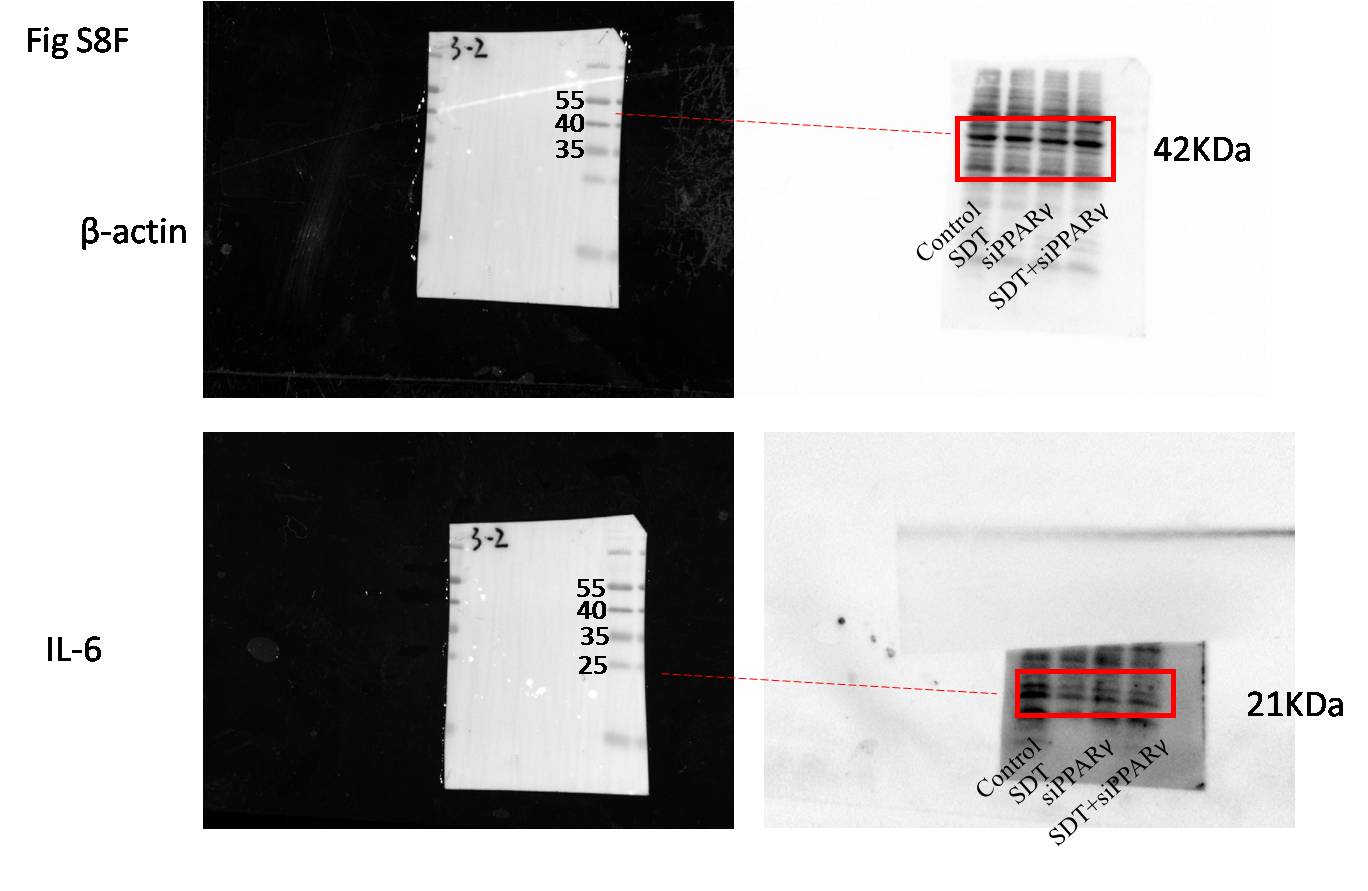
**

**Raw data**

**Fig 1B**

| **Control** | **12 h** | **24 h** | **48 h** | **72 h** |
| --- | --- | --- | --- | --- |
| \| 14.031790 \| \| --- \| \| 17.940170 \| \| 31.120480 \| \| 38.180400 \| \| 48.892270 \| | \| 86.321370 \| \| --- \| \| 86.399480 \| \| 92.960610 \| \| 97.639660 \| \| 101.421700 \| | \| 124.681800 \| \| --- \| \| 126.577700 \| \| 130.987700 \| \| 134.304600 \| \| 145.507000 \| | \| 163.685700 \| \| --- \| \| 168.562600 \| \| 175.128200 \| \| 179.037600 \| \| 195.207000 \| | \| 339.535800 \| \| --- \| \| 362.511400 \| \| 346.474900 \| \| 424.556000 \| \| 416.286100 \| |
